# Understanding pre- and post-COVID urban neighborhood dynamics: a study analyzing georeferenced tweets for New York City

**DOI:** 10.1007/s43762-026-00293-2

**Published:** 2026-08-24

**Authors:** Marjorie Mattes, Dorian Arifi, Bernd Resch

**Affiliations:** 1 Department of Geoinformatics – Z_GIS, University of Salzburg, Schillerstraße 30, Salzburg, 5020 Austria; 2 GeoSocial Artificial Intelligence, Interdisciplinary Transformation University (IT:U), Freistädter Straße 400, Linz, 4040 Austria; 3 Center for Geographic Analysis, Harvard University, 1737 Cambridge Street, Cambridge, MA 02138 USA

**Keywords:** Geo-social media, Spatio-temporal analysis, Urban functional domains, BERTopic, Post-COVID City, New York City

## Abstract

Sudden disruptions, such as the COVID-19 pandemic, can reshape multiple dimensions of everyday urban life by affecting social interactions, mobility patterns, and the use of urban spaces. Previous research efforts trying to understand the consequences for citizens relied on top-down proxies such as mobility traces, transit usage, or aggregate economic indicators. While these capture where and how much people move, they do not create new insights into how different domains of everyday urban life are perceived, discussed, and coped with over time.

This study investigates how geotagged Twitter data can be used to classify posts into predefined urban functional categories and analyze their temporal and spatial dynamics in New York City from 2018 to 2022. The research focuses on five key domains: Transportation, Retail Activity, Cultural/Social Activity, Healthcare, and Work/Remote Work. To classify content related to these categories, a methodological workflow combining Wikipedia-derived keyword filtering with BERTopic-based modeling was developed.

Temporal and spatial analyses of category-related activity reveal distinct patterns of intensifications and declines, particularly within Transportation and Cultural/Social Activity. Deviations from long-term baseline shares illustrate localized disruptions and partial recoveries in these categories, while domains with sparse representation, such as Healthcare and Work/Remote Work, display fragmented patterns that limit interpretability.

The study demonstrates the potential and constraints of using geotagged social media as a complementary source for understanding urban behavioral change. While representation biases and data sparsity remain challenges, the developed workflow offers a means to trace spatial and temporal shifts in selected aspects of urban life, particularly during large-scale events such as the COVID-19 pandemic.

## Introduction

Cities, regardless of their size, geography or level of development, share organizational, social and economic characteristics and evolve as complex systems shaped by interdependent social, infrastructural and spatial processes (Batty et al., 2018; Bettencourt, 2013; Bettencourt et al., 2013; Resch et al., 2012). However, as highlighted by Batty et al. (2022), such complex systems are also highly sensitive toward external disruptions as for instance in recent years the COVID-19 pandemic. At the same time, cities are capable of adapting to the constraints and implications that sudden disruptions impose on urban life and patterns of social interaction. In particular, such disruptions can reshape multiple dimensions of everyday life, ranging from remote work and commuting practices to the reconfiguration of retail and leisure spaces, as well as shifts in digital communication (Bandarin et al., 2021; Batty et al., 2022; Florida et al., 2021; Glaeser, 2022; Megahed & Abdel-Kader, 2022; Rao et al., 2022).

In this regard, the concept of the Post-COVID City (Batty et al., 2022) provides a useful framework for interpreting these transformations and identifying key functional domains that were most affected during and after the pandemic like mobility, healthcare, retail, work and cultural and social activity. Yet much of the empirical evidence used to assess post-pandemic urban change relies on top-down proxies such as mobility traces, transit usage, or aggregate economic indicators, which capture where and how much people move, but provide limited insight into how different domains of everyday urban life are perceived, discussed, and socially manifested over time (Buehler et al., 2025; Ziedan et al., 2023).

Moreover, geotagged social media posts have emerged as a valuable bottom-up data source for studying urban dynamics because they span spatial, temporal, and semantic dimensions of human activity, enabling researchers to infer both where and how people engage within urban space. Accordingly, geo-social media data have been shown to reveal characteristic spatiotemporal patterns of everyday urban activities and as such can be leveraged to infer functional uses of places (e.g., leisure, shopping, mobility) that align with underlying land-use categories when traditional data are unavailable or slow to obtain (Kozlowska & Steinnocher, 2020; Niu & Silva, 2023). Large-scale analyses further demonstrate that spatial and temporal clusters extracted from geotagged posts can uncover demographic differences and activity purposes when integrated with ancillary geographic information, highlighting fine-grained urban activity signatures across populations (Crivellari & Resch, 2022; Niu & Silva, 2023). Beyond functional inference, geo-social media data is also capable of capturing human expressions and sentiments tied to specific places or timeframes, enabling the mapping of emotion and sentiment profiles in urban environments (Niu et al., 2025; Resch et al., 2016).

During the COVID-19 pandemic in particular, geo-social media data from platforms such as Twitter (now X) has been shown to capture behavioral adaptations and shifts in everyday routines across multiple functional domains of urban life, including mobility, remote work, COVID-19-related healthcare discourse, and social distancing practices (Feng & Zhou, 2022; Huang et al., 2020; Xu et al., 2020; Xue et al., 2020). However, comparatively fewer studies adopt a unified, inner-urban perspective that systematically tracks multiple functional domains of everyday urban life, such as leisure, retail activity, mobility, healthcare, and work, across pre-pandemic, pandemic, and post-pandemic phases using a consistent Twitter-based classification framework.

Accordingly, to address this gap in the literature, this study is guided by the following research questions:

- (RQ 1) How did Tweet activity around different categories of urban life in New York City change between pre-pandemic, pandemic and post-pandemic periods?
- (RQ 2) How do the spatial distributions of Tweet activity change across pre-pandemic, pandemic, and post-pandemic periods, and where do spatial clusters emerge?

To answer these questions, this study proposes a methodology combining keyword filtering and BERTopic-based approach to identify Tweets related to the main functional domains of urban life and to analyze how these discussions manifest spatially and temporally. Furthermore, this work develops and evaluates a methodological workflow for filtering and classifying geolocated Twitter data to capture category-related content within the urban life domains Transportation, Retail Activity, Cultural/Social Activity, Healthcare and Work/Remote Work. Building on this, we then examine how different geo-social media activity across different categories changed in New York City between pre-pandemic, pandemic, and post-pandemic periods. Lastly, we also investigate to what extent deviations from long-term baseline shares reveal spatially clustered disruptions in geo-social media topics and link them to shifts in urban activity.

## Related work

### Urban functional domains and social media

The theoretical shift initiated by Batty et al. (2022) towards understanding the Post-COVID City requires a closer examination of specific urban functions, including transportation, economic activity, cultural and social life, working life, and healthcare. Since these domains were affected differently by the COVID-19 pandemic, their analysis requires data sources that capture not only aggregated urban change, but also more fine-grained spatial, temporal, and thematic patterns. In this context, researchers have increasingly turned to digital footprints from geo-social media as a complementary source for studying urban dynamics (Crooks et al., 2015; Miller & Goodchild, 2015; Niu & Silva, 2020; Yang & Liu, 2022).

In the domain of mobility, social media has been widely used to track changes in travel behavior. Huang et al. (2020) utilized geotagged Twitter data to reveal significant changes in human mobility dynamics during the pandemic. Similarly, Jiang et al. (2021) leveraged Twitter data to analyze spatiotemporal mobility patterns across land-use types in New York City, demonstrating how activity around transportation hubs diverged from residential areas, while Rajput et al. (2022) identified broader mobility shifts using aggregated Facebook movement data. Xu et al. (2020) further demonstrated that geolocated Tweets can serve as a proxy for social distancing compliance, showing substantial reductions in user travel following the implementation of social distancing policies across US states.

Social media has also been used to examine cultural and social engagement in urban environments. Huang et al. (2022) used Twitter data to capture public values towards specific parks as well as changes in park use during the pandemic. Reuschke et al. (2021) mapped the spatial footprint of the creative economy, showing that creative work and social interaction are not confined to city centers but are spatially dispersed. Niu and Silva (2023) examined spatial and temporal patterns of urban activities across demographic groups using geotagged Twitter data, while Niu et al. (2025) extended this work by mapping emotional expression in urban environments and linking spatial disparities in emotion to environmental and socio-economic contextual factors.

Other functional domains have also been studied using social media data, although often in isolation. In the retail activity domain, Li et al. (2023) analyzed social media sentiment towards major retailers to track shifts in service quality perception and consumer concerns related to supply shortages and safety measures during the pandemic. Regarding working life, Feng and Zhou (2022) quantified shifts in professional routines by analyzing hourly tweeting gaps between weekdays and weekends during the pandemic, thereby demonstrating the utility of social media as a proxy for work engagement and working-from-home behavior.

In the healthcare field, Xue et al. (2020) applied unsupervised machine learning to millions of Tweets to identify key themes such as public health measures and disease progression, while also quantifying dominant sentiments such as fear and anticipation. Similarly, Umair and Masciari (2023) used a deep learning model to classify sentiments towards COVID-19 vaccines based on Tweets, revealing large-scale vaccine hesitancy. Beyond this, geo-social media signals have also been shown to reflect spatially varying COVID-19 infection patterns across regions with differing political orientations or socio-economic contexts (Arifi et al. 2025a, 2025); Hanny et al., 2025).

Taken together, these studies demonstrate the value of social media for analyzing individual urban functional domains. However, most existing work focuses on one specific domain, such as mobility, parks, healthcare, or retail, rather than examining multiple domains within a unified intra-urban framework. Consequently, there remains a need for approaches that can jointly capture different dimensions of urban life and compare their spatial and temporal dynamics across pre-pandemic, pandemic, and post-pandemic periods. Addressing this gap requires not only suitable geo-social media data, but also methodological approaches capable of transforming noisy and unstructured text into interpretable, category-specific urban indicators.

### Methodological approaches for topic identification

Extracting meaningful patterns from unstructured social media text presents significant methodological challenges. Probabilistic topic modeling approaches such as Latent Dirichlet Allocation (LDA) (Blei et al., 2003) have been widely applied in urban studies to identify topical patterns related to tourism, disasters, and daily activity from large text corpora such as online reviews and social media data (Guo et al., 2016; Lansley & Longley, 2016; Resch et al., 2018). However, such approaches are known to struggle with the short, noisy, and sparse nature of Tweet data (Chen et al., 2019; Egger & Yu, 2022).

To address these limitations, recent studies have increasingly explored more advanced NLP approaches for identifying meaningful topics in social media data. For example, De Sabbata et al. (2023) demonstrated the utility of different NLP models for distinguishing everyday-life social media content from event-related content in the city of Leicester. Similarly, G. Almatar et al. (2020) assessed the primary topics of interest in Tweets during a typical week in Kuwait and analyzed their spatiotemporal distributions. These studies highlight the potential of NLP-based approaches for urban analysis, but they also underline the difficulty of producing stable and interpretable topic structures from short and heterogeneous social media texts. Direct keyword filtering offers a simpler and more transparent alternative and has been used to retrieve domain-relevant Tweets in disaster and pandemic contexts (Arifi et al., 2025a, 2025; de Albuquerque et al., 2015). However, keyword-based approaches remain prone to ambiguity when categories overlap semantically. To reduce subjectivity in topic-related content identification, researchers increasingly leverage external knowledge sources such as Wikipedia, either to compute keyword lists that define specific topics (Gabrilovich & Markovitch, 2007; Rath & Chow, 2022; Wu et al., 2017) or to derive weighted keyword lists directly from Wikipedia articles using TF-IDF for document classification (Biuk-Aghai & Ng, 2014). This logic conceptually underpins the keyword generation step presented in this study.

However, neither keyword filtering nor unsupervised topic modeling alone is well suited for classifying Tweets into multiple predefined functional categories simultaneously. Keyword filtering can retrieve thematically relevant content but cannot reliably resolve semantic overlaps between categories. Conversely, applying topic modeling directly to large and noisy Twitter datasets can be computationally demanding and may produce incoherent or weakly interpretable clusters (De Sabbata et al., 2023; Egger & Yu, 2022). This limitation is particularly relevant for the present study, which aims to classify Tweets across several overlapping urban domains within a consistent intra-urban framework.

This study therefore proposes a hybrid framework that combines Wikipedia-derived keyword filtering with BERTopic-based semantic clustering (Grootendorst, 2022) as a refinement step. BERTopic was selected over comparable embedding-based topic modeling approaches such as Top2Vec because it provides greater flexibility for short-text applications, shows higher stability across domains, and uses a class-based TF-IDF procedure that generates clearer and more interpretable topic representations. This choice is supported by the comparative review of Egger and Yu (2022), who highlight BERTopic’s advantages in producing coherent and interpretable topics from Twitter data compared with several alternative topic modeling approaches. By combining transparent keyword-based filtering with BERTopic-based topic refinement, the proposed workflow is designed to address the multi-domain, intra-urban classification challenge that existing single-method approaches cannot adequately handle.

## Data and methodology

### Study area and data

The study focuses on New York City, analyzing data at the ZIP Code Tabulation Area level. With a population of approximately 8.8 million, the city’s high density makes it particularly susceptible to the magnified effects of global crises (Ignaccolo et al., 2024). The temporal scope spans from January 1, 2018, to December 31, 2022. For analytical purposes, the dataset is structured into annual observations, while broader pandemic phases are described for contextual interpretation: the pre-pandemic period (2018 to 2019), the acute pandemic disruption marked by lockdowns and social distancing (March 2020 to early 2021) and the gradual post-pandemic recovery following the lifting of restrictions (2021 to 2022) (Cuomo 2021; Ignaccolo et al., 2024).

The primary dataset consists of approximately 15 million public, geotagged posts from the social media platform Twitter (now X), collected via the Streaming API and REST API for the years 2018 through 2022 by the University of Salzburg. The data includes textual content, timestamps and precise GPS coordinates (longitude/latitude) and therefore, encompassing point geometries only.

### Methodology

**Fig. 1:**
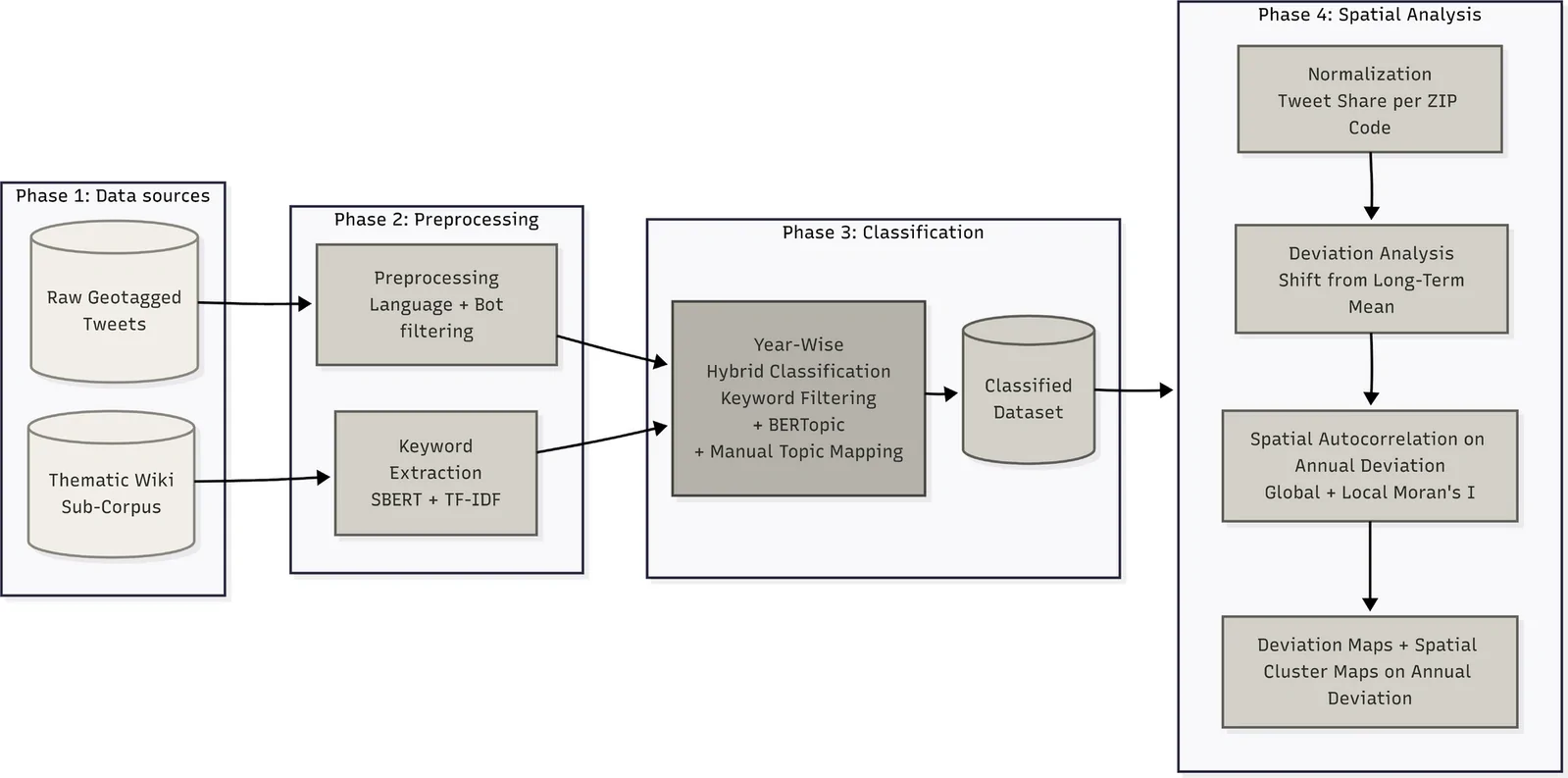
Workflow for Preparation of Twitter Data, Keyword Extraction, Tweet Classification and Spatial Analysis

The study employed a multi-stage NLP pipeline (Fig. 1) consisting of four main components. The first one focused on the preprocessing of geolocated Twitter data, including filtering, normalization and removal of organizational accounts. The second component encompassed keyword extraction using Wikipedia-based article scraping and SBERT clustering as well as TF-IDF for keyword selection. The third component included the classification of Tweets through keyword-based filtering and BERTopic-based topic modeling, followed by manual mapping of BERTopic topics to predefined urban functional categories. The final component consisted of spatial and temporal evaluation of the category-related Tweet activity for the study area.

#### Data pre-processing

As the selected NLP models are optimized for English-language text (Conneau et al., 2019), the dataset was filtered to retain only English-language Tweets. Consequently, approximately 21% of the original Tweet corpus, consisting of non-English content, was excluded from further analysis. Since the analysis focused on content generated by individual users rather than organizations, Tweets from potentially institutional or automated accounts were removed using profile metadata.

Following the heuristic-based filtering method described by Nagarkar et al. (2020), a list of keywords (“news”, “radio”, “journal”, “jobs”, “tmj_”, “tmj”, “511”, “traffic”) was created to exclude accounts with matching terms. These keywords were selected based on constant appearance in institutional or automated accounts through several manual inspection rounds. Lastly, the Tweet texts were preprocessed. Therefore, hashtags were tokenized, emojis were removed following standard preprocessing procedures, and all text was converted to lowercase.

#### Keyword generation via Wikipedia

Our approach is conceptually based on previous research by Biuk-Aghai and Ng (2014), who established a methodology for topic representation by extracting weighted keywords from relevant Wikipedia articles using TF-IDF. Hence, we utilized a domain-specific Wikipedia sub-corpus to identify high scoring terms associated with the five functional categories. Therefore, we selected seed Wikipedia articles related to the functional categories (Table 1) as entry points. Using the BeautifulSoup Python package (Richardson, 2024), we scraped linked articles and stored them into corresponding lists to form a specialized corpus. A final manual curation of the article lists was conducted to ensure the relevance of the corpus for the keyword extraction by removing articles that were not related to the functional categories (e.g. country names or general unfitting articles). The extracted and curated Wikipedia articles are available in the appendices.

**Table 1:** Wikipedia seed articles, article counts and keyword counts per category

Category	Used category-related main articles for scraping	Count of Wikipedia articles used for keyword extraction	Initial keyword pool (unique, TF-IDF ≥ 0.02)	Count of Final keyword set per category
Healthcare	Healthcare COVID-19	146	535	220
Work/Remote Work	Work_(human_activity) Job Remote_Work	60	516	135
Retail Activity	Retail	66	625	200
Cultural/Social Activity	Culture Society Entertainment	112	1113 (612 unique terms + 501 attractions)	906 (incl. Names of Monuments, Building Sites, Museums and cultural Institutions)
Transportation	Transportation Traffic	195	553	156

Next we used the pre-trained Sentence-BERT (SBERT) model all-MiniLM-L6-v2 to embed the Wikipedia article corpora for each list of articles per selected category and to identify underlying semantic structures and meaning. Subsequently, we applied a K-Means clustering algorithm to these embeddings to divide the articles into distinct, semantically coherent clusters, where each cluster represents a unique theme.

To determine an appropriate number of clusters, a sensitivity analysis was conducted testing values of K within a range of 5 to 25 across all categories. Cluster quality was evaluated qualitatively by assessing intra-cluster coherence (i.e., whether keywords within a cluster reflected a clearly identifiable theme) and inter-cluster distinctiveness (i.e., whether clusters were sufficiently differentiated from one another). Lower values of K resulted in overly broad clusters that merged distinct subdomains, while higher values fragmented coherent themes into redundant or overlapping clusters.

After partitioning the documents, we applied a TF-IDF vectorizer within each cluster to identify terms that are most relevant to the theme the cluster represents. A minimum TF-IDF threshold of 0.02 yielded most fitting keyword sets across all clusters (i.e. achieving the highest precision values), requiring only minimal manual curation in the subsequent step.

The resulting keywords were then manually reviewed to ensure domain relevance and reduce cross-category noise. The applied criteria included: (1) exclusion of generic terms that are not category-specific within the context of Tweet data (e.g., “world”, “people”, “use”), (2) removal of duplicate or synonymous terms already represented by a more specific variant, (3) exclusion of terms likely to generate false positives across multiple categories (e.g., “model”, “period”), and (4) retention of multi-word expressions where these provided more precise semantic meaning than their individual components (e.g., “home office”, “public transport”).

The final sets of keywords per category were then created by manually selecting the most representative, high-scoring terms from the 10 thematic clusters and stored in a final nested keyword list. Specific entity names that were directly extracted from Wikipedia lists of buildings, museums and parks were directly appended to the “Cultural/Social” keyword set.

We then filtered Tweets according to the existence of matching keywords within their normalized text field. Tweets that did not contain any of the keywords provided in the keyword list weren’t used in the upcoming topic modeling step. The subsequent topic modeling step was employed to resolve potential overlaps and ensure a higher classification precision.

#### Topic modeling and category assignment

Following the keyword filtering step, the resulting corpus was stratified by year (2018 to 2022) to ensure computational feasibility and to enable comparisons between different pandemic phases. Running BERTopic on the complete multi-million Tweet corpus simultaneously exceeded the available computational resources, particularly during the embedding generation and density-based clustering stages. Therefore, annual subsets were modeled independently.

We ran independent BERTopic models (Grootendorst, 2022) for each annual dataset using the default parameter settings. BERTopic internally applies UMAP (Uniform Manifold Approximation and Projection) (McInnes et al., 2018) for dimensionality reduction to improve efficiency and HDBSCAN (Hierarchical Density Based Clustering of Applications with Noise) for density-based clustering, which automatically designates noise as outliers (Campello et al., 2013, 2015). Finally, the topics are extracted using a class-based variation of TF-IDF procedure (Grootendorst, 2022; Joachims, 1997).

To speed up the dimensionality reduction and clustering step, the cuML (NVIDIA RAPIDS) version of UMAP and HDBSCAN was leveraged through GPU acceleration. A fixed random seed state (42) was set to ensure that results are reproducible across runs, as both algorithms (UMAP and HDBSCAN) involve stochastic processes. The annual BERTopic models employed the standard UMAP and HDBSCAN parameterization commonly used within BERTopic, while a custom CountVectorizer and the all-MiniLM-L6-v2 sentence embedding model were used. Specifically, UMAP was configured with 15 neighbors, five components, a minimum distance of 0.0, and cosine distance, while HDBSCAN used a minimum cluster size of 10, Euclidean distance, and the excess-of-mass cluster selection method with prediction data enabled. These parameter settings were retained to ensure consistency with prior applications of the workflow on smaller datasets (e.g., Vienna and Berlin) and to limit the proportion of Tweets assigned to the outlier class (−1), thereby prioritizing data retention. A complete overview is provided in Table 3 in Appendix.

For category assignment, each topic was represented by its five most relevant terms based on c-TF-IDF scores. Prior to manual mapping, automated approaches including keyword-only filtering and SBERT-based similarity matching were tested, but resulted in insufficient precision due to semantic overlap between categories.

Therefore, topics were manually assigned to one of the predefined functional categories based on the semantic interpretation of their top five representative terms. Topics were mapped to the category whose thematic domain best matched these terms. Topics with ambiguous or non-specific term sets were not assigned and instead classified as outliers. This mapping procedure was applied consistently across all annual BERTopic models.

To assess the quality of the category assignment, a manual precision evaluation was performed based on a random sample of 200 Tweets per category. As shown in Table 2, all categories achieved precision scores above 90%, suggesting that the combined keyword filtering and BERTopic-based workflow provides reliable classification results across the selected themes.

**Table 2:** Precision and recall values and example tweets per category

Category	Precision	1000 Tweet Sample Recall	Example Tweet Content
Healthcare	91.00%	16.67%	• *“Thank You All!! @ Brooklyn Gardens Nursing and Rehabilitation Center”* • *“Texas economy reopens amid new spike in COVID-19 cases”* • *“This virus is nothing to play with. @ New York, New York”* • *“Home or Hospital? Some reasons to consider #homebirth”* • *“I'm at The Family Health Center of Harlem in New York, NY”*
Transportation	95.50%	52.50%	• *JFK was a ghost town last week when I picked up daughter from SLC. #GladShesHome #AllGatheredIn #BeSafe @ JFK International Terminal 4*“ • “*I'm at MTA Subway—Sutphin Blvd (F)—@nyctsubway in Jamaica, NY*” • “*Toyota RAV4 uber driver [plate redacted] blocked the bike lane near 1102 Bedford Ave on January 6 and has been reported to #nyctaxi. This is in Brooklyn Community Board 03 & #NYPD79. #VisionZero #BlockedBikeNYC*”
Retail Activity	93.00%	19.23%	• “*I'm at Union Square Greenmarket—@unsqgreenmarket in New York, NY*” • “*Opening Night! Let?s take a swig!? #brewery #openingnight #party #beer @ The Bronx Brewery*” • “*Toro NYC will be CLOSED this evening for a private event! We will reopen for dinner service*” • “*3 Prong Tennis Chains Available Email Us [email address redacted] or call/text [phone number redacted]*” • *“Much needed haircut. (@ Asia Barber Shop in New York, NY)*”
Work/Remote Work	92.50%	42.86%	• *“Step into my office? @ Sant Ambroeus SoHo”* • *“What roles to hire first?”* • *“Going to work very upset tonight @ Brooklyn, New York”* • *“We’re #hiring! Read about our latest #job opening here: Coordinator [address redacted] #Clerical #NewYork, NY”*
Cultural/Social Activity	94.50%	14.79%	• “*I'm at New York Theatre Workshop—@nytw79 in New York”* • *“NYC is ready for #NYE2023 @ Times Square, New York City”* • *“Me enjoying a hearty bowl of onion soup; loaded with onions and melty cheese atop toasted baguette.”* • *Do You Love to Write? Join the LiveJournal Community and Write on Your Own Blog!”* • *“Christmas Day. Home. (@ [address redacted] in New york, NY)”*

In addition, the recall was evaluated using a manually labeled random sample of 1,000 Tweets. Among the 948 manually relevant Tweets, 520 were retained by the keyword filtering stage, corresponding to a keyword-filtering recall of 54.9%. Overall, 209 Tweets of the relevant Tweets were recovered by the final workflow, resulting in an overall workflow recall of 22.0%. The strict category-level accuracy, requiring agreement between manual and final category assignments, reached 17.7%.

Table 2 showcases how recall varied considerably across categories. Transportation and Work/Remote Work showed the highest category-specific recall values, whereas Cultural/Social Activity exhibited substantially lower recall.

#### Spatial analysis

The spatial analysis was conducted at the level of ZIP Code Tabulation Areas (ZCTAs; hereafter referred to as “ZIP codes” for brevity), with administrative boundaries sourced from the U.S. Census Bureau TIGER/Line Shapefiles (U.S. Census Bureau, 2020).

To ensure comparability across areas with uneven posting volumes, Tweet frequencies were normalized by the total number of Tweets per ZIP code. Following the approach of Kontokosta et al. (2024), we analyzed temporal dynamics by quantifying the deviation from each area’s long-term mean for the years 2018 through 2022. This allows for the detection of relative shifts regardless of absolute volumes.

To assess whether these deviations exhibit spatial structure, spatial autocorrelation was evaluated using Global and Local Moran’s I. A fixed-distance band was applied to model spatial relationships between ZIP codes, allowing for the identification of both overall clustering patterns (Global Moran’s I) and localized hotspots and outliers (Local Moran’s I).

While the subsequent Section 4.1 first provides a descriptive overview of the absolute dataset characteristics, this deviation-based metric is applied specifically in Section 4.2 to uncover spatiotemporal anomalies.

## Results

### Results of topic modeling and category assignment

Following the topic modeling procedure 24% of Tweets were retained from the 15,005,830 geolocated Tweets in the New York Metropolitan Area (3,601,399). Focusing on an area of interest encompassing the five New York City boroughs, Brooklyn, the Bronx, Manhattan, Queens, and Staten Island, and their corresponding ZIP Code Tabulation Areas (ZCTAs), the final dataset comprises 2,281,953 Tweets. These Tweets represent category-specific activity counts for the period 2018 to 2022 and correspond to approximately 15% of the original dataset. The number of category-relevant Tweets reached its peak in 2019, followed by a noticeable decline of Tweets in the following years. The general decline of geolocated Tweets was comparable to the general development of the Tweet counts in the New York Metropolitan Area (Fig. 9 in Appendix).

**Fig. 2:**
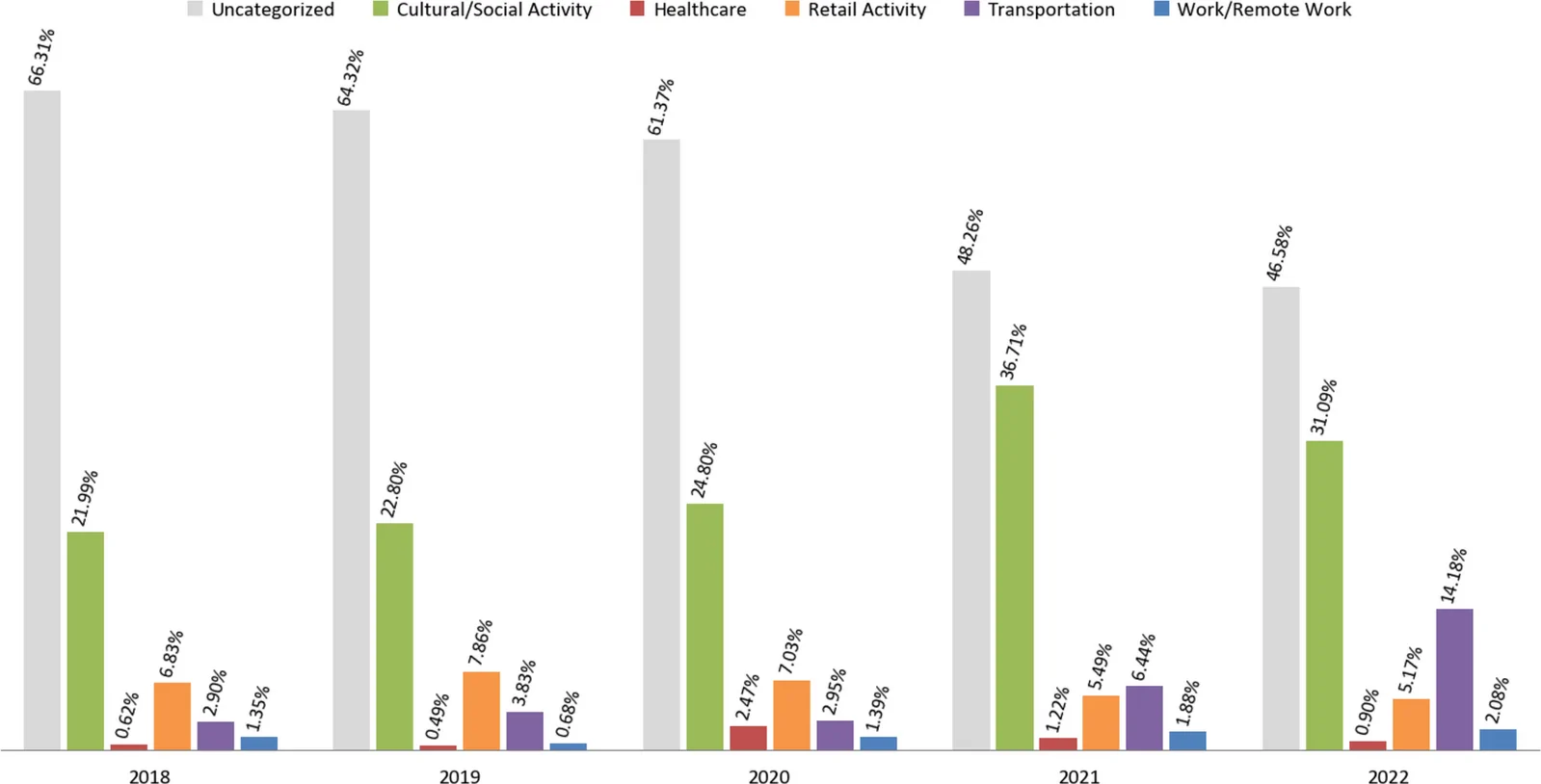
Category Shares among Category-Related Tweets in NYC

Out of the category-relevant Tweets (2,281,953), BERTopic assigned 39.28% of Tweets to distinct topics, while the remaining 60.72% were recognized as outliers. After mapping the discovered BERTopic topics to the urban categories, Cultural/Social Activity emerged as the largest share representing 21.99% to 36.71% of category-related Tweets, peaking in the year 2021 in that regard (Fig. 2). Retail Activity followed with its highest share of 7.86% in 2019 and a continuous decline down to 5.17% in 2022. Transportation shows a strong share increase from 2.90% in 2018 to 14.18% in 2022. The two remaining categories cover smaller share ranges, with Healthcare peaking in 2020 with a share of 2.5% and otherwise covering a range of 0.48 to 1.23%. Tweets classified as Work/Remote Work have their lowest share in 2019 with 0.67% and increasing up to 2.08% in 2022 (Fig. 2).

### Spatial distributions and patterns of categorized tweet content throughout the years of 2018 to 2022

#### Overview of all categories

As seen in Figs. 10 to 12 in Appendix, the spatial distribution of Tweets containing category relevant keywords shifts notably between 2018 and 2022. In the pre-pandemic years of 2018 and 2019, ZIP codes within Manhattan and adjacent areas of the Bronx, Brooklyn or Queens dominate the picture with many areas showing at least 5,000 and in some cases even more than 10,000 category-related Tweets per year. Moreover, the John F. Kennedy International Airport (JFK Airport) in southern Queens also emerges as a prominent location, while Staten Island shows higher counts close to the Upper New York Bay.

**Fig. 3:**
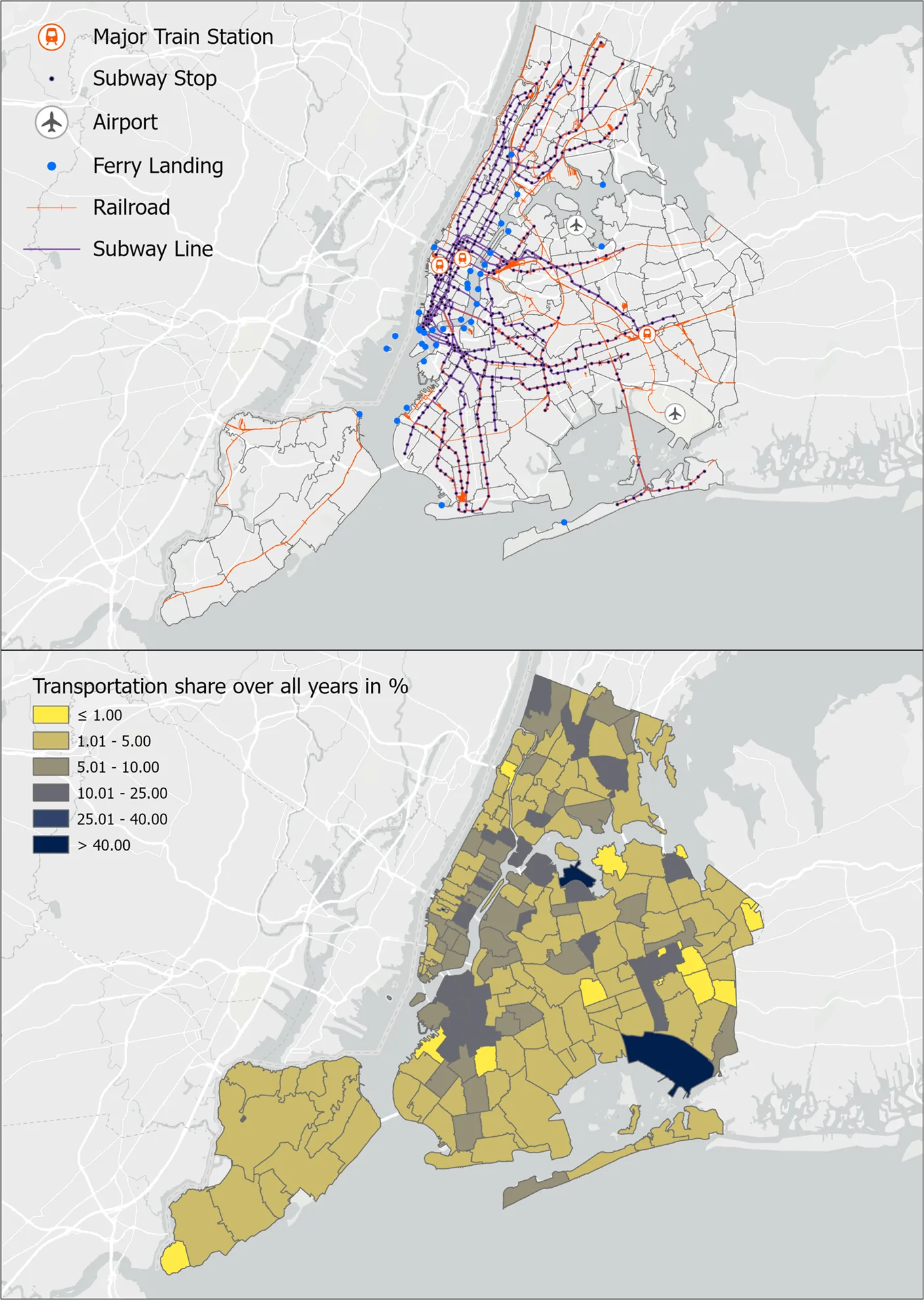
Public Transport Infrastructure NYC (NYC Office of Technology & Innovation, 2022a, b, 2025a, b) (top) and All-Year Average Share of Transportation-Related Tweets (bottom)

In 2020, Tweet volumes begin to decline, especially in areas that showed high Tweet counts in the previous year. At this point, several ZIP codes in Manhattan still exceed 5,000 geolocated and category-relevant Tweets but in the Bronx, Brooklyn, Queens, and Staten Island such counts become rare, with only a few isolated areas surpassing this threshold. This contraction of category-related Tweet activity becomes more visible in 2021, where a few ZIP codes in Manhattan and nearby parts of the Bronx, Brooklyn and Queens only exceed 1,000 Tweets, while larger parts of the city fall below. Exceptions remain visible in the area around JFK Airport, New York Upper Bay and the area around Prospect Park in Brooklyn. The overall decline continues in 2022, with Tweet counts thinning out across nearly all boroughs in New York City. Manhattan and its immediate surroundings still account for the highest Tweet count volumes, but many ZIP codes in the Bronx, Brooklyn, Queens and Staten Island do not even exceed 500 category-related Tweets (Fig. 3).

Furthermore, with respect to spatial patterns, the Healthcare and Work or Remote Work categories exhibit comparatively low Tweet volumes and correspondingly less pronounced spatial clustering relative to the other functional categories. Therefore, we focused mainly on the topics Transportation, Retail Activity and Cultural/Social Activity in the following spatial analysis. Nevertheless, the maps and analysis related to all topics can be found in the appendices (Figs. 13 to 42 in Appendix).

#### Transportation

**Fig. 4:**
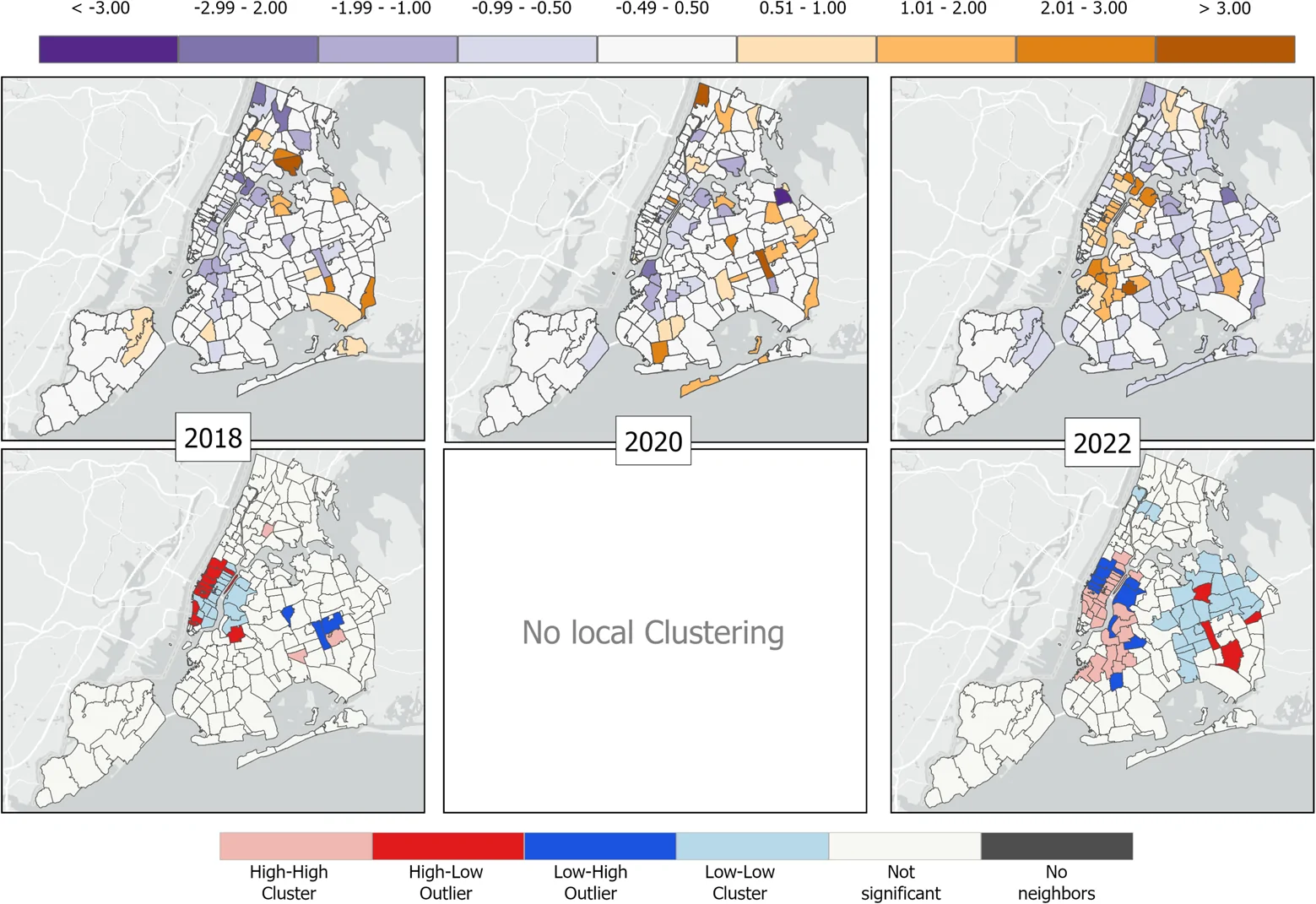
Deviations of Transportation-Related Tweets from All-Year Average (2018, 2020, 2022) and Local Moran’s I Results

In the pre-pandemic period (2018 to 2019), transportation-related Tweets were geographically dispersed, with concentrations around major infrastructure hubs like Airports in northern and southern Queens and areas in the Bronx crossing the Henry Bruckner Expressway. Notably, despite Manhattan’s density of transit hubs like Penn Station or the Grand Central Station, the borough exhibited largely below-average Tweet activity compared to the long-term average (Fig. 4). Conversely, Staten Island showed localized intensities where the Staten Island Railway and Ferry terminal are located. The Local Moran’s I analysis in Fig. 4 illustrates significant high-high and low-low clusters at the tip of Manhattan and minor parts of Queens and Brooklyn. By the onset of the pandemic in 2020, overall spatial distribution of transport-related Tweets contracted (Fig. 4). While general activity remained high around key locations like airports, the deviations reveal a more fragmented pattern (Fig. 4). In central Queens, Jamaica Station shows notable above-average activity, whereas JFK Airport, despite higher Tweet volumes, aligned with the long-term average.

During the post-pandemic phase (2021 to 2022), Transportation-related Tweet activity in 2022 was most strongly concentrated along subway corridors in Manhattan and Brooklyn, while activity levels in Queens remained below the yearly average (Fig. 4). Correspondingly, Local Moran’s I results revealed significant high-high clusters in Manhattan and Brooklyn and extensive low–low clusters in Queens and the Bronx. A small number of spatial outliers persist, including a high–low cluster around Jamaica Station and several low–high ZIP code areas in Manhattan and Queens.

Spatial autocorrelation analysis showed significant clustering in 2018 while no significant spatial autocorrelation was detected for 2019 to 2021. By 2022 however, the clustering of deviations became highly significant (Table 4 in Appendix). Further local analysis confirmed that in 2018, significant clustering mainly reflects areas of consistently below-average transportation-related Tweet activity in eastern Manhattan and northern Queens complemented by high-low outliers in northeastern Manhattan and low–high outliers around Jamaica Station in Queens.

#### Cultural/social activity

**Fig. 5:**
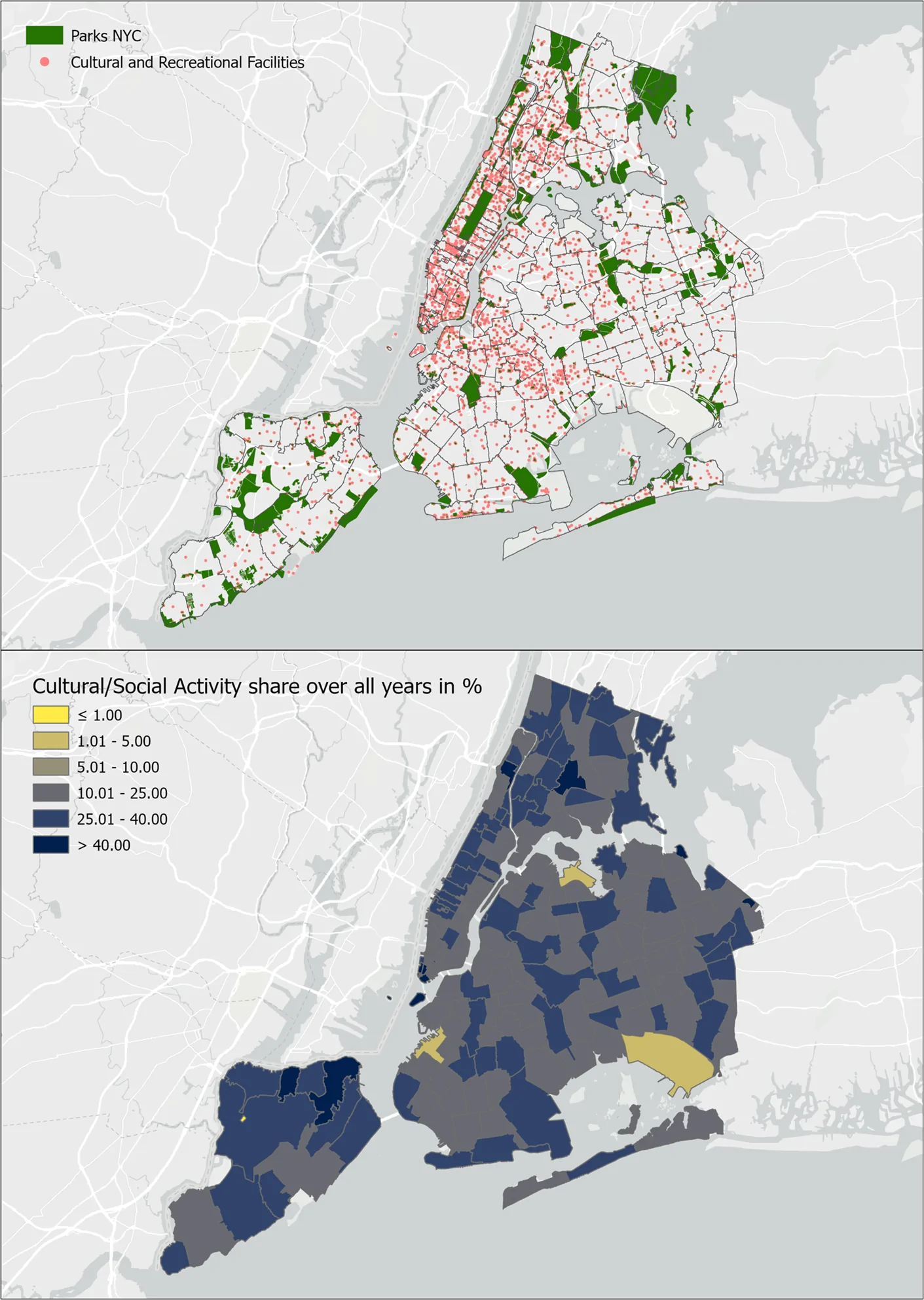
Parks and Cultural-/Recreational Facilities in NYC (NYC Office of Technology & Innovation, 2024a, b) (top) and All-Year Average Share of Cultural/Social Activity-Related Tweets (bottom)

Reflecting the broad nature of cultural and social engagement, Tweet activity in this category was historically prevalent across most ZIP codes, aligning with the citywide distribution of parks and recreational facilities (Fig. 5). The facilities are dispersed across all boroughs but show strong concentrations in Manhattan and dense clusters along major corridors in Brooklyn, Queens, and the Bronx. Staten Island, in contrast, is characterized by a larger share of parkland and more dispersed cultural facilities. In 2018, the corresponding Tweet activity seemed widespread across all boroughs, with high shares in many areas except for a few isolated regions like airports (Fig. 5). However, pre-pandemic deviations remained largely mixed with a few localized intensifications visible in south-central Brooklyn and central Bronx in 2019. Local Moran’s I analysis revealed no clusters of local autocorrelation.

**Fig. 6:**
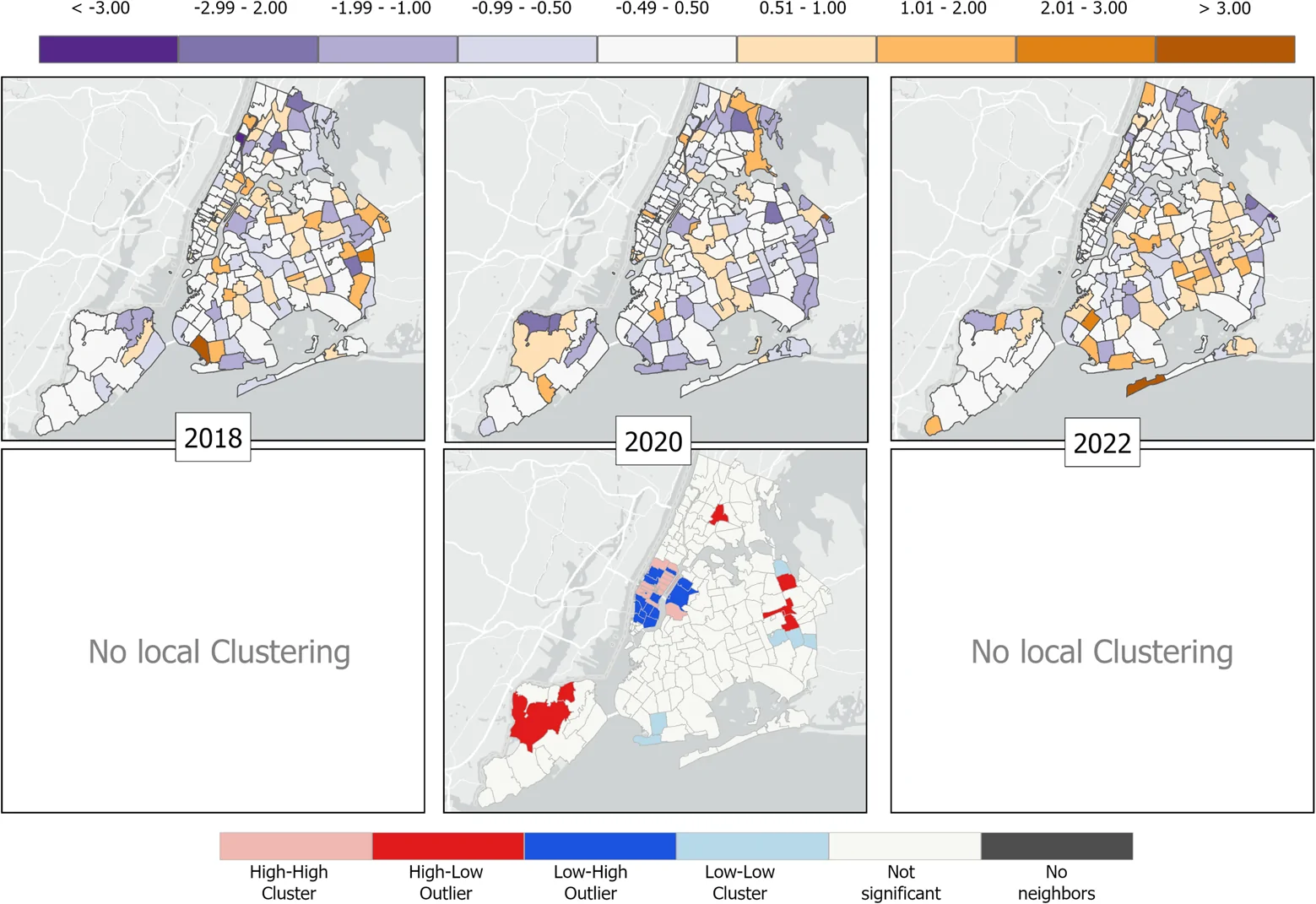
Deviations of Cultural/Social-Activity-Related Tweets from All-Year Average (2018, 2020, 2022) and Local Moran’s I Results

In 2020, the spatial distribution changed more distinctly (Fig. 6). While overall shares remained widespread, an above-average corridor emerged between Brooklyn and Queens, contrasting with surrounding areas where the deviation values dropped below the long-term average. This pattern partly bordered with COVID-19 restriction zones introduced in autumn 2020 (Cuomo 2020a, b, c, d; Harris, 2022). However, this visual corridor also aligned with the higher concentration of recreational facilities in this region (Fig. 5).

Simultaneously, above-average Tweet activity was observed in parts of Staten Island containing larger park areas and the eastern shoreline of the Bronx, coinciding with larger parklands. By 2021 and 2022, activity remained high, but deviations began to shift. Previous above-average activity focused on less concentrated recreational facilities and larger park areas dissolved towards a more mixed pattern again.

The results of Global Moran’s indicated significant clustering for the observed deviations in the years 2019, 2020 and 2022, while 2018 and 2021 did not show statistically significant spatial autocorrelation (Table 4 in Appendix). For the year 2020, Local Moran’s I provided further results (Fig. 6) that partly diverged from the deviation patterns seen there. In Manhattan and parts of northwestern Queens, many ZIP codes were identified as low–high outliers or high-high clusters, even though their deviation values were mostly average, which reflected more their contrast towards adjacent areas rather than strong global deviations. Staten Island, by contrast, showed a clearer alignment, with above-average deviations coinciding with high-low outliers, underlining localized intensifications in the northwest. Around Jamaica in central Queens, a mix of high-low outliers and adjacent low-low clusters pointed to localized contrasts, while Coney Island in south Brooklyn formed a low-low cluster consistent with its below-average deviation.

#### Retail activity

**Fig. 7:**
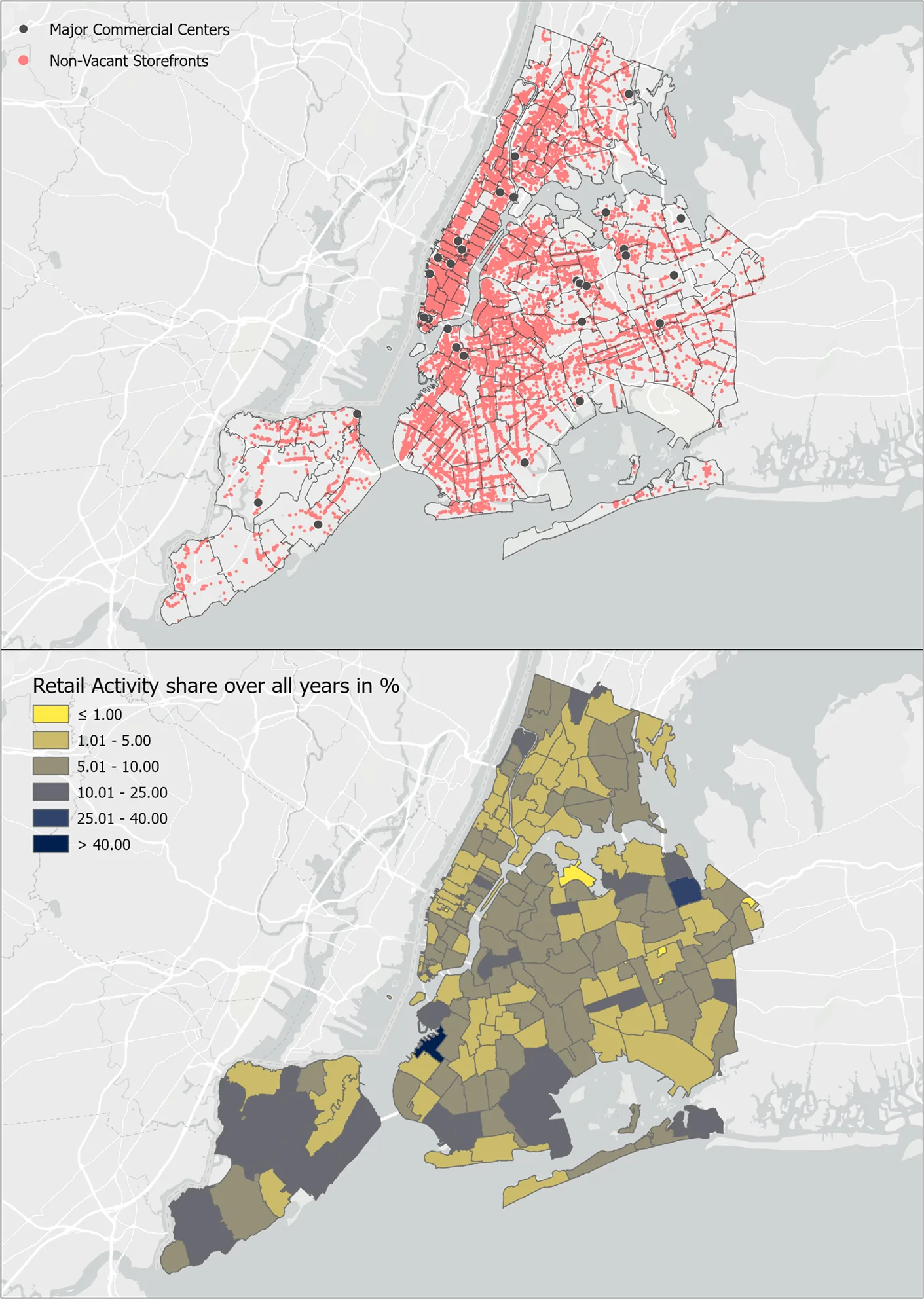
Major Commercial Centers and Non-Vacant Storefronts in NYC (Department of Finance (DOF), 2025) (top) and All-Year Average Share of Retail Activity-Related Tweets (bottom)

In the pre-pandemic period (2018 to 2019), retail-related Tweet shares were spatially dispersed across all boroughs, though 2018 displayed a few isolated high share areas in Brooklyn and Queens while their respective deviations paint a mainly patchy picture with below-average exceptions in Brooklyn and Staten Island (Figs. 28 to 29 in Appendix). By 2019, activity intensified in southern Brooklyn and northeastern Queens, the latter forming a notable above-average area despite containing comparatively few large shopping centers or storefronts (Fig. 7). However, Local Moran’s I analysis revealed that these intensifications were not statistically significant clusters, suggesting that it’s not a cohesive neighborhood pattern. Parts of Staten Island also showed above-average deviations, particularly around commercial areas such as Staten Island Mall in the northwest or Hylan Boulevard in the southeast (Fig. 7).

By the onset of the pandemic in 2020 and continuing through 2021, the spatial distribution remained widespread, yet deviations revealed a more ambivalent pattern. While the underlying share of activity remained broadly distributed (Figs. 30 to 31 in Appendix), the deviation maps showed that above-average values (e.g. in southeastern Brooklyn or central Staten Island) often were interspersed with average or below-average results, preventing the formation of any stable clusters.

**Fig. 8:**
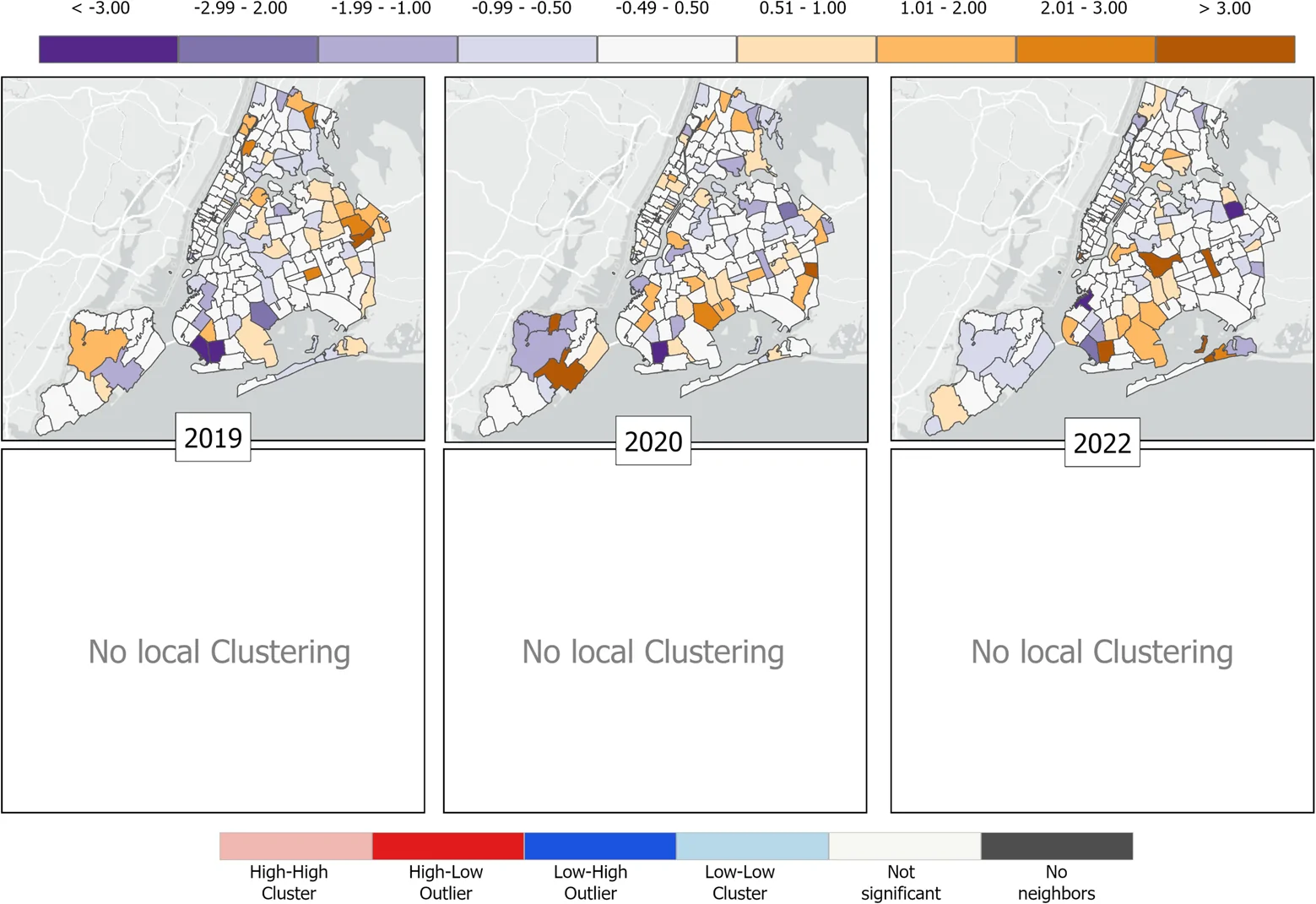
Deviations of Retail Activity-Related Tweets from All-Year Average (2019, 2020, 2022) and Local Moran’s I Results

**Fig. 9:**
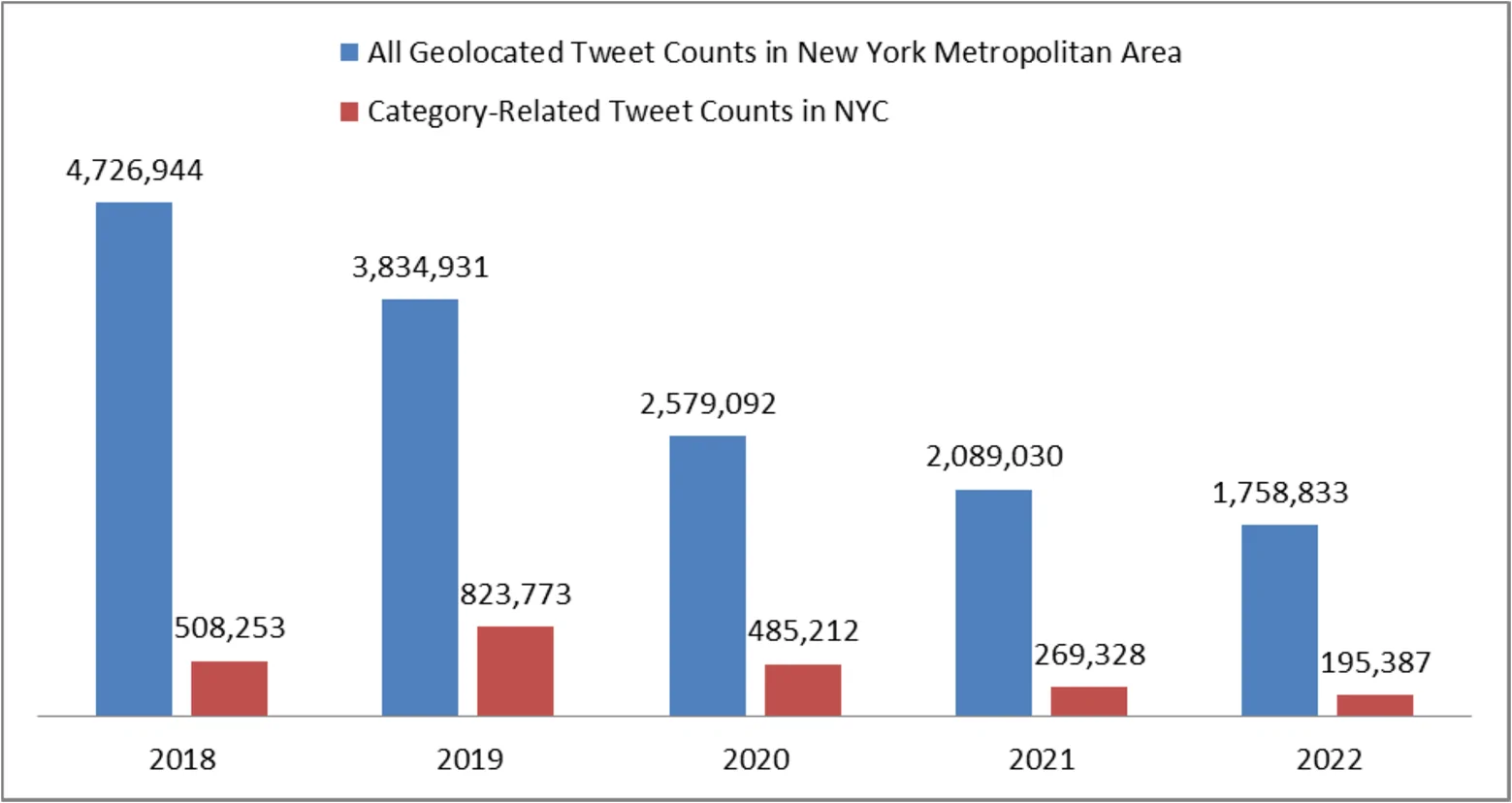
Development of geolocated Tweet Counts per Year: Original Dataset for New York Metropolitan Area (Blue) and final Dataset for New York City (Red)

**Fig. 10:**
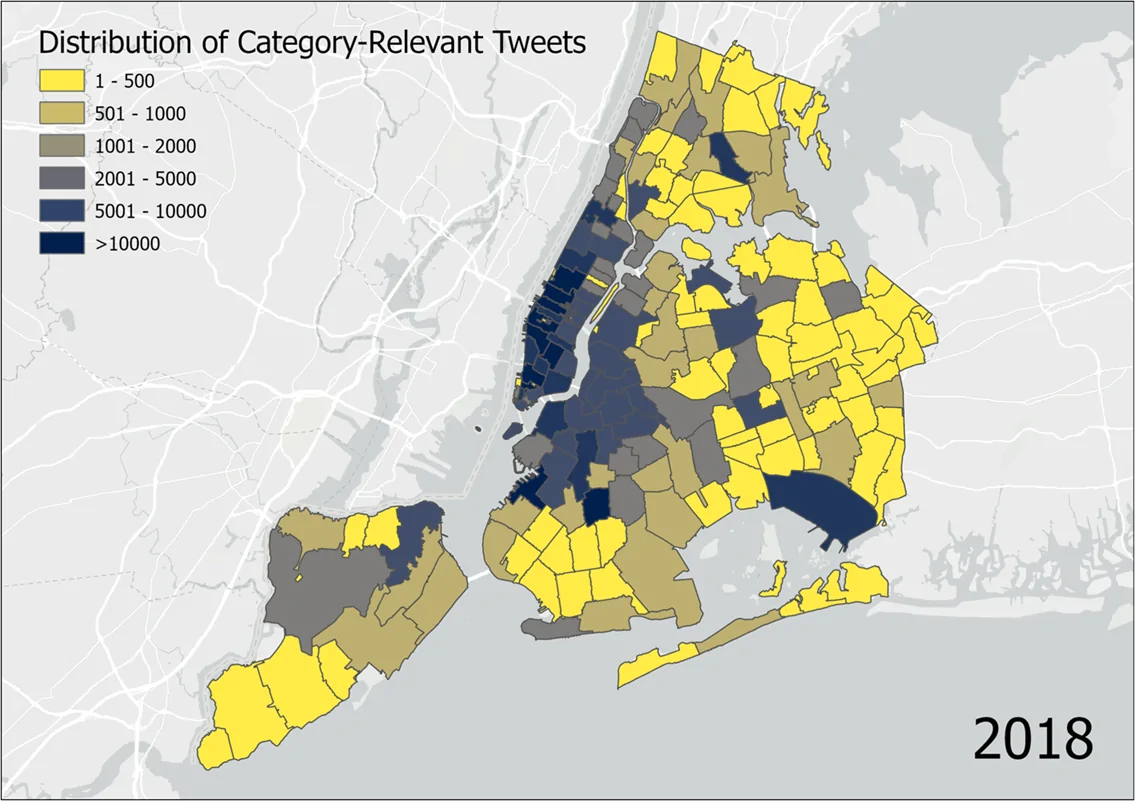
Distribution of Category-Relevant Tweet Counts in NYC 2018

**Fig. 11:**
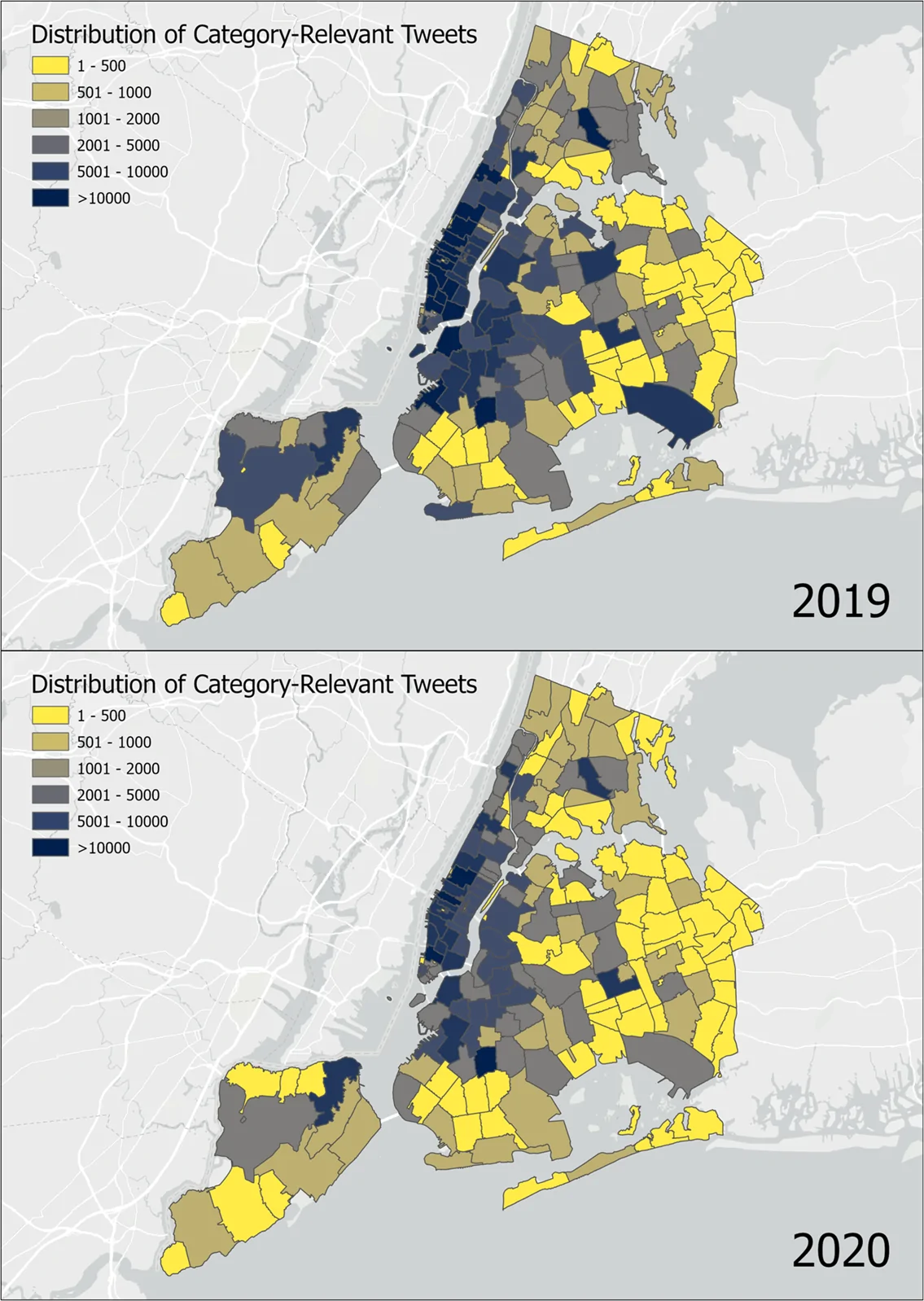
Distribution of Category-Relevant Tweet Counts in NYC 2019 and 2020

**Fig. 12:**
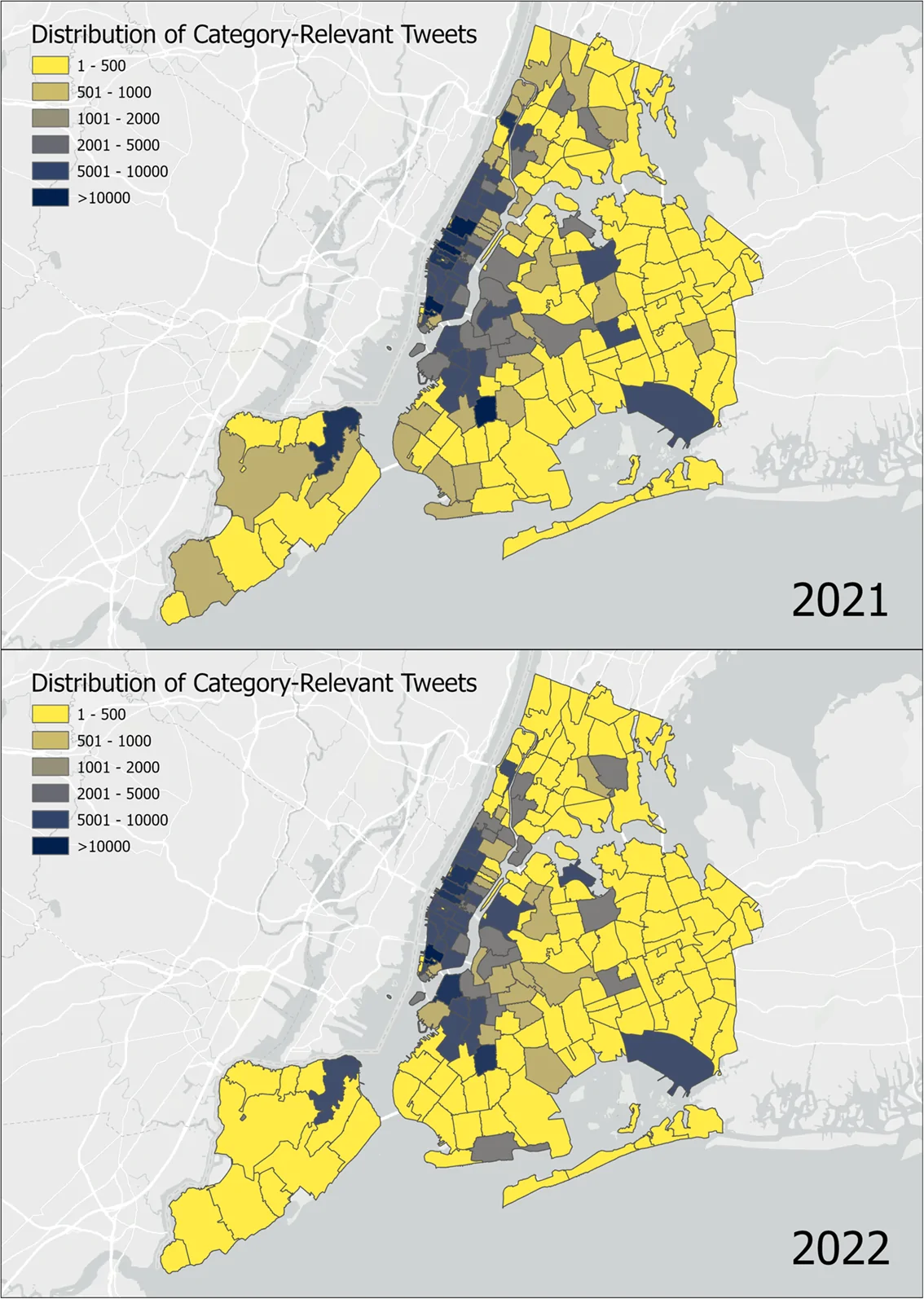
Distribution of Category-Relevant Tweet Counts in NYC 2021 and 2022

**Fig. 28:**
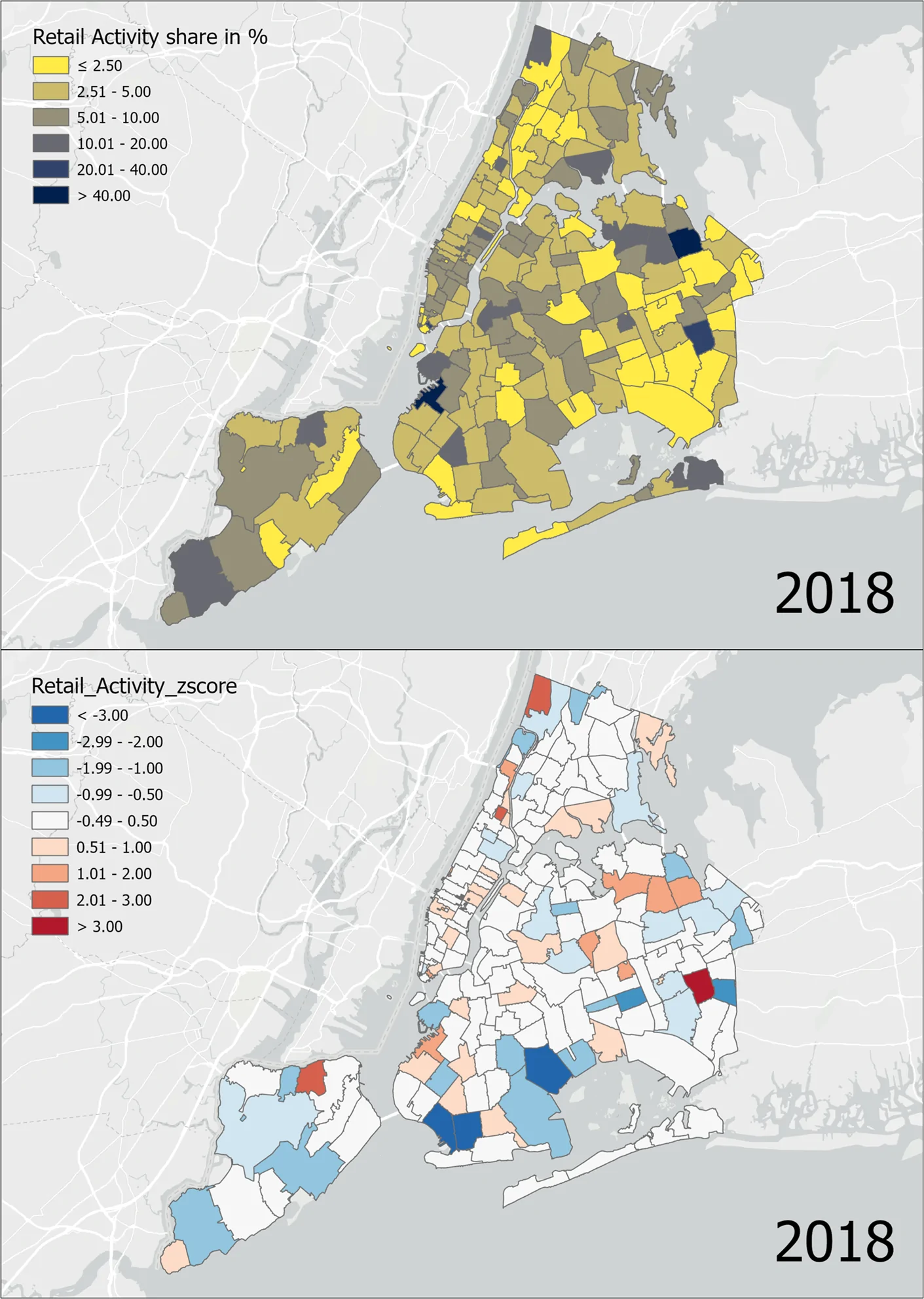
Shares of Retail Activity Category (top) and Deviation of Shares (bottom) from All-Year Average in NYC 2018

**Fig. 29:**
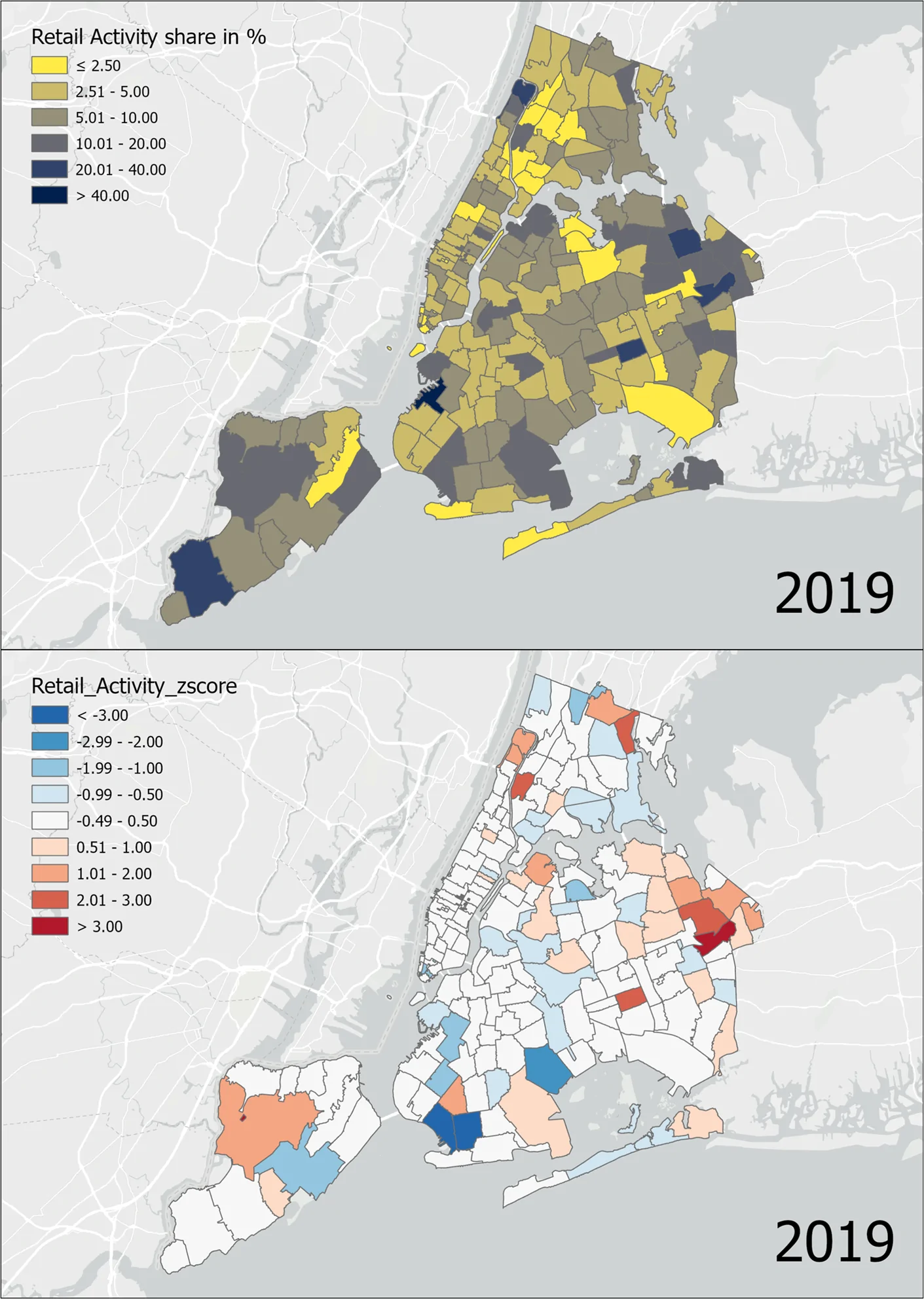
Shares of Retail Activity Category (top) and Deviation of Shares (bottom) from All-Year Average in NYC 2019

**Fig. 30:**
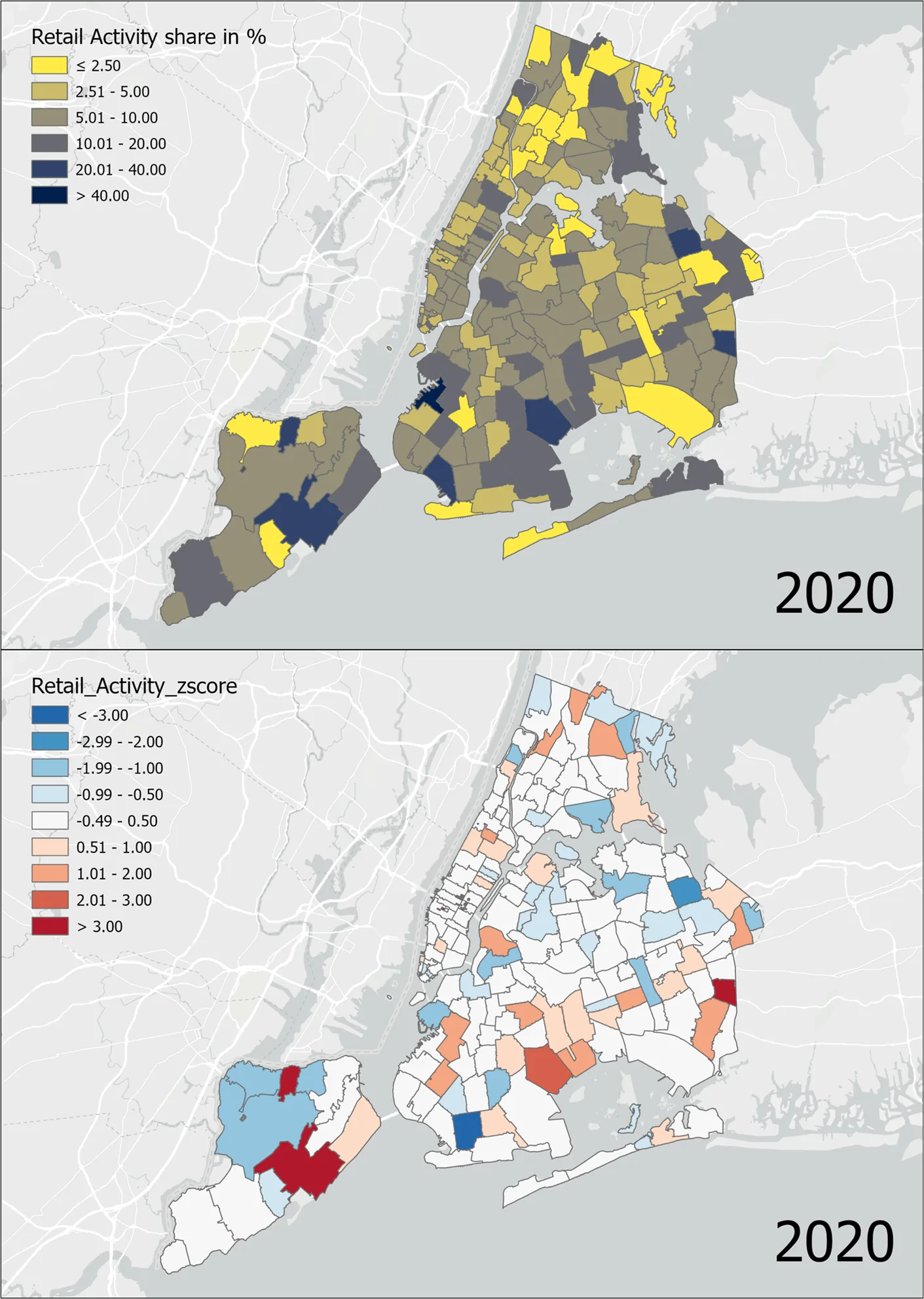
Shares of Retail Activity Category (top) and Deviation of Shares (bottom) from All-Year Average in NYC 2020

**Fig. 31:**
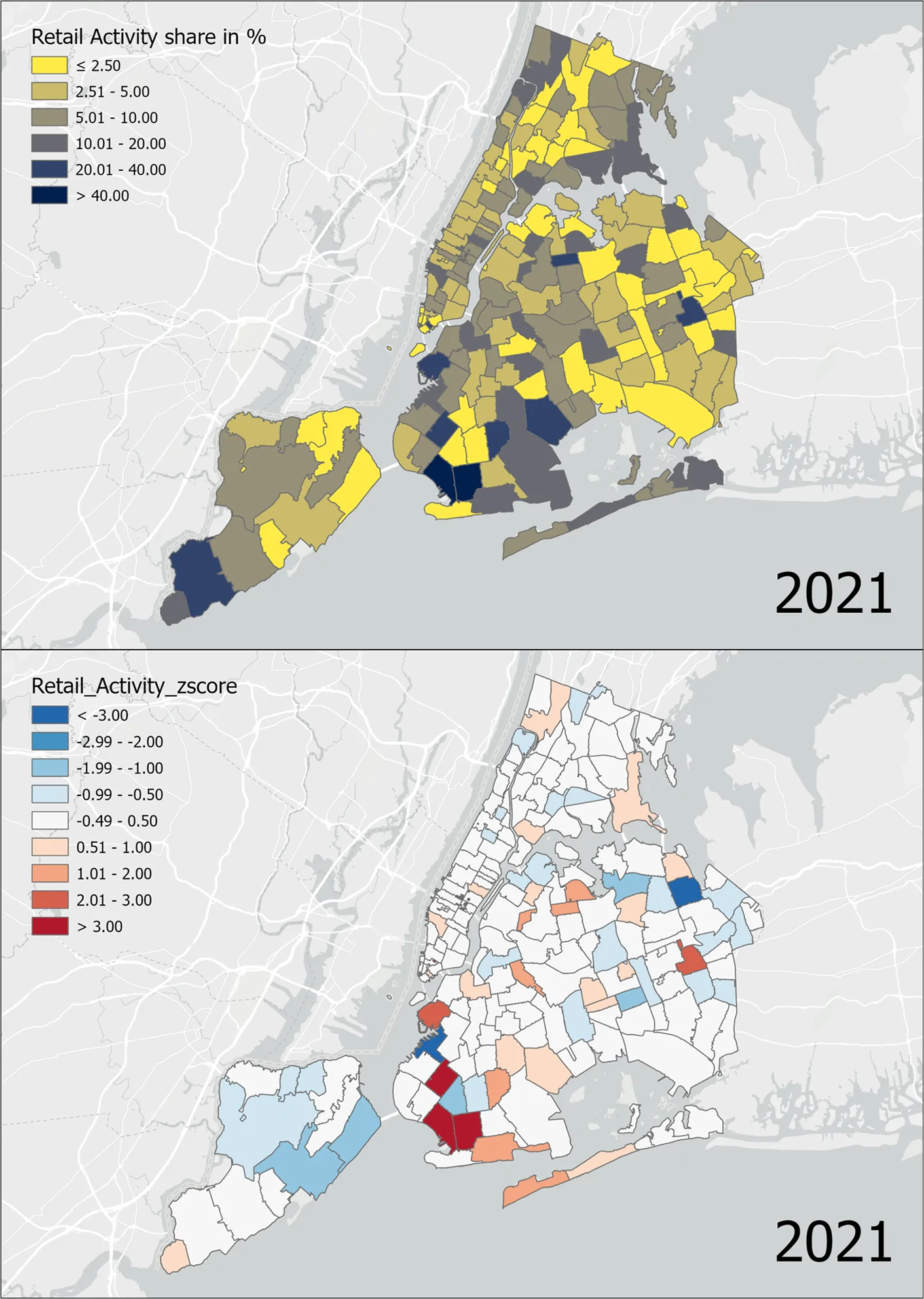
Shares of Retail Activity Category (top) and Deviation of Shares (bottom) from All-Year Average in NYC 2021

**Fig. 32:**
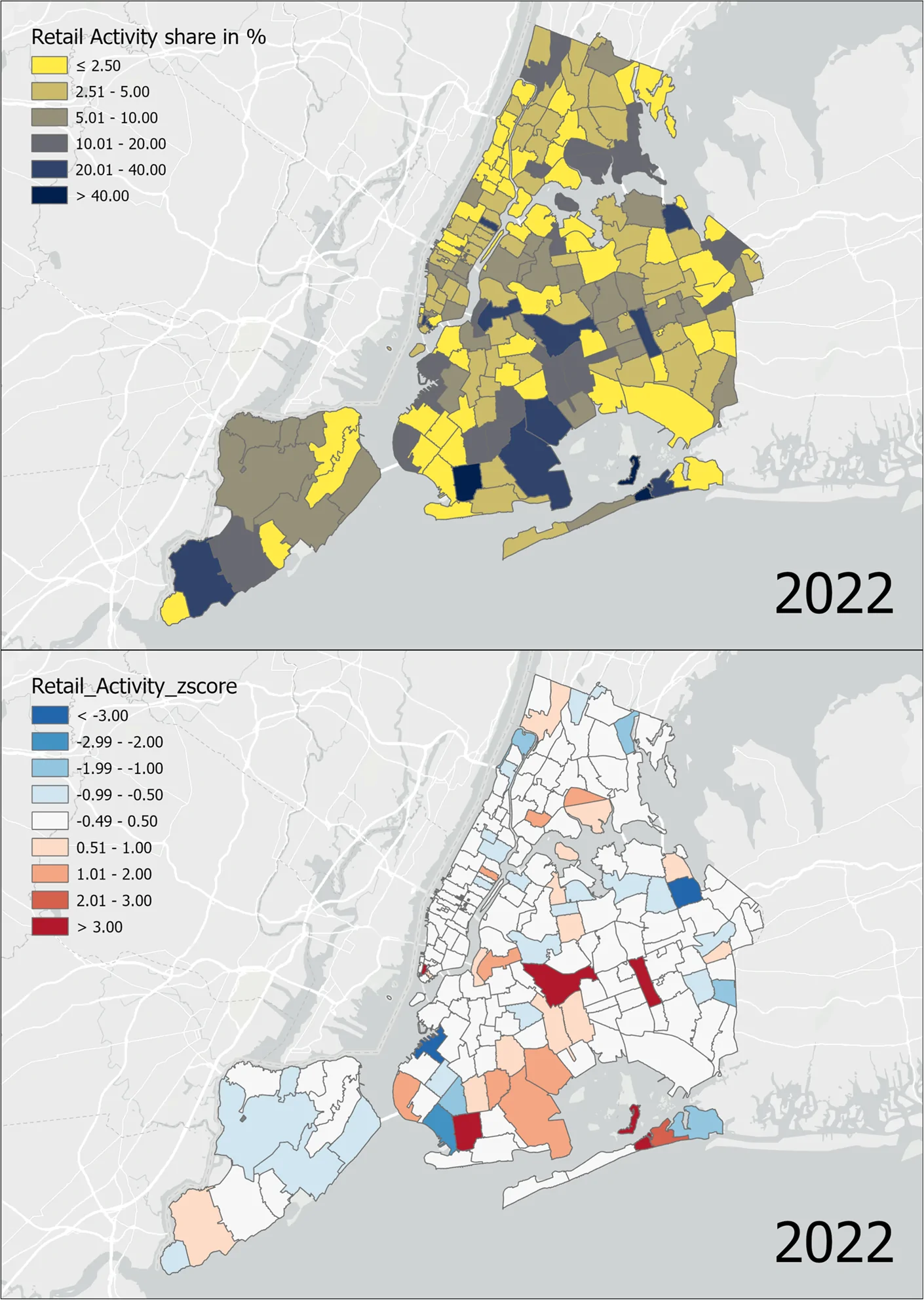
Shares of Retail Activity Category (top) and Deviation of Shares (bottom) from All-Year Average in NYC 2022

By 2022, the spatial structure became slightly more distinct, with high shares grouping in southeastern Brooklyn close to the King’s Plaza and Gateway Center (Fig. 32 in Appendix), aligning with above-average retail-related Tweet activity (Fig. 8). Conversely, boroughs like Manhattan, the Bronx, and Queens displayed mixed patterns with no clear spatial dominance.

Unlike other functional domains, significant spatial autocorrelation was detected only for 2019 (Table 4 in Appendix). However, Local Moran’s I analysis did not reveal any significant local clusters and therefore, the pattern remained limited to broader global tendencies than on a neighborhood level.

## Discussion

### Overview of main findings

Addressing RQ1 and RQ2, the analysis of geolocated Twitter data in New York City between 2018 and 2022 revealed distinct temporal and spatial dynamics across thematic categories. These dynamics provide insights into how urban Tweet activity evolved in response to external disruptions such as the COVID-19 pandemic. However, the interpretability of Tweet patterns differed substantially across categories. Transportation and Cultural/Social Activity showed clearer patterns of contraction, spatial reorganization, and partial recovery, while Retail Activity displayed more fragmented and localized fluctuations. Healthcare and Work/Remote Work, by contrast, produced only limited and spatially patchy signals, suggesting that their geotagged Tweet volumes were too sparse to support robust spatial and temporal interpretation.

This thematic imbalance is consistent with previous research showing that leisure and socially oriented activities are often overrepresented in social media data, while still providing useful signals for quantifying mobility and urban activity patterns (Jurdak et al., 2015; Liao et al., 2022). Taken together, the findings suggest that geotagged Tweets do not provide a comprehensive representation of urban behavior. Rather, they capture selective digital traces of urban activity, shaped by platform use, user demographics, thematic engagement, and the uneven spatial distribution of geotagged content.

### Thematic interpretation of spatial and temporal patterns

#### Transportation activity

Transportation-related Tweet activity showed one of the clearest spatial and temporal developments across the study period. Before the pandemic, transportation-related Tweet intensity was spatially scattered, with even Manhattan showing below-average activity. During the pandemic year, overall activity decreased, while comparatively higher Tweet shares appeared around selected transportation hubs, such as Jamaica Station. Similar contractions in general and transportation-related mobility during the early pandemic phase were observed by Rajput et al. (2022) and Jiang et al. (2021), who reported substantial declines following city-wide restrictions in New York City.

By 2022, a highly significant clustering pattern emerged, with high-high clusters along subway and railway lines in Manhattan and Brooklyn. This may indicate a renewed concentration of transportation-related online discourse in areas associated with transit infrastructure. While this interpretation requires further validation, it aligns with studies describing post-pandemic recovery dynamics in mobility-related data near transit infrastructure in cities such as London and Toronto (Forouhar et al., 2025; Zhong et al., 2023). For New York City, Qiang and McKenzie (2024) similarly emphasize that the return of public transport activity varied across pandemic phases and was shaped by anti-pandemic policies and socio-economic characteristics.

These findings suggest that geo-social media data may capture transportation-related shifts in online attention and discourse within urban environments. However, the observed spatial correspondence should not be interpreted as direct evidence of actual changes in public transportation usage. Instead, it indicates that transportation infrastructure became more visible in geotagged Twitter discourse during the post-pandemic recovery phase. Future work should therefore examine to what degree changes in transportation-related social media activity can be statistically linked to actual mobility indicators, such as passenger counts, station-level transit usage, or mobile-phone-derived mobility flows.

#### Cultural and social activity

The Cultural/Social Activity category displayed a broad spatial footprint before the pandemic, underscoring the central role of cultural and social activities in urban life (Liao et al., 2022). In 2020, during pandemic restrictions, the spatial pattern became more uneven. A pronounced above-average corridor appeared between Brooklyn and Queens, partly bordering areas affected by COVID-19 restriction zones and areas containing dense concentrations of cultural venues and facilities. Although previous research has examined the limited effectiveness of these zones in containing infections (Harris, 2022), cultural and social activity patterns in these areas have received less attention. The spatial correspondence observed here may therefore suggest that localized engagement with cultural and social themes persisted in this corridor despite broader disruptions to urban activity.

At the same time, parts of Staten Island and the eastern shoreline of the Bronx showed above-average cultural/social Tweet intensity during 2020, particularly in areas containing larger parklands. This may reflect increased online engagement with outdoor-related cultural and social themes during periods of restriction. This interpretation is consistent with Zhao et al. (2023), who found that nature areas in New York City experienced stable or increased visitation during the pandemic and served as perceived safe havens for residents, especially in outer-borough areas. Their findings suggest that recreational behavior shifted toward peripheral parks as residents adapted to pandemic-related restrictions.

For cultural and social activities, statistically significant local clusters were mainly observed in Staten Island. These clusters likely reflect the borough’s large parklands and lower-density urban structure, which provided accessible outdoor venues for social and cultural activity during restrictions (Zhao et al., 2023). In contrast, the visually apparent Brooklyn-Queens corridor and the eastern Bronx shoreline did not show statistically significant clustering. This distinction highlights the importance of separating visually observable spatial patterns from statistically supported local clusters.

Following the pandemic-related clustering of deviations, the post-pandemic years showed a gradual return to more mixed spatial patterns. This aligns with previous observations that cultural and creative social media activity is often less spatially concentrated and more widely distributed across urban landscapes (Niu & Silva, 2023; Reuschke et al., 2021). Such patterns reflect the decentralized and diverse nature of cultural engagement in large cities.

#### Retail activity

Retail Activity Tweets exhibited more fragmented and less spatially coherent patterns than Transportation or Cultural/Social Activity. Although 2019 showed globally significant clustering of retail-related Tweet activity, the Local Moran’s I analysis did not identify coherent local clusters in the visually assessed above-average region in northeastern Queens. Similarly, localized increases in southern Brooklyn in 2022 near commercial centers such as King’s Plaza were not supported by statistically significant local clustering and should therefore be interpreted as visual tendencies rather than robust spatial clusters.

This heterogeneity may reflect the diverse nature of retail-related social media interactions. Retail Tweets can include consumer reviews, business promotions, complaints, advertisements, or general references to shopping locations, each of which may follow different spatial and temporal dynamics. Li et al. (2023) demonstrate that customers engage in different forms of social media interaction during crises, with each type of interaction reflecting different attributes and motivations. These varying forms of engagement likely contributed to the fragmented spatial structure of retail-related Tweet activity observed in this study.

Consequently, the retail category illustrates both the potential and the limitations of thematic geo-social media analysis. While retail-related Tweets may reveal localized signals of commercial activity, their interpretation requires a more detailed distinction between consumer-generated and business-generated content. Future research could therefore separate promotional, transactional, experiential, and complaint-based retail Tweets to better understand which types of retail-related discourse are most informative for urban activity analysis.

#### Sparse categories: healthcare and work/remote work

Healthcare and Work/Remote Work produced comparatively weak and spatially fragmented signals. Although both categories were conceptually relevant during the COVID-19 pandemic, the volume and spatial density of geotagged Tweets in these categories were too limited to support robust interpretation. This finding is important because it demonstrates that thematic relevance alone does not guarantee analytical usefulness in geo-social media data. A category may be socially or politically important, but still remain underrepresented in geotagged Twitter activity.

The limited signal in these categories may be linked to several factors. Healthcare-related discussions may occur without geotags, may be expressed in more general pandemic-related language, or may not directly mention healthcare facilities and services. Similarly, Work/Remote Work activity may be difficult to identify spatially because remote work is often detached from specific urban locations. As a result, these categories should be treated as exploratory rather than conclusive in the present study.

### Methodological contribution

The methodological framework combined keyword filtering with BERTopic-based topic clustering to classify geotagged Tweets into urban functional categories. This approach achieved precision scores above 90%, suggesting that the combination of keyword filtering and BERTopic-based classification can provide a relatively robust framework for capturing urban functional categories in geotagged Twitter data.

Beyond the achieved precision values, the workflow also demonstrated methodological transferability. Since the keyword generation process was based on English-language Wikipedia corpora rather than city-specific training data, large parts of the resulting keyword sets were reusable across different urban contexts. The workflow and keyword sets were initially developed and tested using translated data from Vienna and Berlin and were later transferred to New York City with only limited adaptation through the addition of local points of interest, landmarks, and institutions. This suggests that the proposed framework may provide a comparatively transferable approach for thematic classification of urban geo-social media data across different cities, provided that Tweets are available in English or translated accordingly.

The methodological design also contributed to the interpretation of pandemic-related urban change. Analyzing deviations from long-term baseline category shares proved useful for detecting spatially clustered disruptions, especially in thematic domains such as Transportation and Cultural/Social Activity. In this year-wise configuration, the approach functions primarily as a retrospective analytical tool. It highlights where persistent deviations in categorical activity emerged and where digital traces of urban disruption became spatially visible. If applied to datasets with higher temporal resolution, the same methodology could potentially be extended toward more dynamic monitoring of urban activity patterns.

### Limitations

Several limitations need to be considered when interpreting the results. First, the study relies on geolocated Tweets, which represent only a fraction of all Twitter activity. As a result, the dataset does not capture citywide communication in its entirety. Instead, it reflects the activity of a digitally active minority that chooses or happens to produce geotagged content. This limitation is widely acknowledged in geo-social media research, where user demographics, platform-specific behavior, and uneven participation can bias the interpretation of spatial patterns (De Sabbata et al., 2023; Almatar et al., 2020; Kovacs-Györi et al., 2018; Yang & Liu, 2022).

Second, normalized category shares at the ZIP code level may be sensitive to low underlying Tweet counts. In areas or categories with sparse Tweet activity, small absolute differences can translate into comparatively large relative deviations. Therefore, spatial patterns in low-volume areas should be interpreted cautiously and primarily as exploratory indicators rather than stable estimates of urban activity.

Third, the classification workflow includes interpretative and manual components. The reported precision partly reflects the manual mapping of BERTopic-generated topics to higher-level categories, while the preprocessing workflow was also optimized toward this metric. In addition, the keyword extraction process required iterative parameter tuning to maximize topic relevance and minimize noisy terms. Similar challenges have been noted by De Sabbata et al. (2023) and G. Almatar et al. (2020), who emphasize that topic identification in social media data often requires manual refinement to preserve conceptual coherence.

Fourth, the hybrid workflow applies BERTopic only after keyword filtering, meaning that only Tweets with category-related keywords entered the topic model. This improved precision but contributed to lower overall category recall, as relevant Tweets could be missed if they lacked explicit keywords or could not be assigned to coherent BERTopic clusters. The large share of “−1” outlier topics reflects this limitation and aligns with De Sabbata et al. (2023) and Steiger et al. (2016), who show that sparse or heterogeneous Twitter data can remain unclustered. The recall evaluation therefore indicates a selection bias toward Tweets with explicit terminology, while indirect, colloquial, or heterogeneous expressions may be underrepresented. However, this reflects a deliberate methodological choice to prioritize precision and interpretability of the final topics over higher recall. Consequently, the category distributions should be read as conservative indicators of clearly identifiable category-related Tweet activity rather than complete representations of urban activity. Moreover, since the five categories do not cover the full thematic diversity of urban Twitter activity, uncategorized Tweets may also contain meaningful topics outside the analytical framework.

Fifth, linguistic coverage remains limited. Although BERTopic can support multilingual input (Egger & Yu, 2022), the hybrid nature of the workflow would require additional steps, such as translating Tweets and keywords into the target language or curating equivalent keyword lists for each language. Jiang et al. (2023) note that extending BERTopic to additional languages can also create computational challenges when working with large datasets. This limitation is particularly relevant for a city such as New York, where multilingual communication is common. The Cultural/Social Activity category, in particular, may benefit from multilingual analysis because it could better capture posts by tourists and multilingual residents.

Sixth, the detection of non-individual accounts remains imperfect. The heuristic approach used in this study was effective in removing obvious organizational accounts, but it may have excluded some individual users or retained more subtle automated, semi-automated, or organizational accounts. Ferrara et al. (2016) show that social bots can distort online activity by amplifying noise and making it more difficult to distinguish human from automated behavior. Residual automated or organizational users may therefore have influenced some of the observed spatial and thematic patterns.

Finally, the validation strategy relied primarily on spatial correspondence with urban facilities and infrastructure rather than direct comparison with official statistical indicators. For example, transport-related patterns were compared with public transport lines and hubs, while retail-related patterns were compared with commercial center locations. This approach ensured that the interpretation remained anchored in the urban landscape and is comparable to previous studies that validated Tweet-derived activity patterns against land use data, landmark locations, and transport hubs (Frias-Martinez et al., 2012; Lansley & Longley, 2016; Steiger et al., 2016). However, spatial correspondence alone cannot establish causality or confirm that Tweet activity directly reflects actual behavioral change.

### Future research

Future research should extend the present framework in several directions. First, the relationship between geo-social media activity and official urban activity indicators should be tested more systematically. Transportation-related Tweet patterns could be compared with station-level passenger counts or mobility data. Retail-related Tweet activity could be linked to store openings and closures, retail revenue, footfall data, or business registry information. Such comparisons would help determine whether geotagged Twitter data can serve as a reliable proxy for specific forms of urban activity.

Second, future studies should improve the spatial representativeness of the data. One option would be to infer locations from Tweet text through explicit place-name mentions, as demonstrated by Serere et al. (2023). Another option would be to combine geotagged Tweets with self-reported user location information, as suggested by G. Almatar et al. (2020). These extensions could substantially increase spatial coverage and reduce dependence on explicitly geotagged Tweets.

Third, future work should explore multilingual extensions of the workflow. This would be particularly useful for culturally diverse urban environments, where English-only data may miss important parts of social media activity. Multilingual analysis could also help assess whether categories such as Cultural/Social Activity capture different patterns when tourist activity and multilingual resident communication are included.

Fourth, the classification workflow could be further automated and tested against alternative topic modeling approaches. Future research could compare different BERTopic variants, embedding models, clustering parameters, and large-language-model-assisted classification strategies. Such comparisons would help determine how sensitive the observed spatial patterns are to modeling decisions and how reproducible the workflow is across cities and time periods.

Fifth, future studies could improve the treatment of bots, organizational accounts, and business-generated content. Instead of removing organizational accounts entirely, future research could treat them as a separate analytical category, as suggested by McCorriston et al. (2015). This would be especially relevant for retail activity, where business-generated posts may carry different meanings than consumer-generated posts.

Finally, future research could apply the methodology at higher temporal resolution. While the present study uses a year-wise configuration, weekly or monthly analysis could reveal more precise temporal responses to policy changes, infection waves, seasonal effects, or major urban events. Such an extension would allow the framework to move from retrospective analysis toward a more dynamic monitoring approach for urban activity disruptions and recovery processes.

## Conclusion

This study explored how geotagged Twitter data can be used to analyze thematic Tweet activity related to urban functional categories in New York City between 2018 and 2022. The developed workflow shows that Wikipedia-derived keyword filtering, BERTopic-based classification, baseline normalization, and spatial autocorrelation analysis can be combined to structure large-scale Twitter data into interpretable urban categories and examine thematic changes over time and space.

Overall, the results demonstrate that geotagged Twitter data can provide meaningful but selective insights into urban activity dynamics. The strongest signals appeared in categories with sustained online engagement and clear spatial anchoring, particularly Transportation and Cultural/Social Activity. Transportation provided the clearest post-pandemic recovery signal, with significant spatial clustering emerging in 2022 along subway and railway corridors in Manhattan and Brooklyn. Cultural/Social Activity also displayed meaningful spatial variation, particularly during 2020. In contrast, Retail Activity showed more fragmented patterns, while Healthcare and Work/Remote Work remained too sparse for robust spatial interpretation.

The study therefore contributes both empirically and methodologically. Empirically, it shows how pandemic-related disruptions and recovery processes became visible in selected forms of urban Twitter activity across New York City. Methodologically, it demonstrates how keyword filtering, topic modeling, baseline normalization, and spatial autocorrelation analysis can be combined to examine thematic changes in geotagged social media data. At the same time, the findings should be understood as exploratory indicators of digital urban activity rather than direct measurements of citywide behavior.

These differences underline that the analytical value of geo-social media data depends strongly on thematic context, data density, and the relationship between online discourse and offline urban activity. Used carefully, such data can complement official statistics by highlighting localized disruptions, persistent voids, and emerging patterns of urban attention that may otherwise remain hidden in aggregate citywide indicators.

Future work could expand this approach across multiple cities, incorporate multilingual and inferred location data, and compare Tweet-derived patterns with external indicators such as transit ridership, retail activity, or park visitation data. Such extensions would help further assess when and where geotagged social media data can provide reliable insights into urban disruptions and recovery processes.

## Figures and Tables

**Fig. 13:**
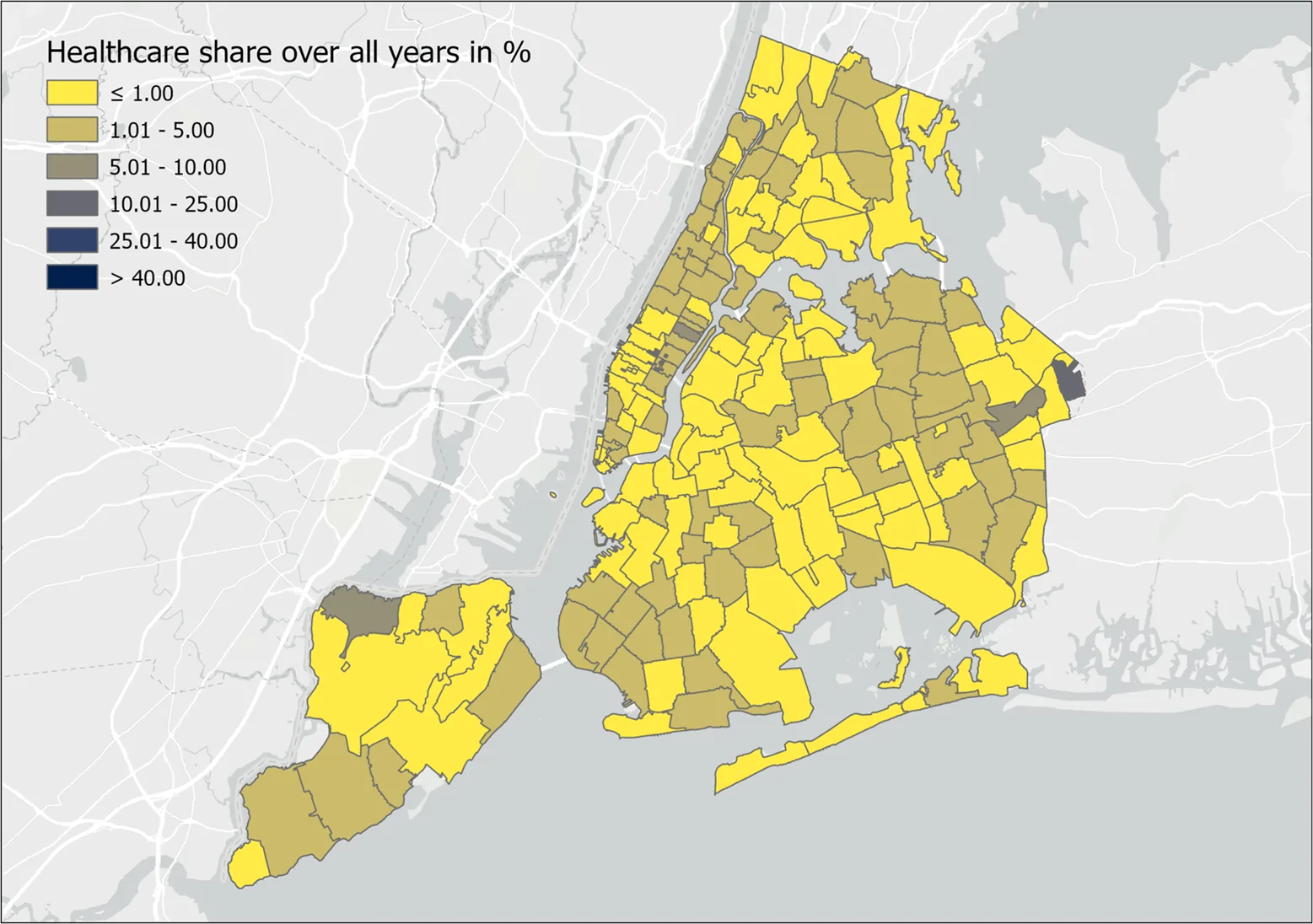
All-Year Average Shares Healthcare in NYC

**Fig. 14:**
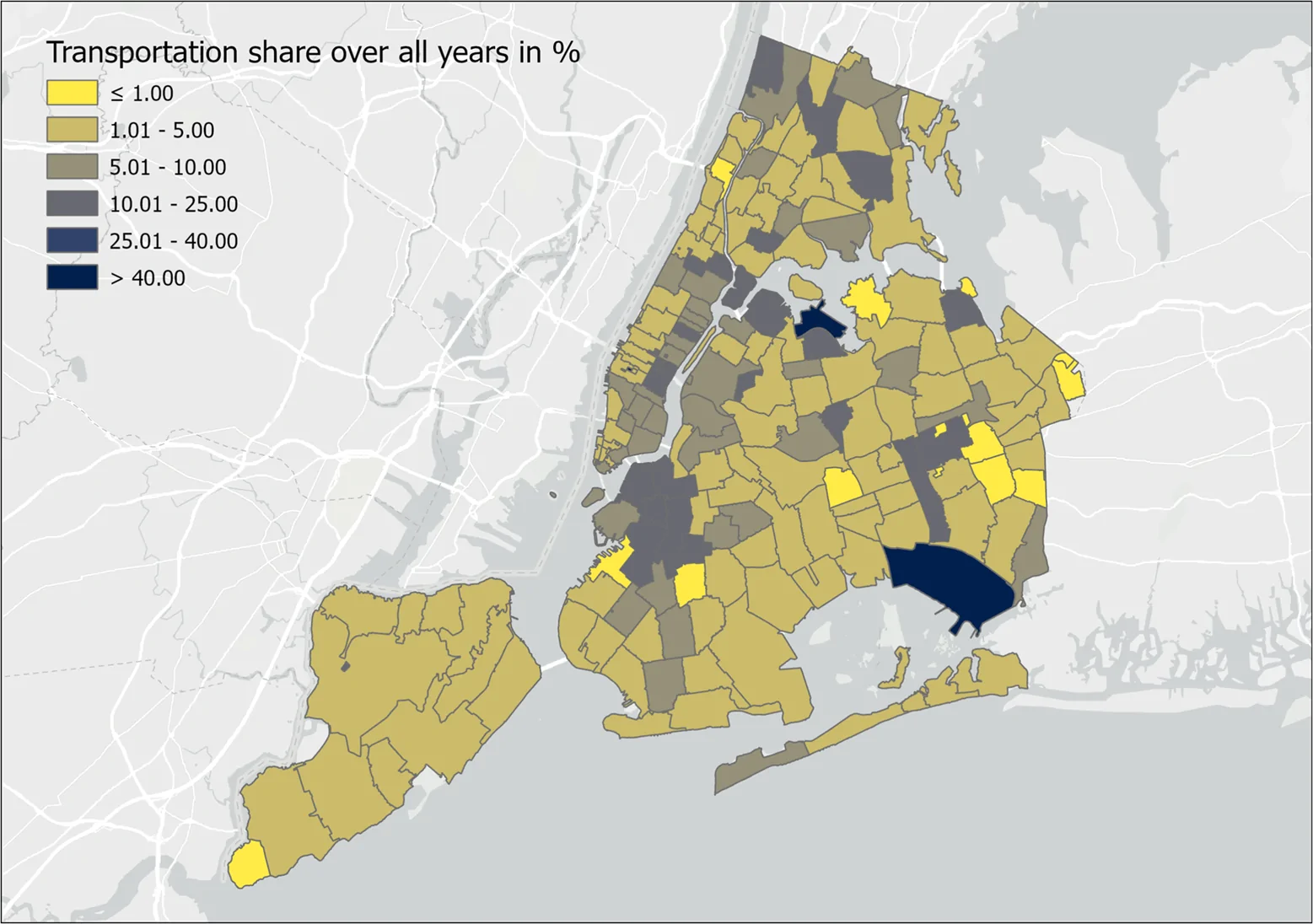
All-Year Average Shares Transportation in NYC

**Fig. 15:**
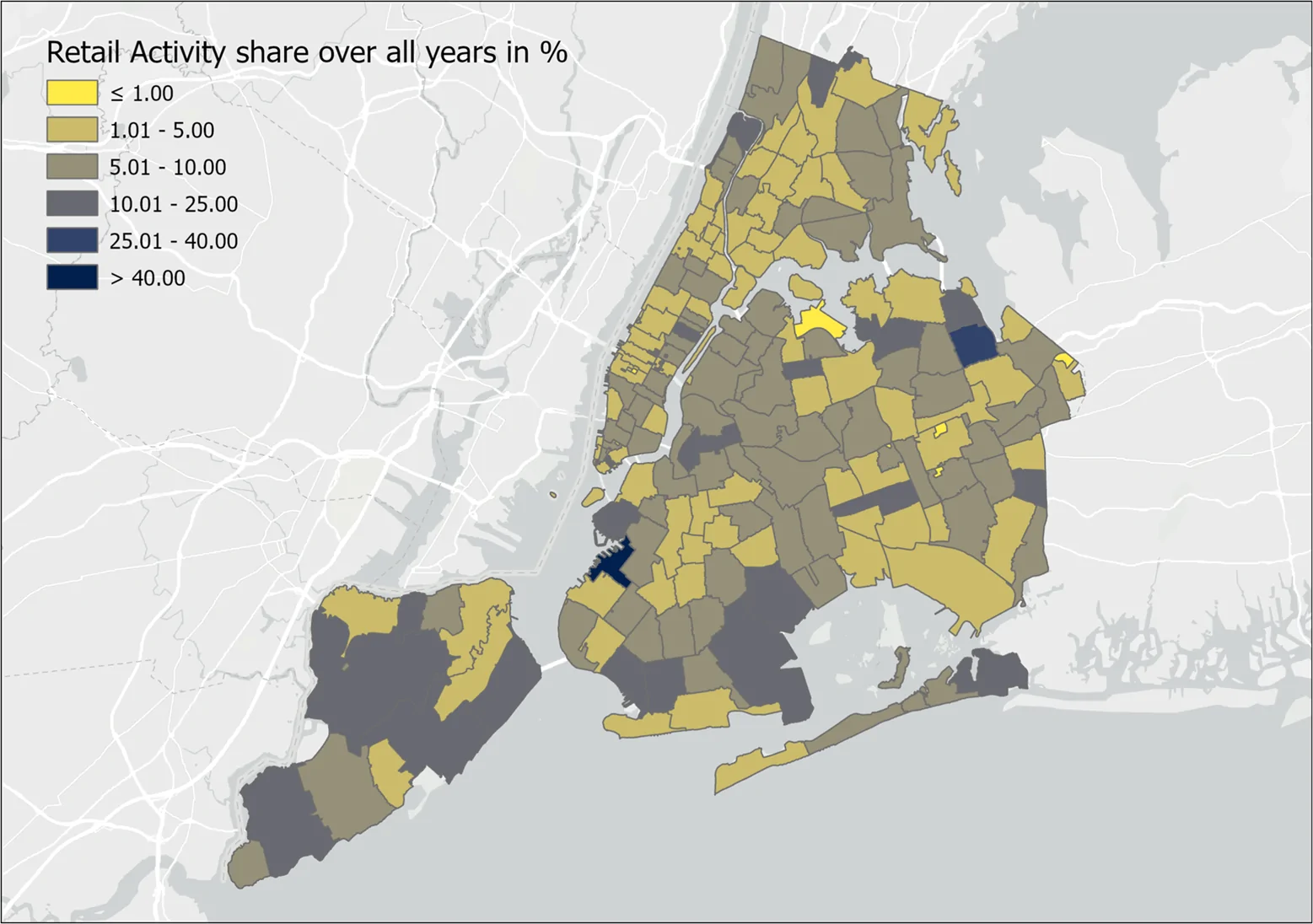
All-Year Average Shares Retail Activity in NYC

**Fig. 16:**
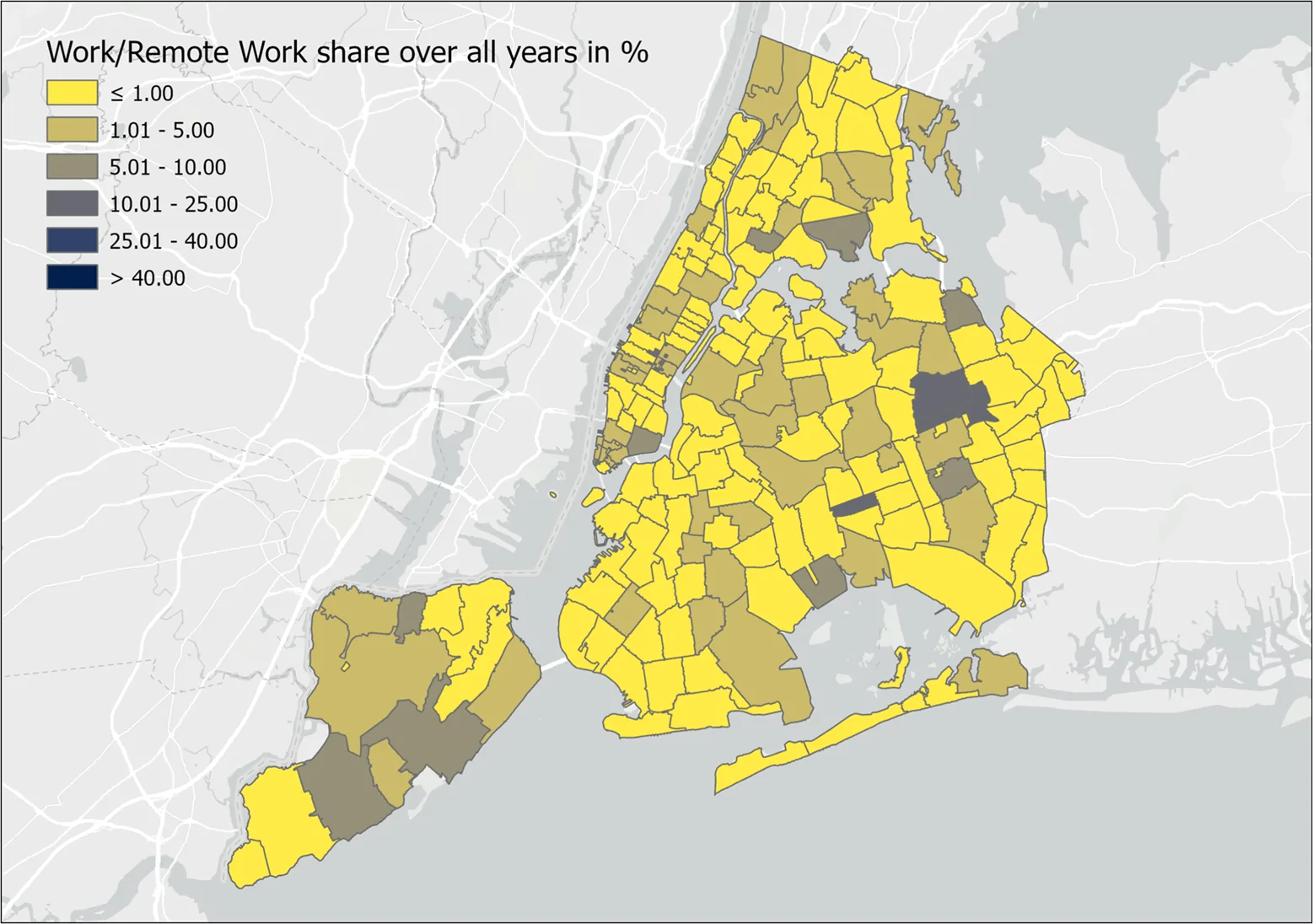
All-Year Average Shares Work/Remote Work in NYC

**Fig. 17:**
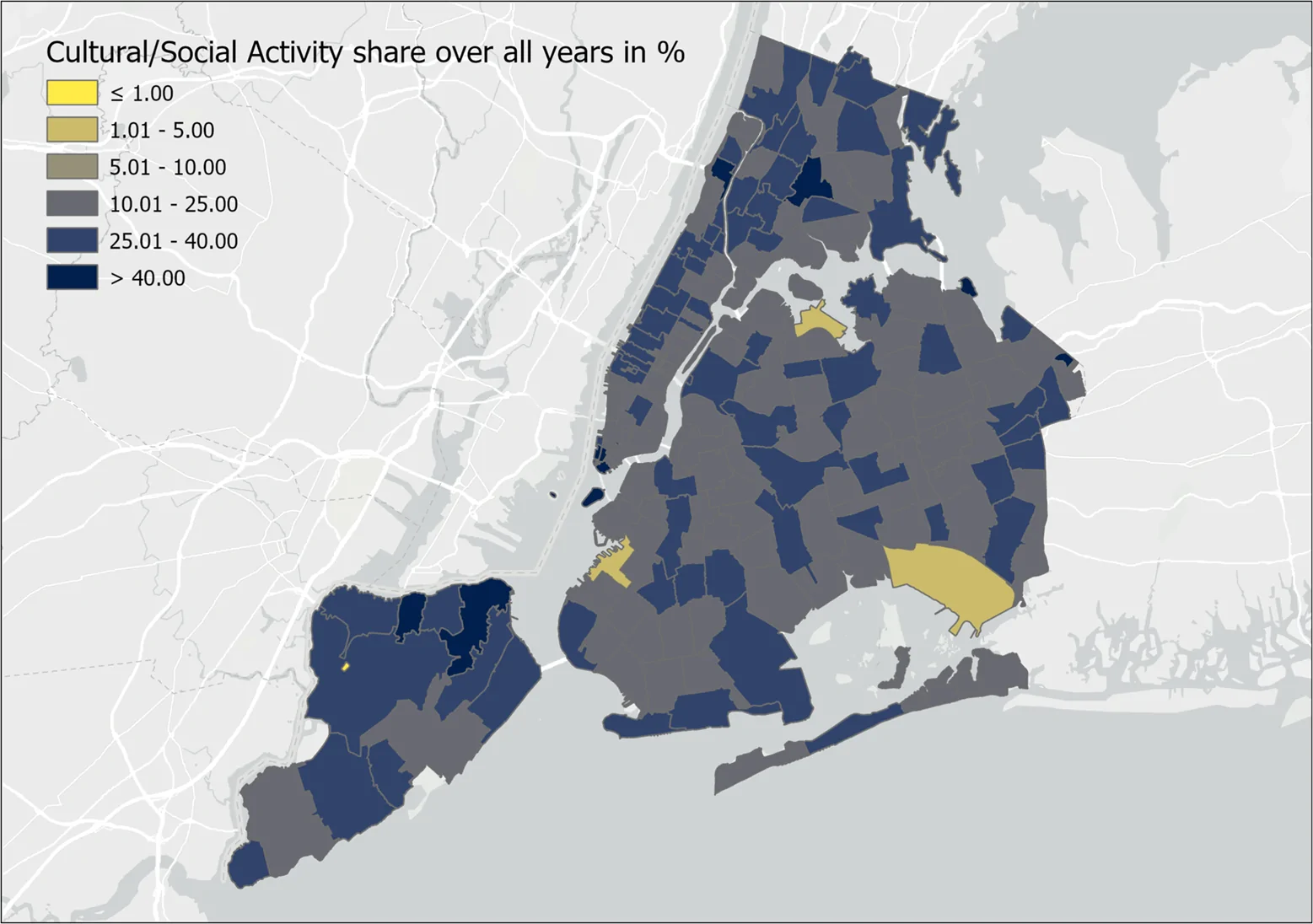
All-Year Average Shares Cultural/Social Activity in NYC

**Fig. 18:**
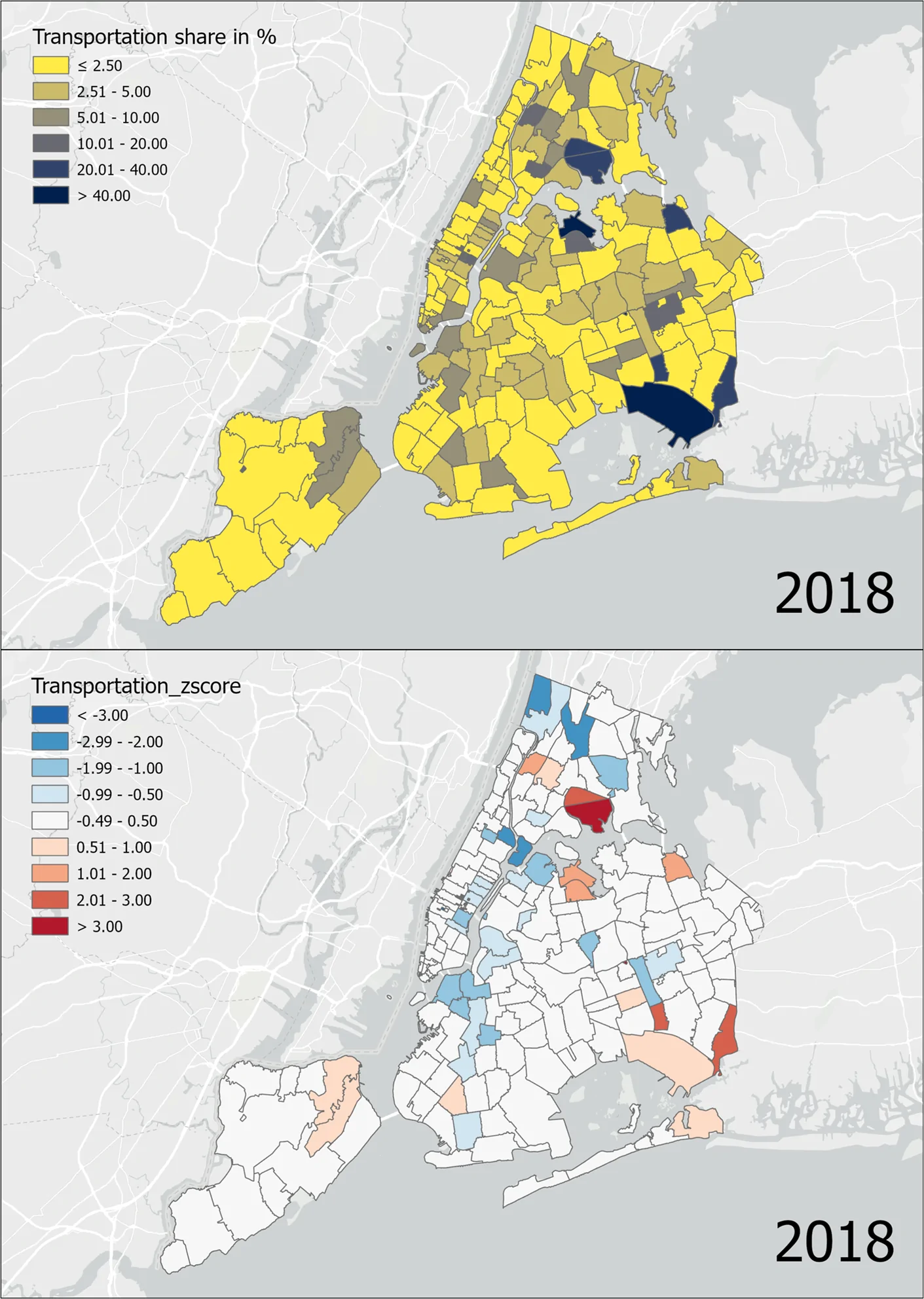
Shares of Transportation Category (top) and Deviation of Shares (bottom) from All-Year Average in NYC 2018

**Fig. 19:**
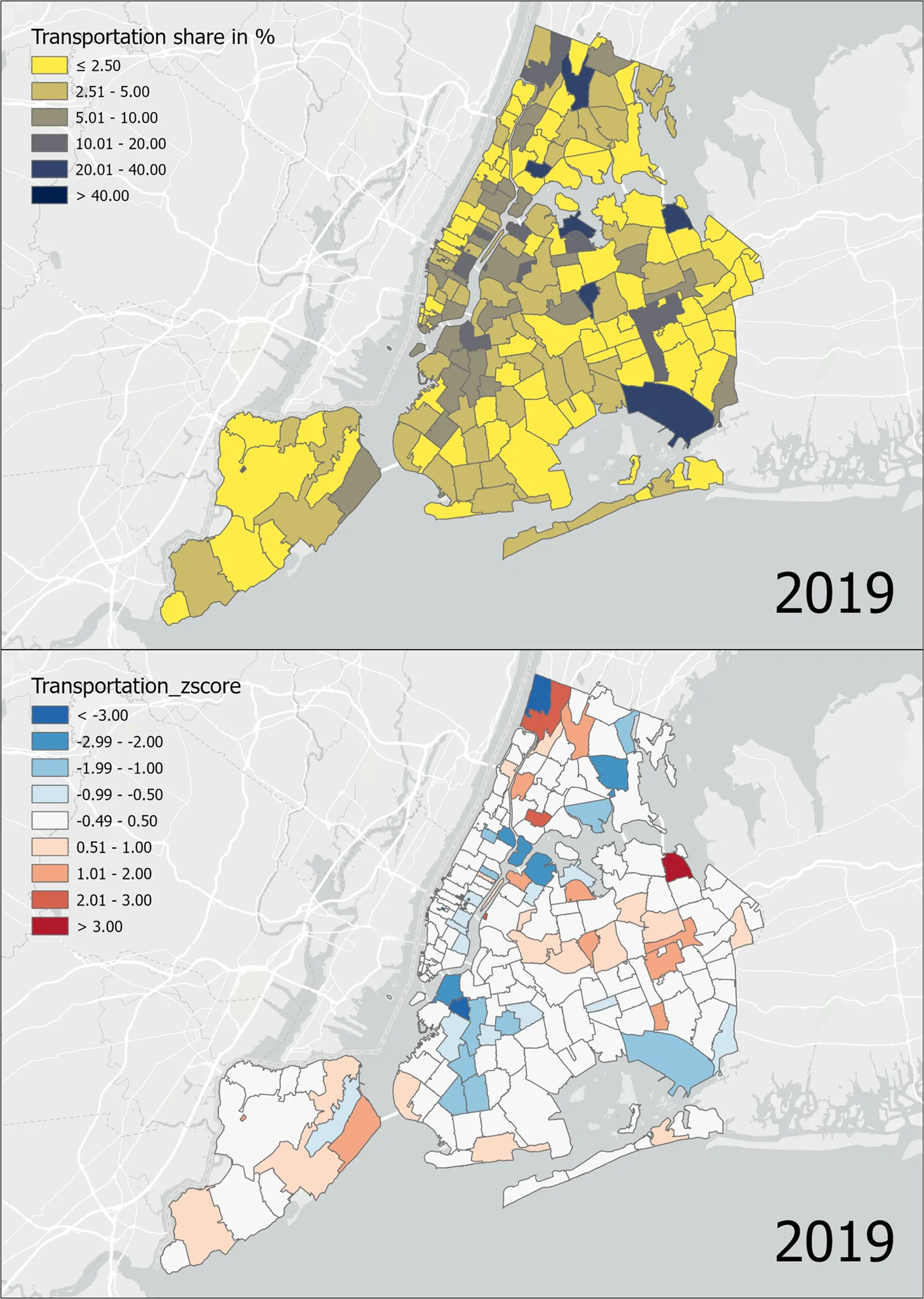
Shares of Transportation Category (top) and Deviation of Shares (bottom) from All-Year Average in NYC 2019

**Fig. 20:**
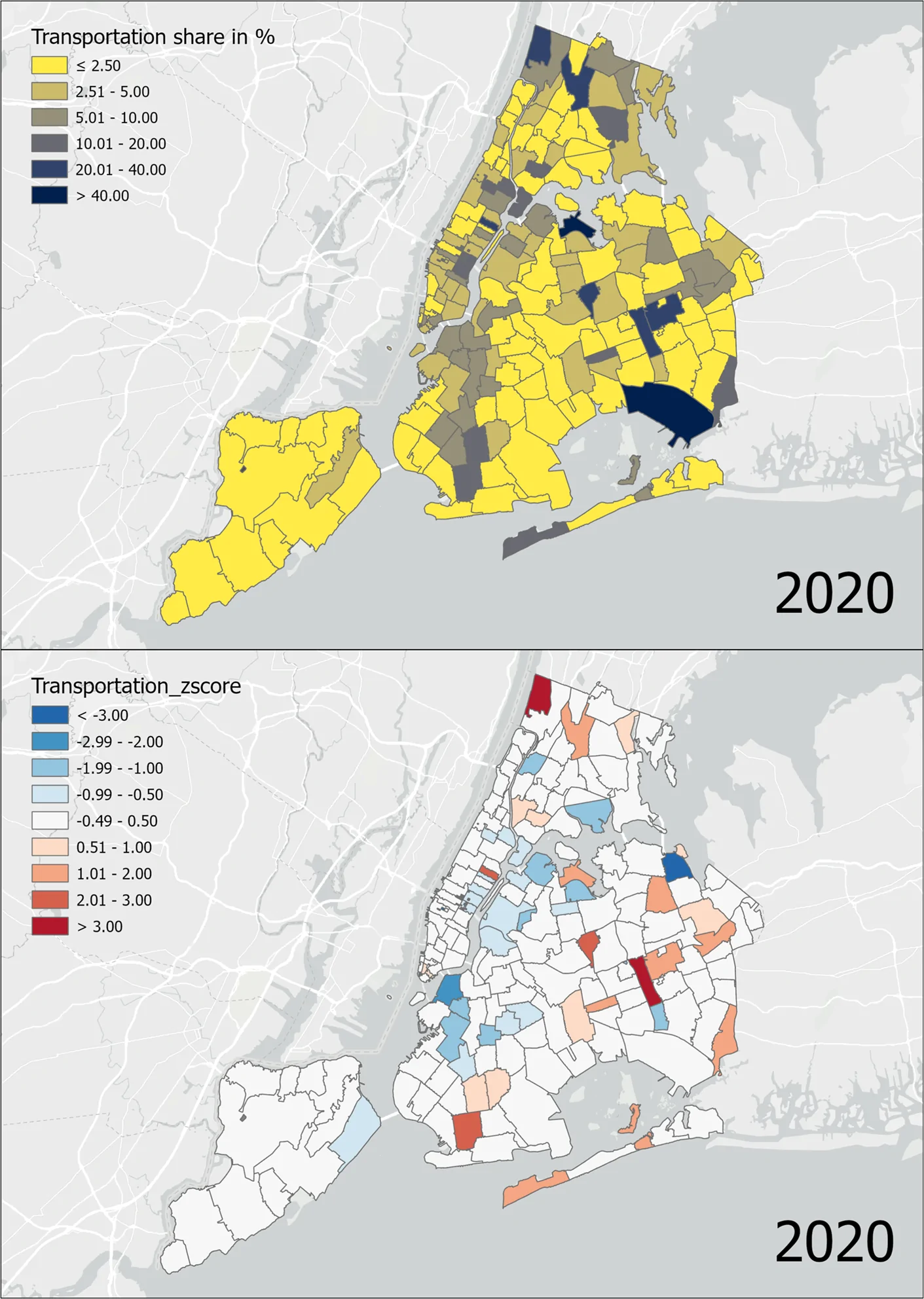
Shares of Transportation Category (top) and Deviation of Shares (bottom) from All-Year Average in NYC 2020

**Fig. 21:**
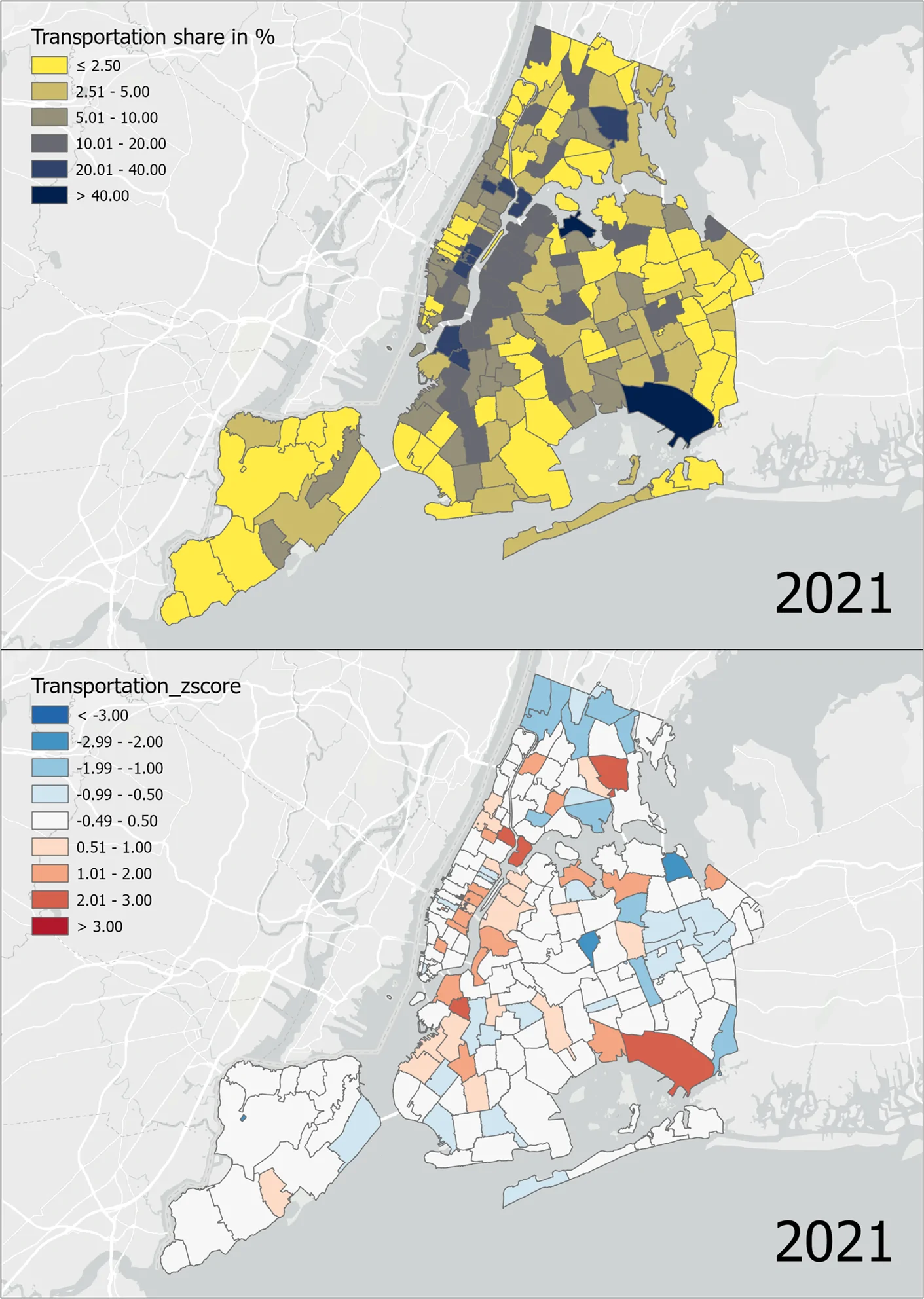
Shares of Transportation Category (top) and Deviation of Shares (bottom) from All-Year Average in NYC 2021

**Fig. 22:**
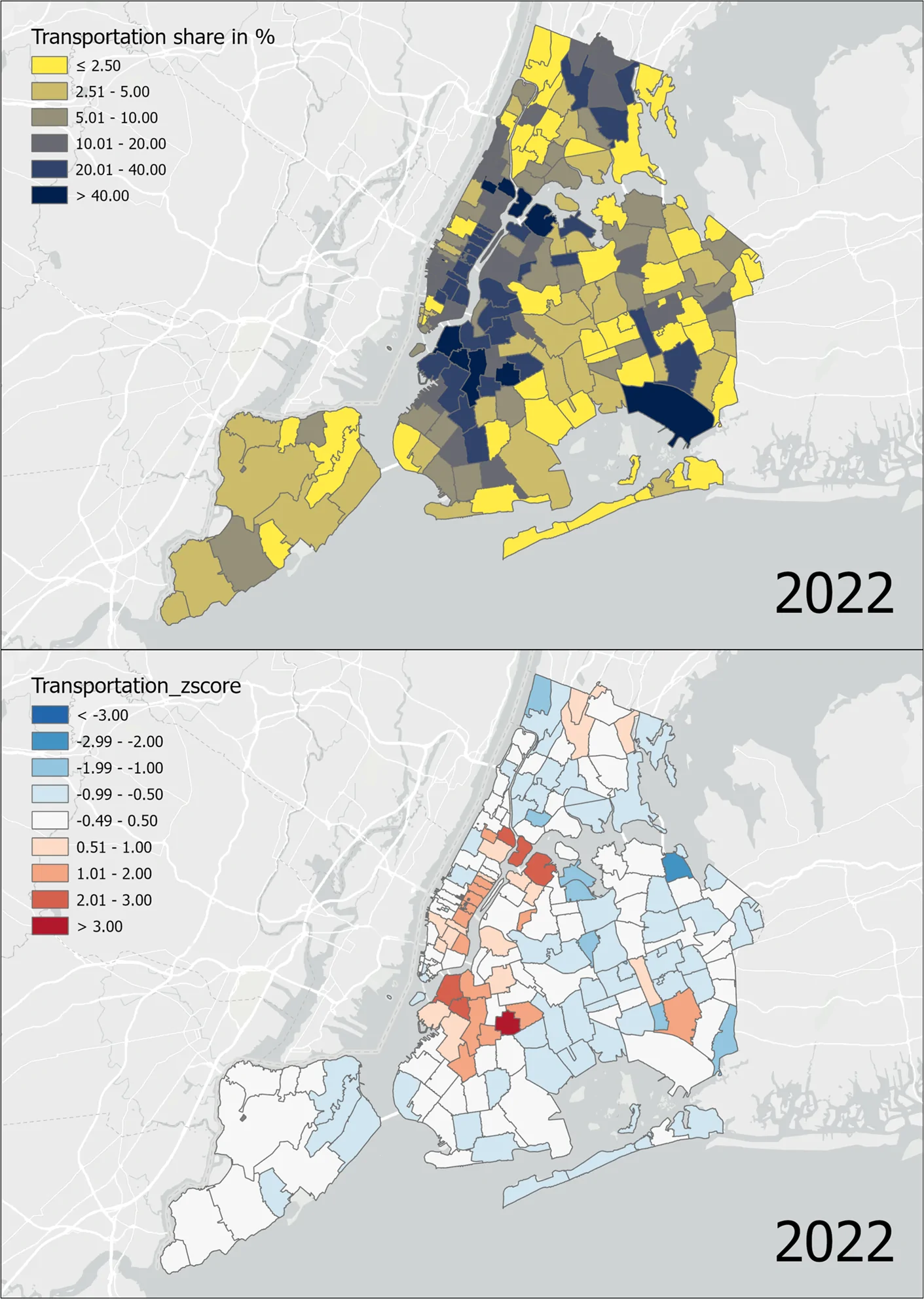
Shares of Transportation Category (top) and Deviation of Shares (bottom) from All-Year Average in NYC 2022

**Fig. 23:**
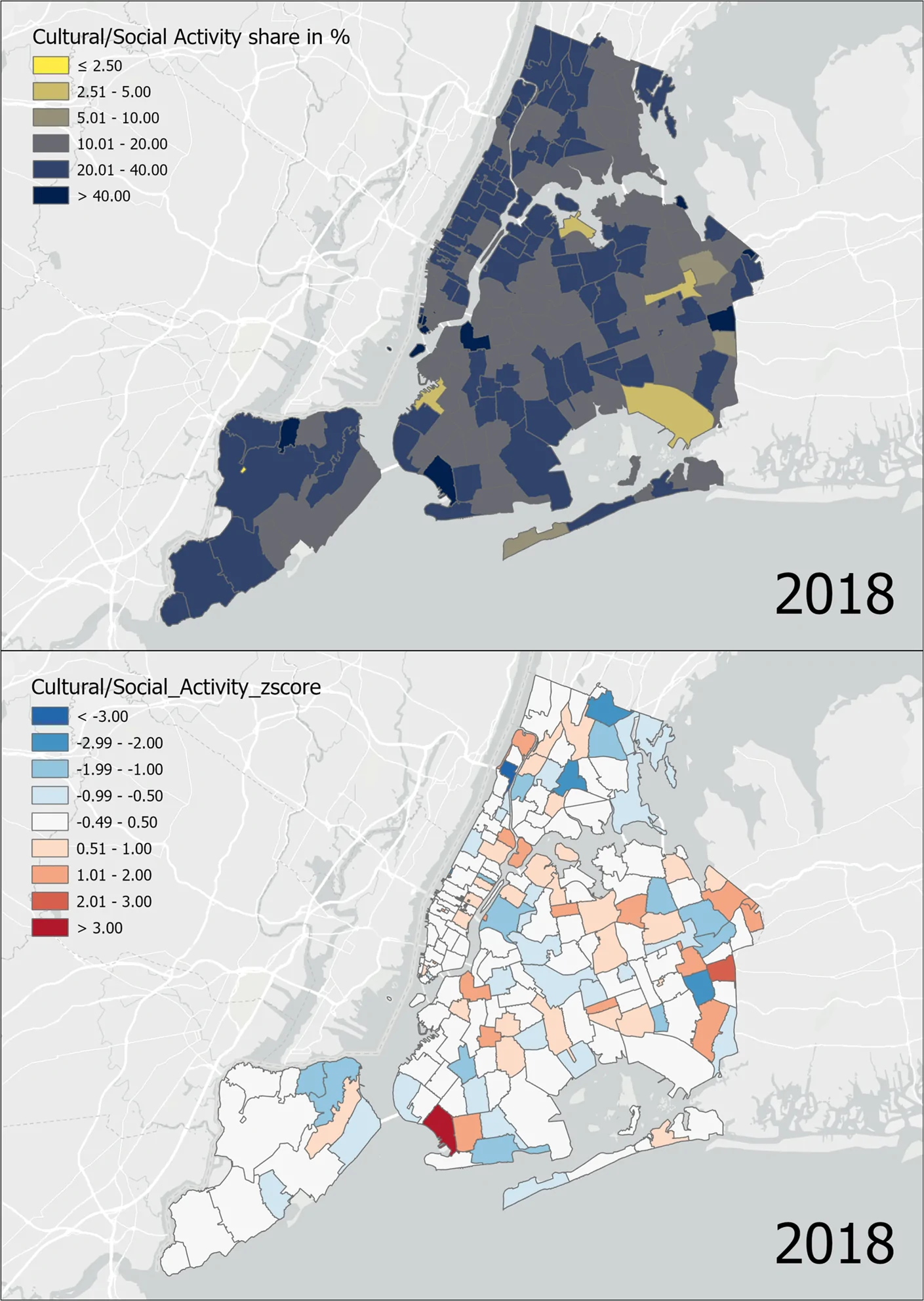
Shares of Cultural/Social Activity Category (top) and Deviation of Shares (bottom) from All-Year Average in NYC 2018

**Fig. 24:**
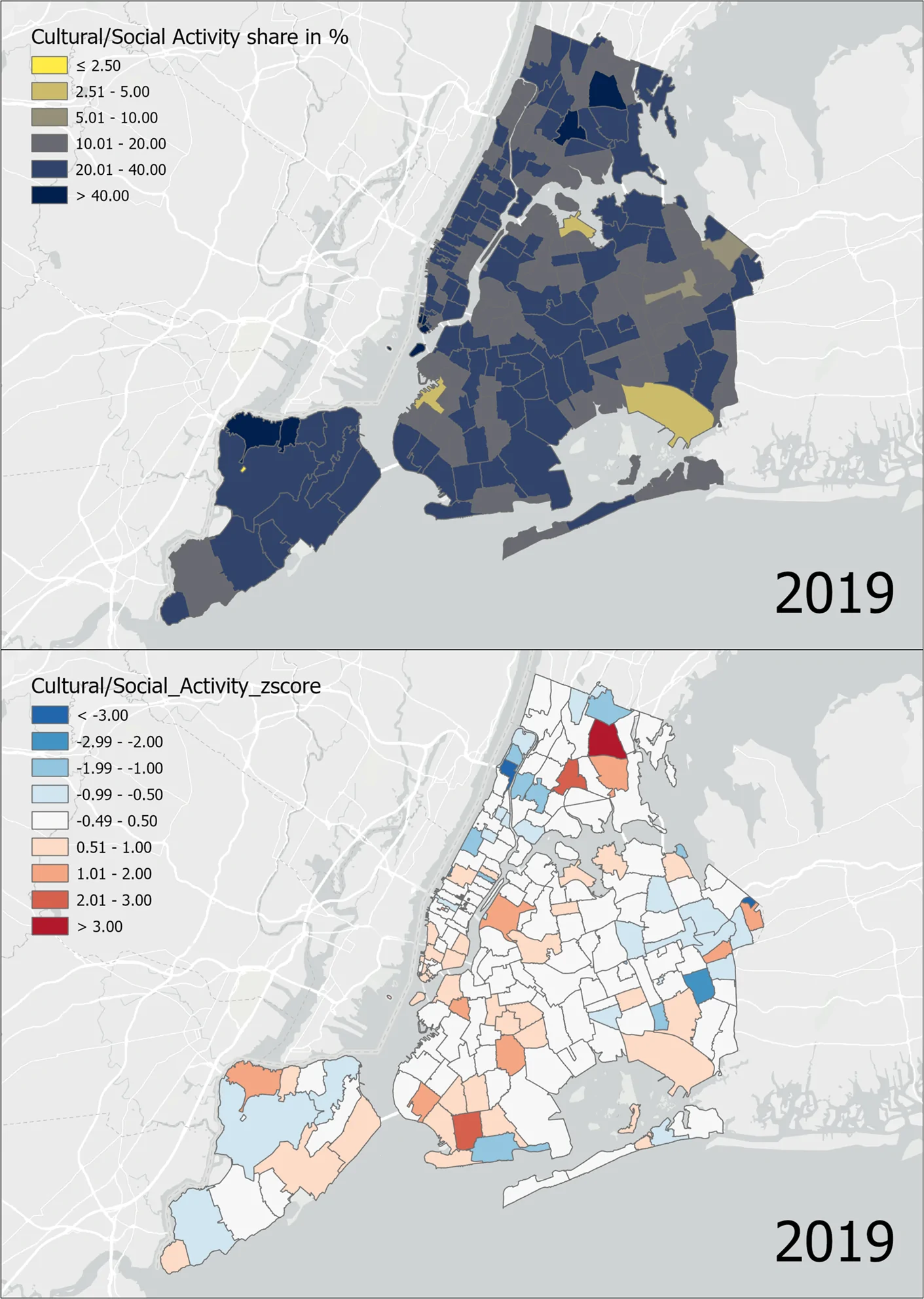
Shares of Cultural/Social Activity Category (top) and Deviation of Shares (bottom) from All-Year Average in NYC 2019

**Fig. 25:**
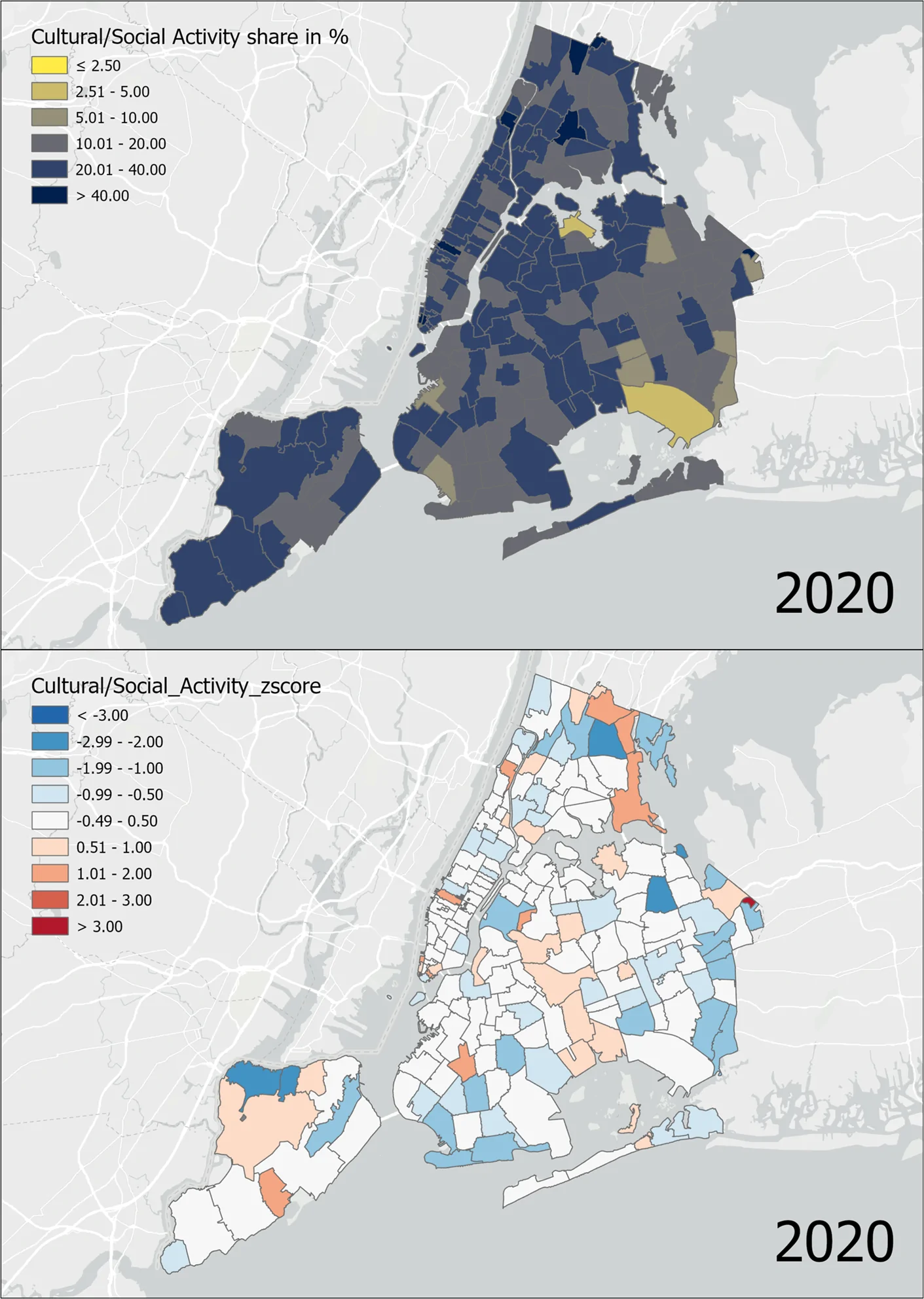
Shares of Cultural/Social Activity Category (top) and Deviation of Shares (bottom) from All-Year Average in NYC 2020

**Fig. 26:**
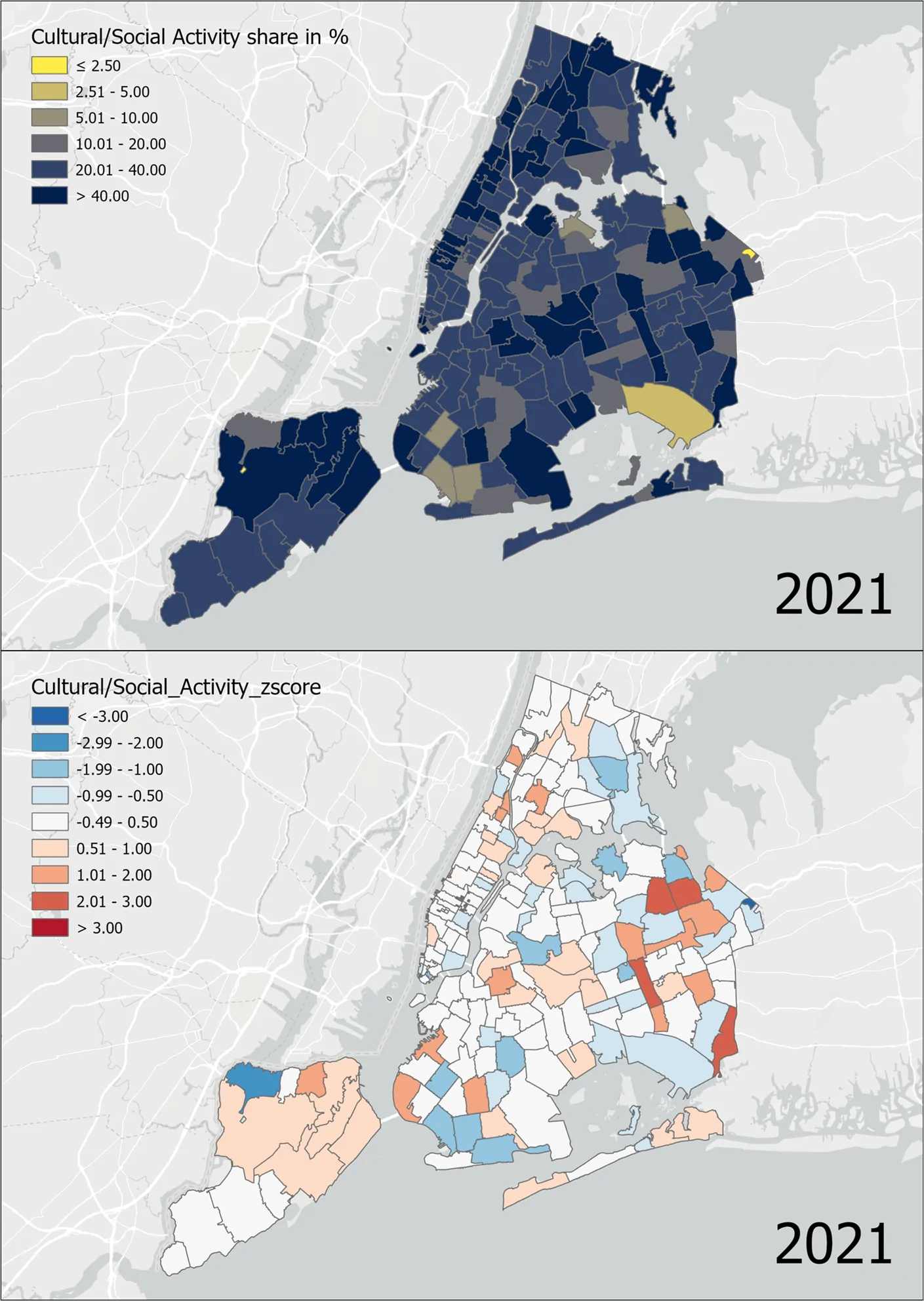
Shares of Cultural/Social Activity Category (top) and Deviation of Shares (bottom) from All-Year Average in NYC 2021

**Fig. 27:**
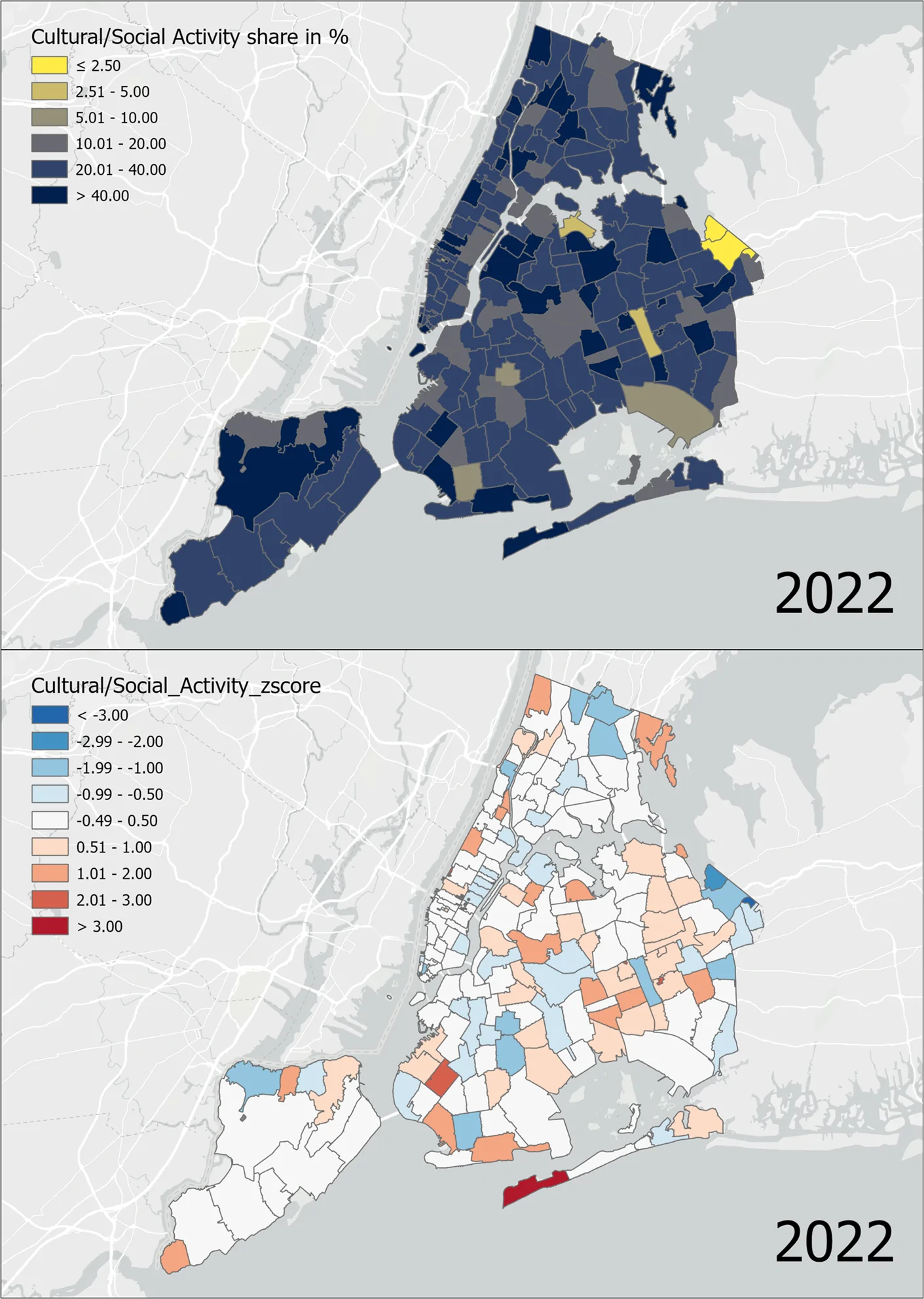
Shares of Cultural/Social Activity Category (top) and Deviation of Shares (bottom) from All-Year Average in NYC 2022

**Fig. 33:**
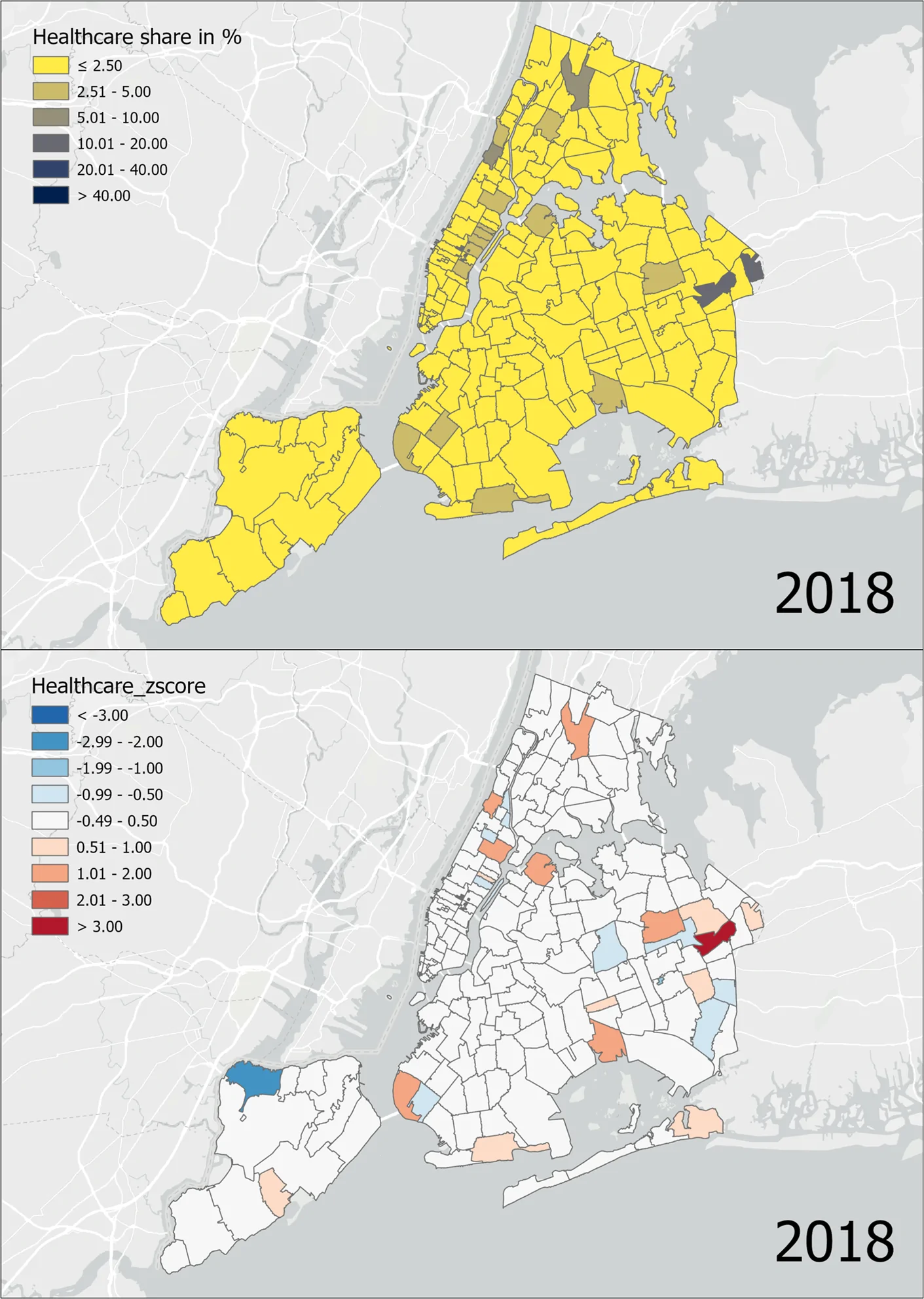
Shares of Healthcare Category (top) and Deviation of Shares (bottom) from All-Year Average in NYC 2018

**Fig. 34:**
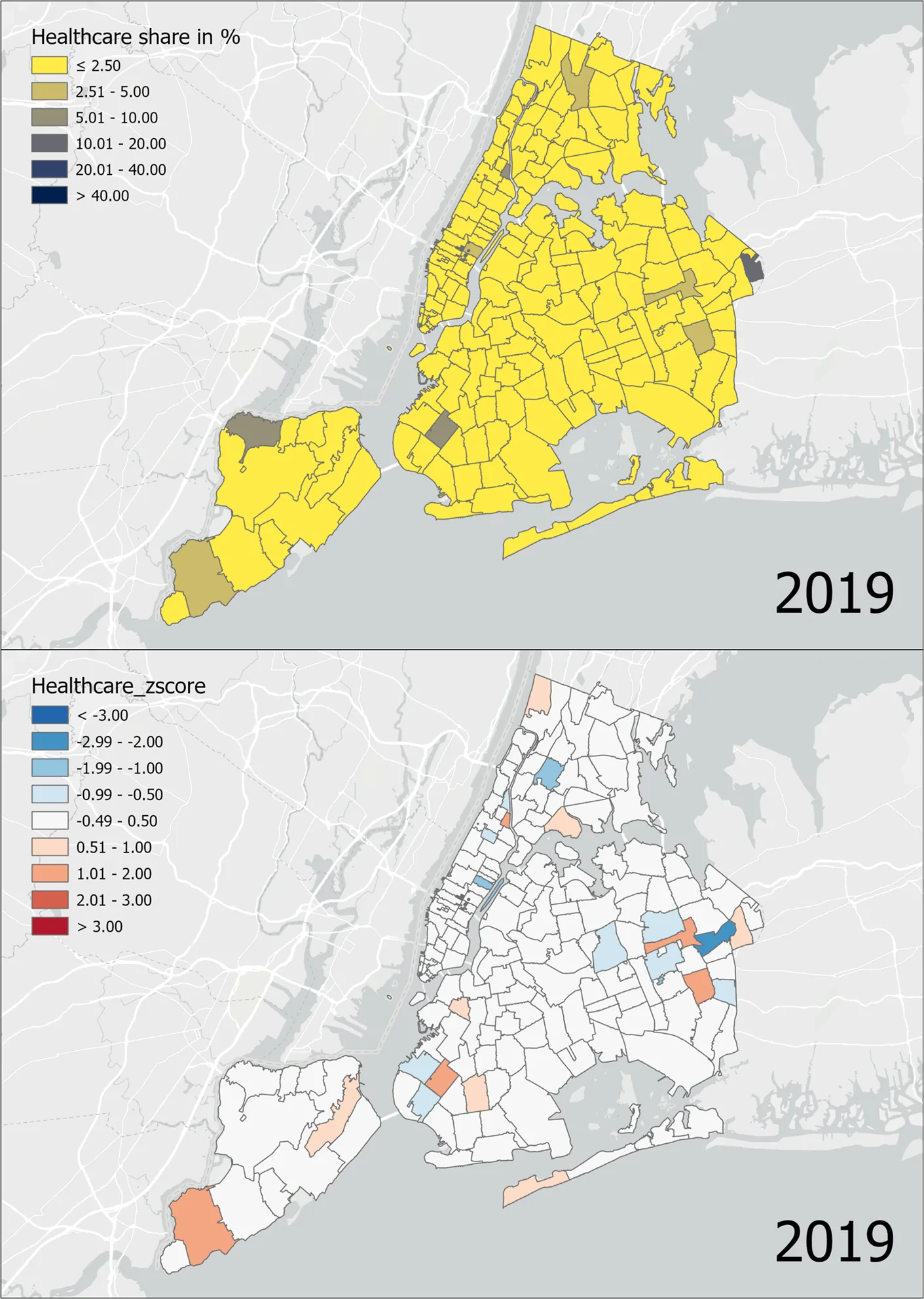
Shares of Healthcare Category (top) and Deviation of Shares (bottom) from All-Year Average in NYC 2019

**Fig. 35:**
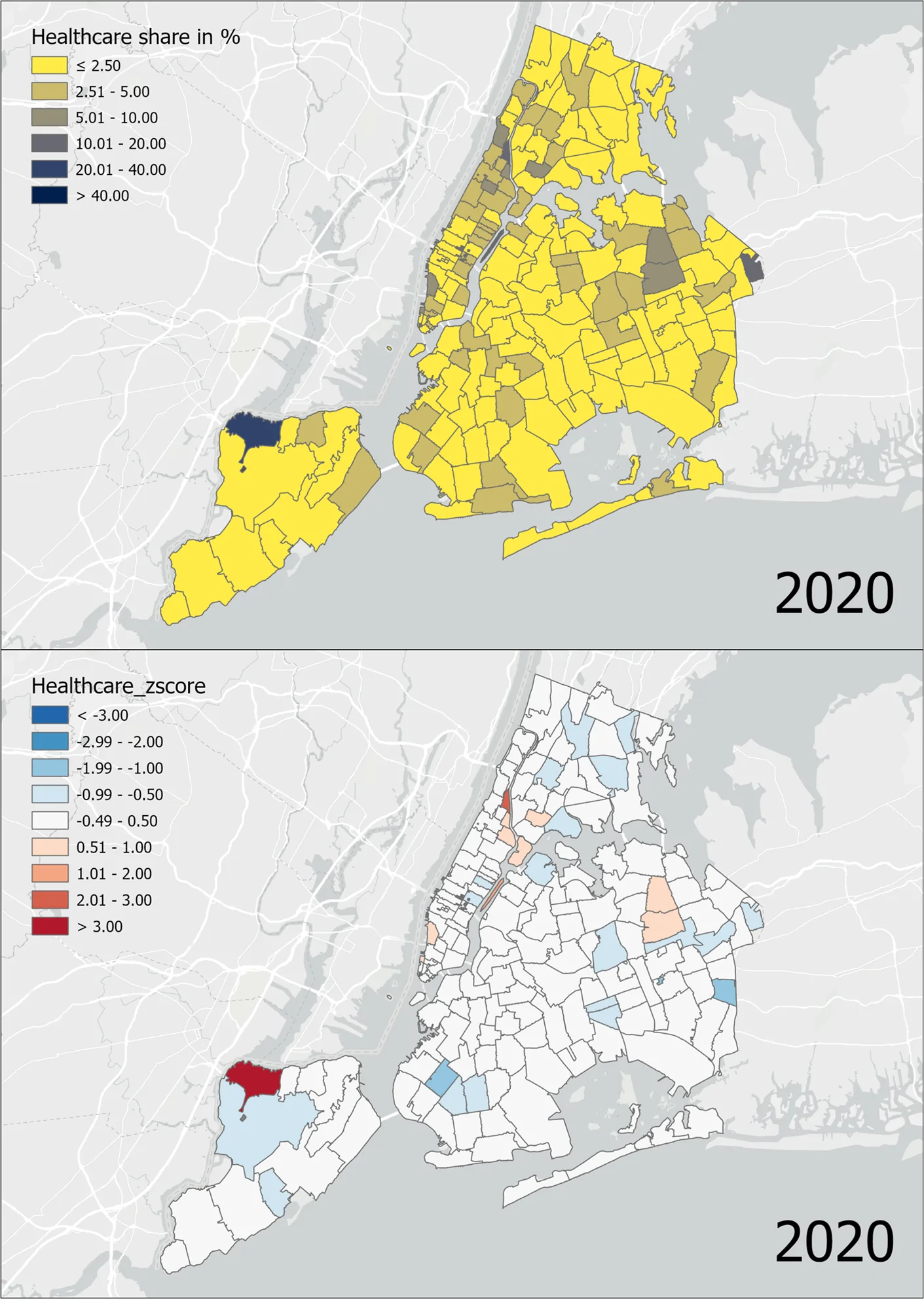
Shares of Healthcare Category (top) and Deviation of Shares (bottom) from All-Year Average in NYC 2020

**Fig. 36:**
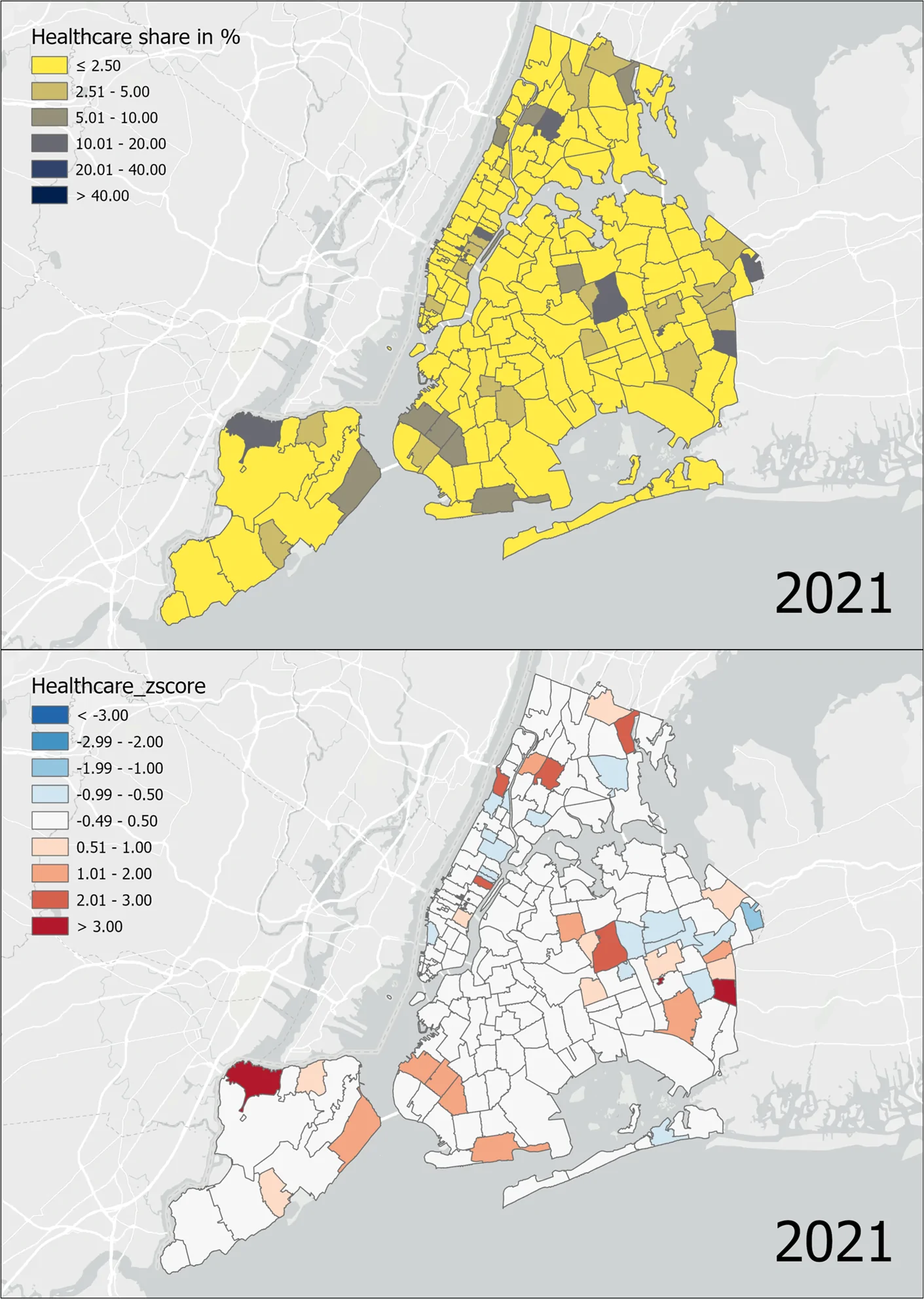
Shares of Healthcare Category (top) and Deviation of Shares (bottom) from All-Year Average in NYC 2021

**Fig. 37:**
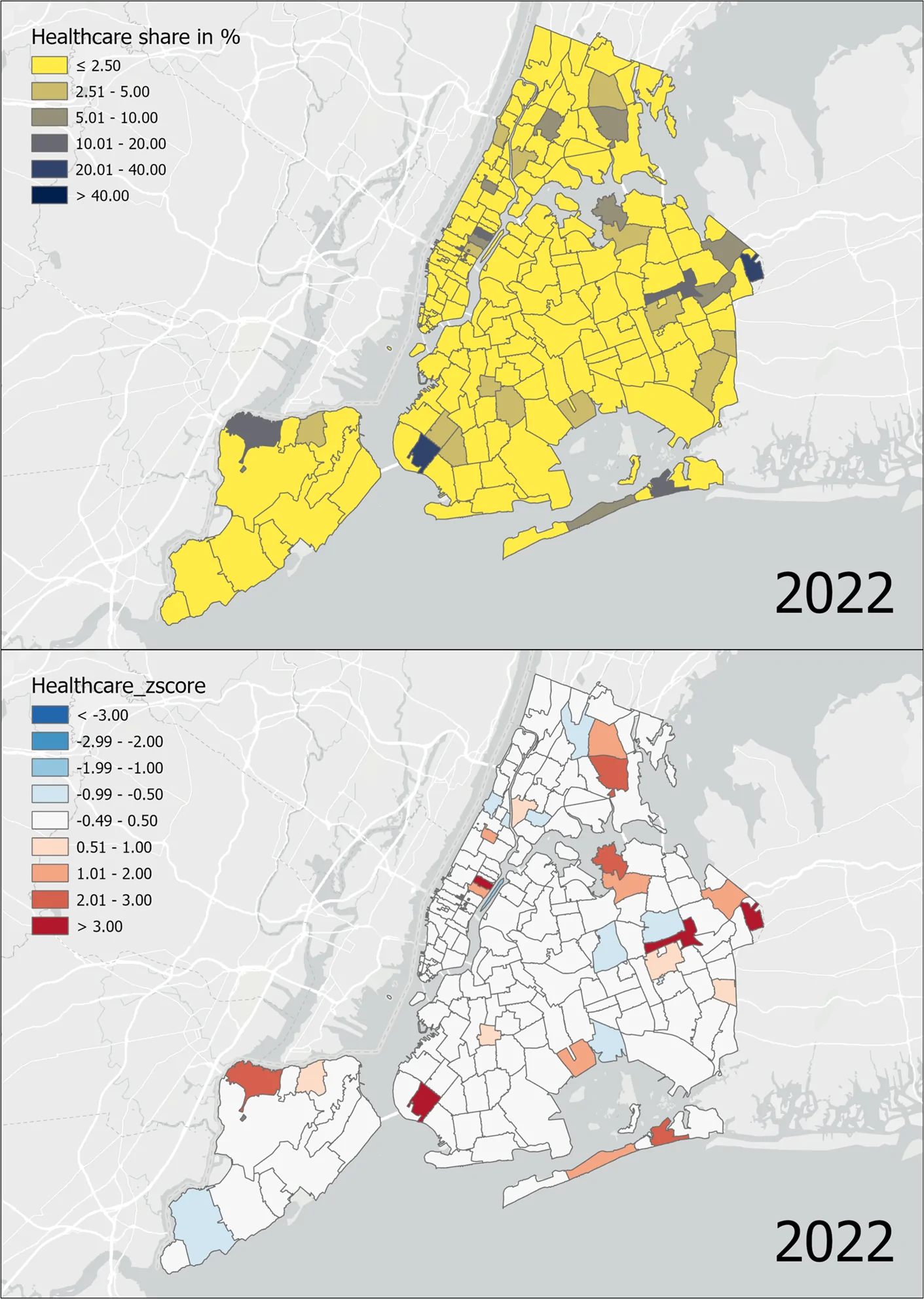
Shares of Healthcare Category (top) and Deviation of Shares (bottom) from All-Year Average in NYC 2022

**Fig. 38:**
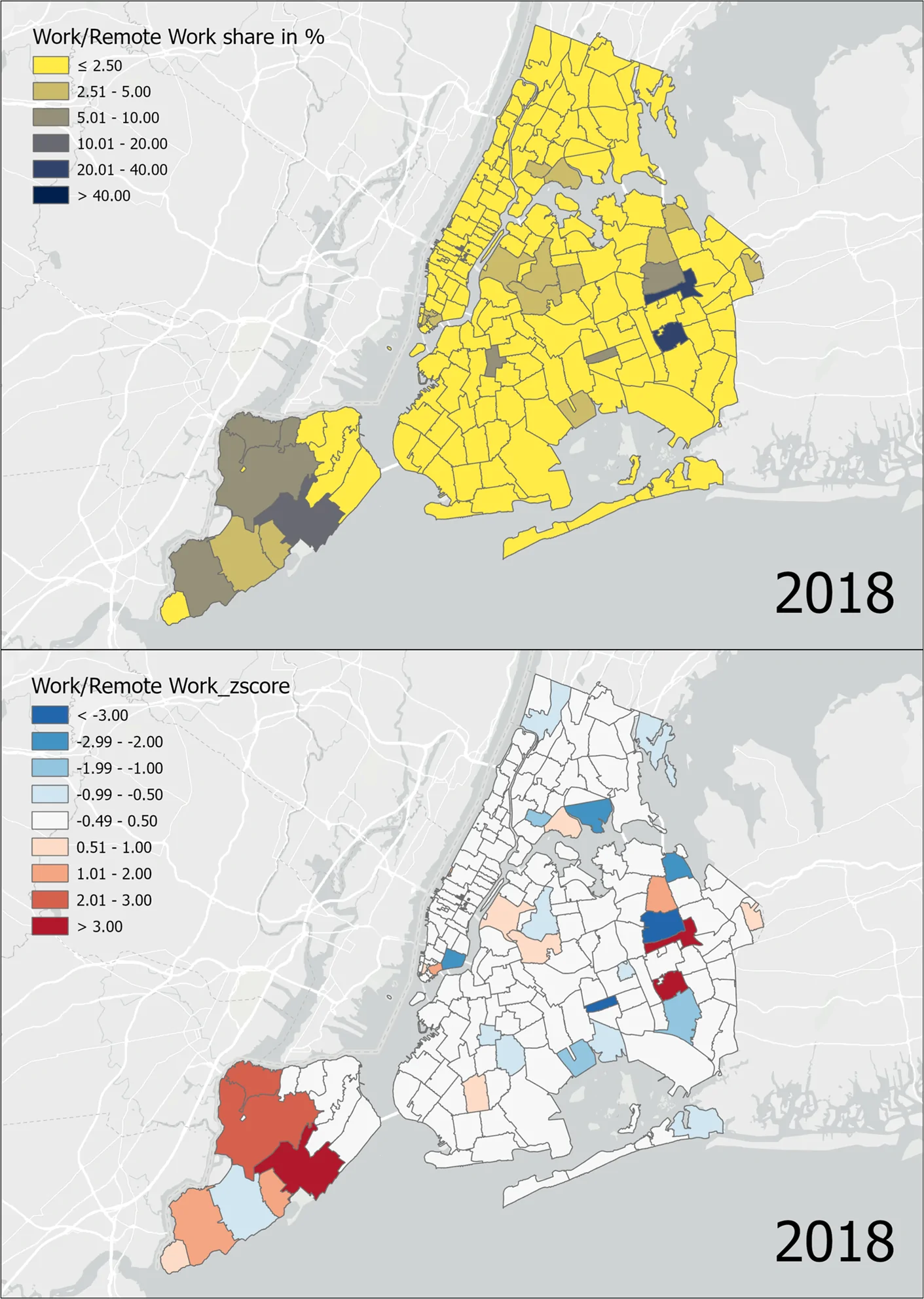
Shares of Work/Remote Work Category (top) and Deviation of Shares (bottom) from All-Year Average in NYC 2018

**Fig. 39:**
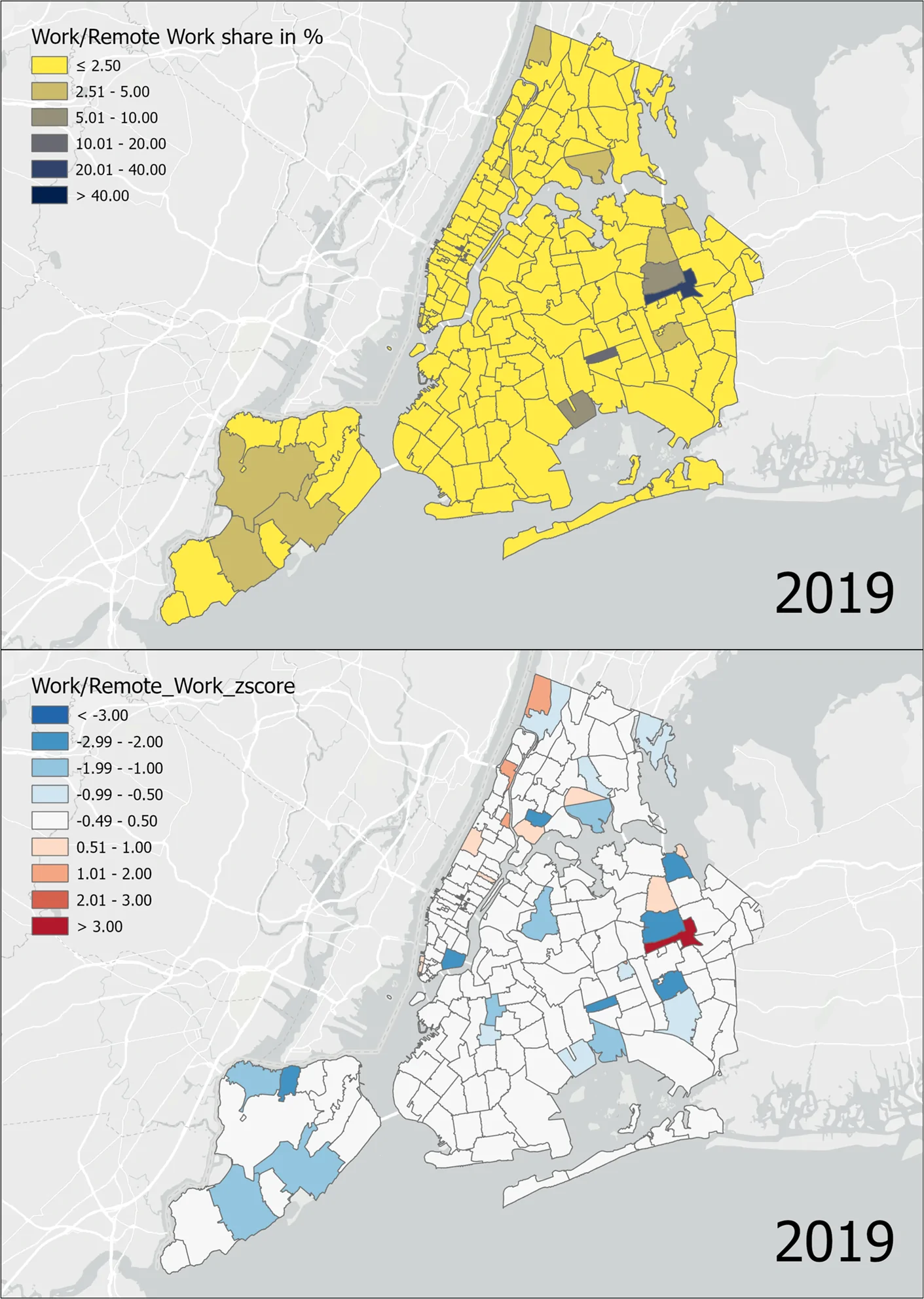
Shares of Work/Remote Work Category (top) and Deviation of Shares (bottom) from All-Year Average in NYC 2019

**Fig. 40:**
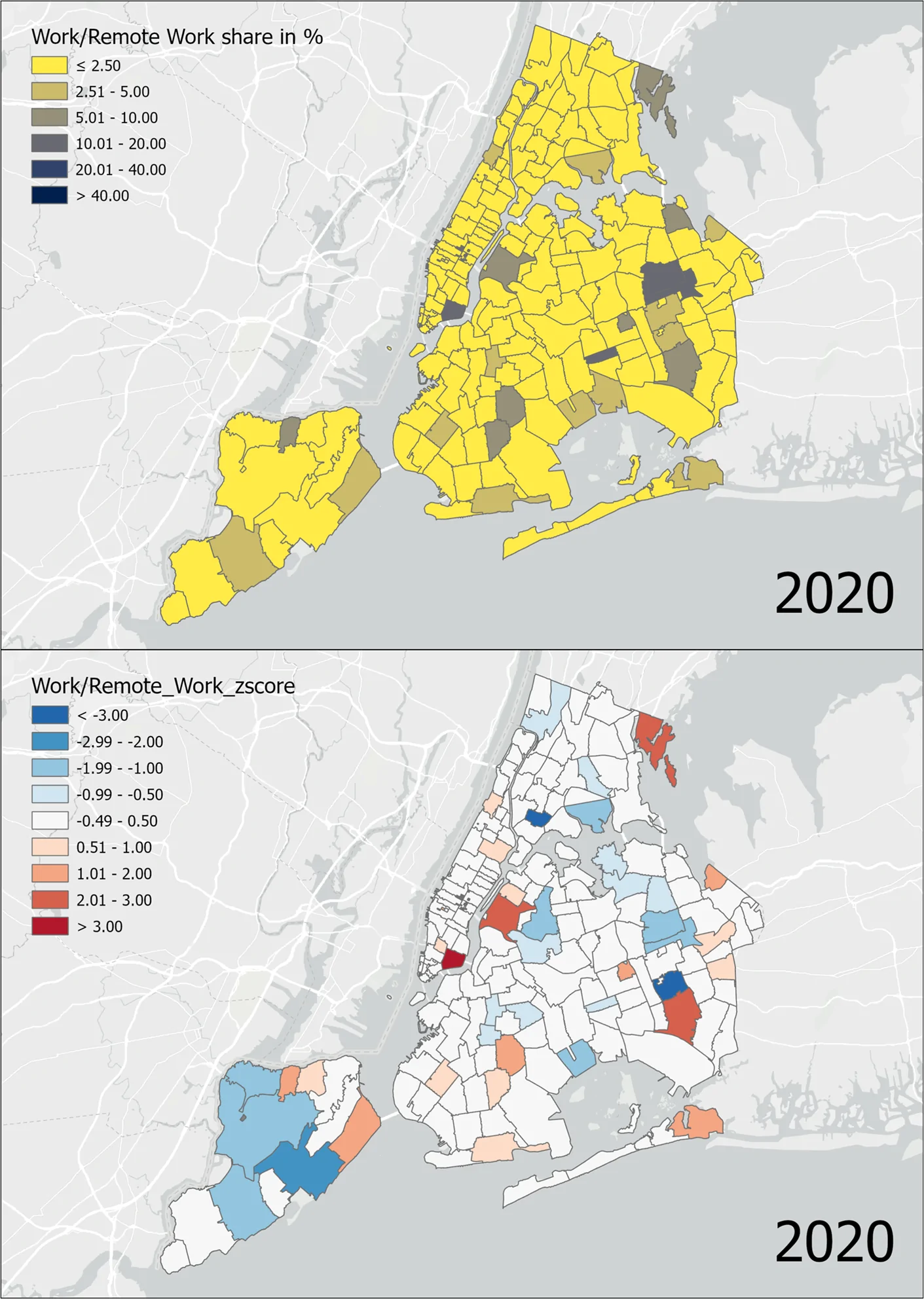
Shares of Work/Remote Work Category (top) and Deviation of Shares (bottom) from All-Year Average in NYC 2020

**Fig. 41:**
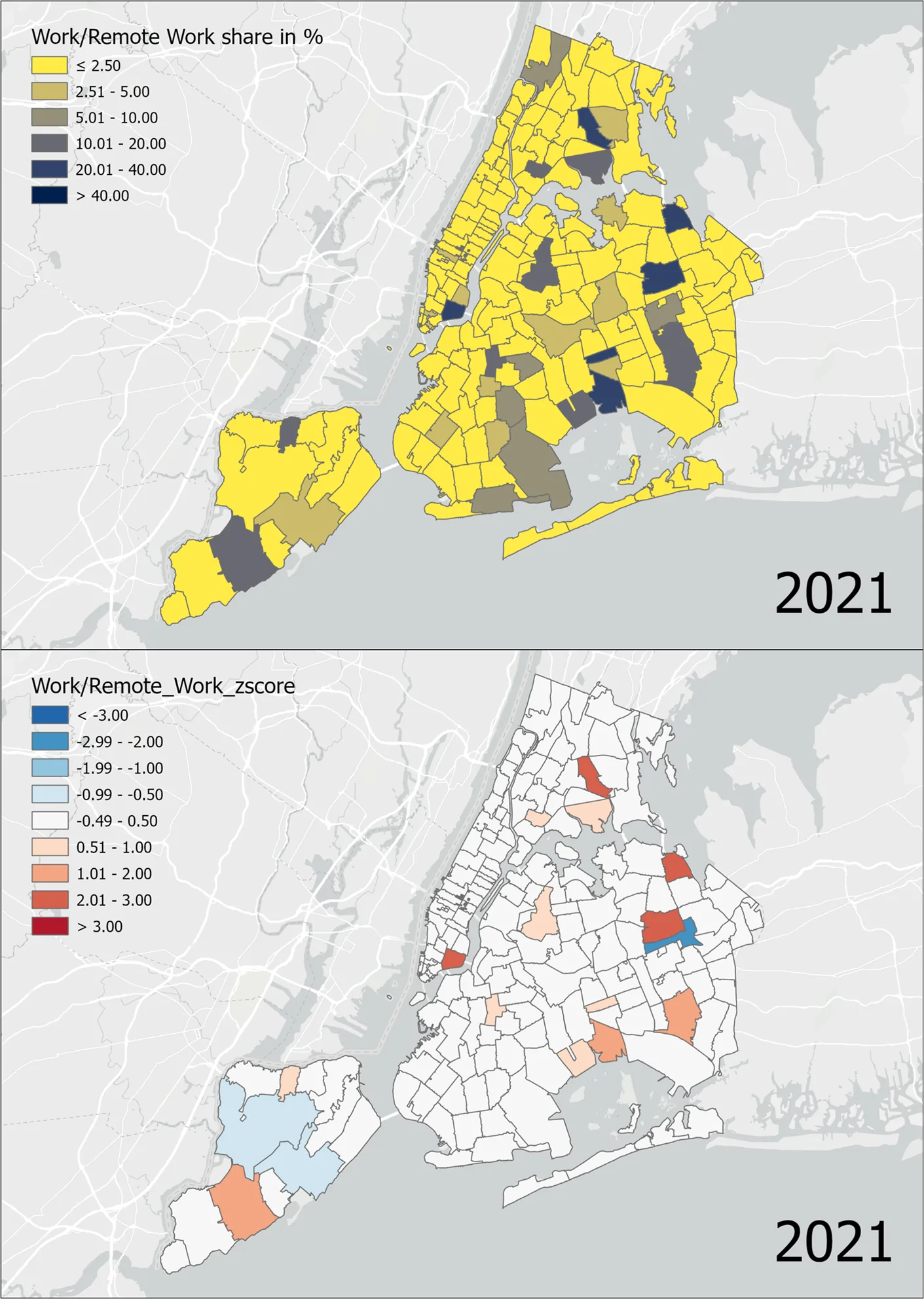
Shares of Work/Remote Work Category (top) and Deviation of Shares (bottom) from All-Year Average in NYC 2021

**Fig. 42:**
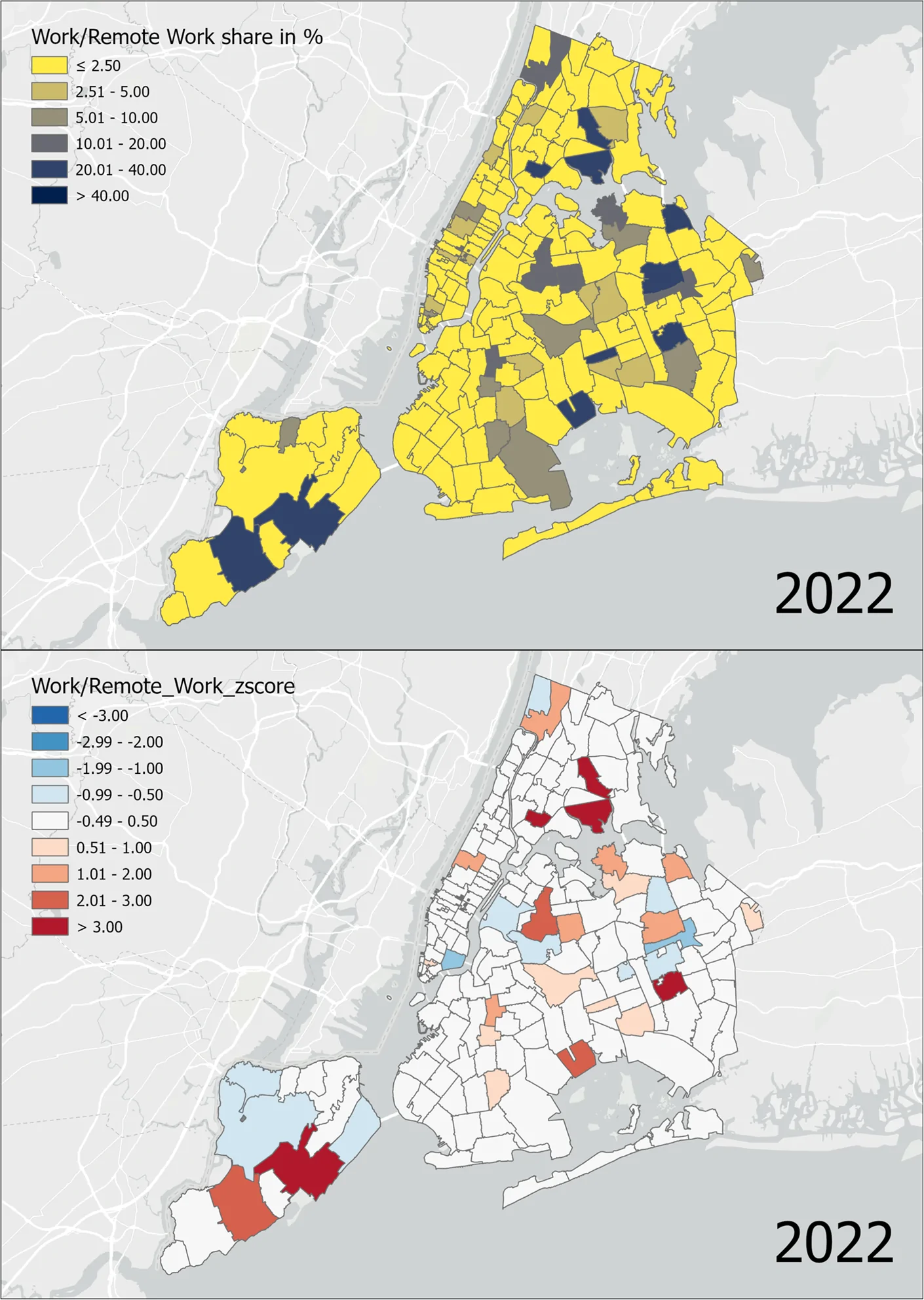
Shares of Work/Remote Work Category (top) and Deviation of Shares (bottom) from All-Year Average in NYC 2022

## Data Availability

Data cannot be shared openly but are available upon request from the authors.

## Appendix

**Table 3:** Parameter settings used for BERTopic modeling

Component	Parameter	Value
Embedding model	embedding_model	all-MiniLM-L6-v2
CountVectorizer	ngram_range	(1, 2)
CountVectorizer	stop_words	english
CountVectorizer	min_df	10
UMAP	n_neighbors	15
UMAP	n_components	5
UMAP	min_dist	0.0
UMAP	metric	cosine
UMAP	low_memory	True
UMAP	random_state	42
HDBSCAN	min_cluster_size	10
HDBSCAN	metric	euclidean
HDBSCAN	cluster_selection_method	eom
HDBSCAN	prediction_data	True
BERTopic	calculate_probabilities	False

**Table 4:** Global Moran’s I results of share deviation from all-year average (all categories)

		2018	2019	2020	2021	2022
Healthcare	Moran’s Index	0.045616	0.012571	0.08286	0.017053	0.025729
	z-Score	1.866941	0.699192	3.137028	0.741009	1.058774
	-value	0.06191	0.484432	0.001707	0.458688	0.289703
Transportation	Moran’s Index	0.082443	0.000329	0.027424	0.026214	0.21543
	z-Score	3.019604	0.174993	1.10013	1.030507	7.505692
	*p*-value	0.002531	0.861085	0.271276	0.302772	< 0.001
Retail Activity	Moran’s Index	0.037496	0.110334	-0.02103	0.027659	0.03555
	z-Score	1.425845	3.940511	-0.548374	1.136133	1.393332
	*p*-value	0.153913	0.000081	0.583435	0.255901	0.163519
Work/Remote Work	Moran’s Index	0.052758	-0.016481	-0.016693	-0.008383	-0.029282
	z-Score	2.030953	-0.425373	-0.421837	-0.147424	-0.876876
	*p*-value	0.04226	0.670565	0.673144	0.882798	0.380554
Cultural/Social Activity	Moran’s Index	-0.0039	0.053107	0.067381	0.005039	0.094271
	z-Score	0.029215	1.967775	2.409147	0.328138	3.30508
	*p*-value	0.976693	0.049094	0.01599	0.742807	0.000949

### Final curated list of cultural/social activity Wikipedia articles

“Entertainment”, “Culture”, “Society”, “Drama”, “Performance”, "Court (royal)", “Banquet”, “Party”, “Amusement”, “Fun”, “Ceremony”, “Religious festival”, “Satire”, “Recreation”, “Leisure”, "Play (theatre)", “Opera”, “Television show”, “Game”, “Theatre”, “Concert”, "Magic (illusion)", “Video game”, “Festival”, “Music festival”, “Film festival”, “Competitive dance”, “Spectator sport”, “Cooking”, “Remix”, “Camping”, “Circus”, “Music hall”, "Theater (structure)", “Auditorium”, “Spectacle”, “Racing”, “Tournament”, “Musical instrument”, “Jazz”, “Folk music”, “Choir”, “Musical ensemble”, “Human voice”, “A cappella”, “Operetta”, “Home cinema”, "History of film", “Parade”, “Fair”, “Fandom”, “Social relation”, “Friendship”, “Social phenomenon”, “Social conflict”, “Social roles”, “Social behavior”, “Social norm”, “The arts”, “Socialization”, “Cultural norm”, “Culture change”, “UNESCO”, “Anthropology”, “Cultural universals”, “Art”, “Music”, “Dance”, “Ritual”, “Religion”, “Mythology”, “Philosophy”, “Literature”, “Writing”, “Oral literature”, “Science”, “Social class”, “Popular culture”, “Folk culture”, “Cultural capital”, “Media culture”, “Tradition”, “Cultural anthropology”, “Cultural relativism”, “Social interaction”, “Cultural change”, “Social innovation”, “Social revolution”, “Cultural invention”, “Social structure”, "European classical music", “Social group”, “Western culture”, “Archaeology”, “Sociology”, "Non-material culture", “Social stratification”, “Social network”, “Social psychology”, “Social theory”, “Film theory”, “Museum studies”, “Art history”, “Film”, “Photography”, “Fashion”, “Hairstyle”, “Cultural artifact”, “Culture shock”, “LGBT culture”, “Cultural heritage”, “Cultural diversity”

### Final curated list of work/remote work Wikipedia articles

"Work_(human_activity)", “Job”, “Remote_work”, “Office”, “Coworking”, "Job characteristic theory", “Job satisfaction”, “Employee engagement”, "Work-family conflict", “Turnover intention”, “Work motivation”, “Labour inspectorate”, “Payment”, “Unpaid work”, “Manufacturing”, “Corporation”, “Profession”, “Job titles”, “Career”, "Division of labor", "Critique of work", “Bullshit Jobs”, "The Abolition of Work", “Retirement”, "Universal basic income", “Employment”, “Night shift”, “Slavery”, “Working class”, "Hours of work", “Child labour”, “Office work”, “Seniority”, “Apprentice”, “Journeyman”, “Master craftsman”, “Skilled trade”, “Managerial”, “Manual labor”, “Wage labor”, “Piece work”, “Overwork”, "White-collar worker", “Sedentary”, “Handicraft”, "Computer (job description)", “Labor union”, “Labor market”, “Corporate”, “Resource allocation”, “Work ethic”, "Protestant work ethic", “Slave labor”, “Human trafficking”, “Unemployment”, “Productivity”, “Job matching”, “Unemployment insurance”, “Job guarantee”, “Cheap labour”

### Final curated list of retail activity Wikipedia articles

“Retail”, “Goods”, "Service (economics)", “Consumer”, “Wholesaling”, “Institutional customers”, “Manufacturing”, "Profit (accounting)", “Supply chain”, “Peddler”, "Bricks and mortar store", “Online shopping”, “Corporate strategy”, "Market (economics)", "Product (business)", “Customer service”, “Wholesale”, “Vending machine”, "Consumers%27 co-operative", “Department store”, "E-commerce", “Retail apocalypse”, “Marketing mix”, “Product management”, “Site selection”, "Online marketing platform", “Pricing strategies”, "Value-based pricing", "Relationship-based pricing", "Everyday low price", “Product bundling”, “Psychological pricing”, "Money back guarantee", "Buy one, get one free", “Servicescape”, “Marketplace”, “Retail chains”, “Services marketing”, “Disposable product”, “Clothing”, “Fabrics”, “Footwear”, “Toiletries”, “Cosmetics”, “Medicines”, “Stationery”, “Grocery store”, “Supermarkets”, “Hypermarkets”, “Convenience stores”, “Automobiles”, “Home appliance”, “Electronics”, “Furniture”, “Sporting goods”, “Lumber”, "Contemporary art galleries", “Bookstores”, “Handicrafts”, “Music store”, “Gift shops”, "Barriers to entry", "National Retail Federation", “Consumer spending”, "Gross domestic product", “Food service”

### Final curated list of transportation Wikipedia articles

“Transportation”, “Traffic”, "Mode of transport", “Aviation”, “Land transport”, “Rail transport”, “Road transport”, “Ship transport”, “Cable transport”, “Pipeline transport”, “Space transport”, “Infrastructure”, “Vehicle”, “Road”, “Railway”, "Airway (aviation)", “Waterway”, “Airport”, “Train station”, “Bus station”, “Warehouse”, “Fuel station”, “Seaport”, "Means of transport", “Wagons”, “Automobiles”, “Bicycles”, “Buses”, “Train”, “Truck”, “Helicopter”, “Watercraft”, “Spacecraft”, "Fixed-wing aircraft", “Public transport”, “Private transport”, “Air pollution”, “Land use”, “Traffic flow”, “Urban sprawl”, “Sustainable transport”, “Bicycle”, “Rotorcraft”, “Gyroplane”, “Airliner”, "Lift (soaring)", “Landing”, "Rail tracks#Railway rail", “Railroad tie”, “Rail gauge”, “Monorail”, "Maglev (transport)", “Locomotive”, “Steam locomotive”, “Diesel locomotive”, “Electric locomotive”, "Railway electrification system", "Inter-city rail", "High-speed rail", “Regional rail”, “Commuter rail”, “Tram”, “Rapid transit”, “Freight trains”, “Box car”, “Container train”, “Route number”, “Trail”, “Bus”, “Motorcycles”, “Pedestrians”, “Noise pollution”, "Last mile (transportation)", “Barge”, “Boat”, “Ship”, “Sailboat”, "Hull (watercraft)", “Steam ships”, “Submarine”, “Sea transport”, “Commercial vessel”, “Shipping”, "Short sea shipping", “Ferry”, “Aerial tramway”, “Sea lane”, "Airport rail link", “Parking lot”, “Transshipment”, “Transport finance”, “Driving”, “People mover”, “Passenger”, “Flag carrier”, “Railway company”, “Commuting”, “Business travel”, "Intermodal passenger transport", “Transport hub”, “Railway station”, "Demand-responsive transport", “International travel”, “Cargo airline”, “Transport sustainability”, “Transport forecasting”, “Transport economics”, “Transport engineering”, “Trip generation”, “Trip distribution”, “Mode choice”, “Route assignment”, “Transport electrification”, “Electric car”, “Airplane”, “Railroads”, “Roads”, “Vehicles”, “Trains”, “Public conveyance”, "Intersection (road)", "International Regulations for Preventing Collisions at Sea", “Lane”, "Junction (traffic)", "Interchange (road)", “Traffic signal”, “Traffic cone”, “Traffic sign”, “Motor vehicle”, “Car”, “Moped”, “Speed limit”, “Travel safety”, “Road construction”, “Car accident”, "Debris in the roadway", “Traffic wave”, “Gridlock”, “Network traffic”, “Air traffic”, “Driving etiquette”, "Vienna Convention on Road Traffic", “Traffic light”, "Road traffic control", “Highway Code”, “Traffic code”, "Uniform Vehicle Code", “Traffic law”, “Stop sign”, "Vienna Convention on Road Signs and Signals", "Road traffic control device", “Road marking”, “Turn signal”, “Roundabout”, "Priority to the right", “Traffic circle”, “Boulevard rule”, "All-way stop", “Protected intersection”, "Bicycle-friendly", “Pedestrian crossings”, “Jaywalking”, “Green wave”, “Divided highway”, “Overtaking”, "Left- and right-hand traffic#Left-hand traffic", "Left- and right-hand traffic#Right-hand traffic", "Overtaking#Rules of overtaking", “Road rage”, "California Vehicle Code", “Lane splitting”, "Grade separation#Roads", “Grade separation”, “Underpass”, “Interstate highways”, “German Autobahn”, "Expressways of China", “Road works”, “Traffic jam”, "Traffic engineering (transportation)", "Level of service (transportation)", "Three-phase traffic theory", “Rush hour”, “Drive time”, “Public transportation”, “License plate”, "High Occupancy Vehicle Lane", “Emergency service”, “Fire apparatus”, "Winter service vehicle", "Contraflow lane reversal", "Intelligent transportation system", "Floating car data", "Integration of traffic data with navigation systems", "Code of Federal Regulations"

### Final curated list of healthcare Wikipedia articles

Healthcare", “Health”, “Preventive healthcare”, “Diagnosis”, "COVID-19", “Coronavirus”, "SARS-CoV-2", "COVID-19 pandemic", "COVID-19 vaccine", “Long COVID”, “Therapy”, “Cure”, “Disease”, “Illness”, “Injury”, “Disability”, “Health professional”, "Allied health professions", “Medicine”, “Dentistry”, “Pharmacy”, “Midwifery”, “Nursing”, “Optometry”, “Audiology”, “Psychology”, “Occupational therapy”, “Physical therapy”, “Athletic training”, “Health profession”, “Primary care”, “Tertiary care”, “Public health”, “Health policy”, “Health literacy”, “Health system”, "World Health Organization", "Health Human Resources", “Health facilities”, “Physical health”, “Mental health”, "Well-being", "Eradication of infectious diseases", “Smallpox”, “Paraprofessional”, “Physiotherapy”, “Allied health”, "Community health worker", "Unlicensed assistive personnel", "Physical medicine and rehabilitation", "Public health care", "Private healthcare in the United Kingdom", “Health professionals”, “Patients”, "Health care system", "Primary care physician", “General practitioner”, “Family medicine”, “Physiotherapist”, “Physician assistant”, “Nurse practitioner”, “Pharmacist”, “Nurse”, “Patient”, "Referral (medicine)", “Urgent care”, “Multimorbidity”, "Transitional care#continuity", “Preventive medicine”, “Health education”, "International Classification of Primary Care", “Hypertension”, “Diabetes mellitus”, “Asthma”, "Chronic obstructive pulmonary disease", "Major depressive disorder", “Anxiety disorder”, “Back pain”, “Osteoarthritis”, “Thyroid disease”, “Maternal health”, “Family planning”, “Vaccination”, "National Health Interview Survey", "Direct primary care", “Concierge medicine”, “Population aging”, "Non-communicable disease", "Primary health care", “Acute care”, “Hospital”, “Emergency department”, “Childbirth”, "Intensive care medicine", “Medical imaging”, “Psychiatrists”, “Clinical psychology”, “Occupational therapists”, “Dental specialties”, “Physician”, “Health insurance”, “Medical specialty”, “Medical specialist”, "National health insurance", “Respiratory therapists”, “Occupational therapist”, "Speech and language pathology", “Dietitians”, “Inpatient”, "Tertiary referral hospital", “Cancer”, “Neurosurgery”, “Cardiac surgery”, “Plastic surgery”, “Burn”, “Neonatology”, “Clinical research”, “Surgery”, "Self-care", “Home care”, "Long-term care", “Assisted living”, "Substance use disorder", “Prosthesis”, “Orthotics”, “Wheelchair”, "Health care ratings", “Evaluation”, “Healthcare industry”, “Biotechnology”, “Biopharmaceutical”, “Medical model”, “Medical diagnosis”, “Biomedical research”, “Pharmaceutical research”, "Evidence-based medicine", "Evidence-based practice", "Health services research", "Social model of disability", "Health care systems", "Health care industry", “Life expectancy”, "Health system#International comparisons", “Health administration”, “Health department”, "Health professional requisites".

### Curated keywords cultural/social activity

“accompaniment”, “activities”, “activity”, “actors”, “acts”, “album”, “alto”, “amusement”, “animal”, “animals”, “anthropologists”, “anthropology”, “archaeological”, “archaeology”, “art history”, “art”, “artifacts”, “arts”, “asian”, “audience”, “audio”, “auditorium”, “band”, “bands”, “banquet”, “banquets”, “baroque”, “bass”, “behavior”, “bourdieu”, “brass”, “camera”, “camping”, “cantatas”, “cappella groups”, “cappella music”, “cappella”, “cello”, “century”, “ceremonies”, “ceremony”, “change”, “choir”, “choirs”, “choral music”, “choral”, “chorus”, “christian”, “church”, “churches”, “cinema”, “circus”, “circuses”, “clarinet”, “class”, “classical music”, “classical”, “college”, “collegiate cappella”, “comedy”, “community”, “competitions”, “composers”, “concert band”, “concert”, “concerts”, “conductor”, “conflict”, “consists”, “continental”, “cooking”, “corps”, “court”, “courts”, “cultural capital”, “cultural diversity”, “cultural heritage”, “cultural relativism”, “cultural”, “culture”, “cultures”, “cycling”, “dance”, “dancers”, “dances”, “distance”, “doubling”, “drama”, “drum”, “education”, “effects”, “ensemble”, “ensembles”, “entertainment”, “episodes”, “fair”, “fairs”, “fan”, “fandom”, “fans”, “fashion”, “feast”, “festival”, “festivals”, “fiction”, “film festival”, “film festivals”, “film”, “films”, “folds”, “folk music”, “folk”, “folklore”, “food”, “friendships”, “fun”, “game”, “games”, “greek”, “group”, “groups”, “guests”, “guitar”, “habitus”, “hairstyles”, “hall”, “halls”, “harmony”, “having fun”, “heritage”, “history”, “home cinema”, “home”, “horn”, “human”, “individual”, “individuals”, “instrument”, “instrumental”, “instruments”, “jazz”, “leisure”, “lgbt”, “literature”, “magic”, “magician”, “magicians”, “maori”, “marx”, “masques”, “media”, “members”, “modern”, “motets”, “multiple aisle”, “museology”, “museum”, “museums”, “music ensembles”, “music festivals”, “music hall”, “music”, “musical instruments”, “musical”, “musicians”, “myth”, “myths”, “national”, “network”, “norm”, “norms”, “opera”, “operas”, “operetta”, “oral literature”, “orchestra”, “orchestras”, “organ”, “palace”, “parade”, “parties”, “parts”, “party”, “percussion”, “perform”, “performance”, “performed”, “performers”, “philosophy”, “photography”, “piano”, “play”, “player”, “players”, “playing”, “plays”, “political”, “polyphony”, “pop”, “popular culture”, “popular music”, “popular”, “quartet”, “quintet”, “race”, “races”, “racing”, “recreation”, “recreational”, “religion”, “religions”, “religious”, “remix”, “renaissance”, “restoration”, “ritual”, “rock”, “role”, “roles”, “roman”, “royal”, “running”, “sacred”, “satire”, “saxophone”, “school”, “schools”, “science”, “scientific”, “season”, “series”, “sing”, “singers”, “singing”, “social class”, “social innovation”, “social stratification”, “social”, “socialization”, “societies”, “society”, “sociology”, “song”, “songs”, “soprano”, “sound”, “south asian”, “spectacle”, “spectator”, “sport”, “sports”, “sprint finishes”, “sprint”, “stage”, “stratification”, “string quartet”, “string”, “structure”, “style”, “styles”, “sung”, “symphony orchestra”, “symphony”, “tactic”, “television”, “theater”, “theaters”, “theatre”, “theatres”, “theory”, “time”, “tournament”, “tournaments”, “tradition”, “traditional”, “tragedy”, “unesco”, “university”, “venue”, “venues”, “video game”, “video games”, “video”, “viola”, “violins”, “vocal group”, “vocal”, “voice”, “voices”, “western”, “woodwind”, “working class”, “worship”, “writing”, “youth”, “hobby”, “hobbies”, # Attractions New York "383 Madison Avenue", "40 Wall Street", "9/11 Tribute Museum", "92nd Street Y", "Abe Lebewohl Park", "Abingdon Square Park", "Adventurer’s Park", "African Burial Ground National Monument", “Alben Square”, "Albert Capsouto Park", "Alice Austen House", "Alice Tully Hall", “Alley Park”, "Alley Pond Park", "American Folk Art Museum", "American International Building", "American Museum of Immigration", "American Museum of Natural History", "America’s Response Monument", “Amundsen Circle”, "Andrew Heiskell Braille and Talking Book Library", "Anthony Catanzaro Square", “Apollo Theater”, “Aqueduct Walk”, "Arthur W. Diamond Law Library", “Ascenzi Square”, “Asphalt Green”, "Asser Levy Recreation Center", “Astoria Park”, "Avery Architectural and Fine Arts Library", "Baisley Pond Park", "Bank of America Tower", “Bargemusic”, "Barnum’s American Museum", "Barretto Point Park", "Bartow-Pell Mansion", “Battery Park”, "Battery Park City Ferry Terminal", "Bayswater Point State Park", “Beech tree”, "Bellevue South Park", “Belvedere Castle”, “Bennett Park”, “Bensonhurst Park”, "Betsy Head Park", "Billie Holiday Theatre", “Binghamton University”, "Blackwell Island Light", “Bloomberg Tower”, “Bloomingdale Park”, "Blue Heron Park", "Bocchino-Dente Memorial Plaza", “Boone Park”, “Bowery Ballroom”, “Bowling Green”, “Bowne Park”, “Bridge Park”, “Brill Building”, “Broadway theatre”, "Bronx Library Center", "Bronx Museum of the Arts", “Bronx Park”, "Bronx Skate Park", “Bronx Zoo”, “Brookfield Place”, "Brooklyn Academy of Music", "Brooklyn Botanic Garden", “Brooklyn Bridge”, "Brooklyn Bridge Park", "Brooklyn Children’s Museum", "Brooklyn Heights Promenade", “Brooklyn Museum”, "Brooklyn Navy Yard", "Brooklyn Public Library", "Brooklyn Symphony Orchestra", "Brooklyn–Queens Greenway", “Brower Park”, “Bryant Park”, “Buono Beach”, "Burton Arms Apartments", "Bush Terminal Park", "Bushwick Inlet Park", “Butler Library”, "C.V. Starr East Asian Library", “Cadman Plaza”, "Calvert Vaux Park", "campus of New York University", "Captain Patrick J. Brown Walk", "Captain Tilly Park", "Carl Schurz Park", “Carnegie Hall”, "Carnegie Hall Tower", “Carroll Park”, “Castle Clinton”, "Cathedral of St. John the Divine", "Catholic War Veterans Triangle", “Central Park”, "Central Park Zoo", "Charlie’s Place", "Chelsea Art Museum", “Chelsea Park”, “Chelsea Piers”, "Chief Charles A. Joshua Plaza", "Children’s Museum of the Arts", "Choco-Story New York", "Chris Postiglione Triangle", “Chrysler Building”, “Citi Field”, “Citigroup Center”, "City Hall Park", "City Parks Foundation", “CitySpire Center”, “Claremont Park”, "Clay Pit Ponds State Park Preserve", "Cleopatra’s Needle", “Climate Museum”, “Clinton Hall”, "Cloisters, The", "Clove Lakes Park", "Cobble Hill Park", “Collect Pond”, "College Point Fields", "College Point Little League Building", “Columbia University”, “Columbus Circle”, “Columbus Park”, "Commodore Barry Park", "Con Edison Energy Museum", "Condé Nast Building", "Concrete Plant Park", "Conference House Park", “Conference House”, “Conservatory Garden”, "Coney Island Creek Park", "Coney Island Cyclone", “Cooper Park”, "Cooper-Hewitt, National Design Museum", “Corlears Hook”, "Craftsman Music Center", “Crocheron Park”, “Crotona Park”, “Cunningham Park”, "Dag Hammarskjold Library", "Dag Hammarskjold Plaza", “Damrosch Park”, “Dante Park”, "David Geffen Hall", "David H. Koch Theater", “DeSalvio Playground”, "DeWitt Clinton Park", "Diana Ross Playground", "Discovery Times Square Exposition", “Domino Park”, “Doughboy Park”, “Douglaston Park”, "Downtown Athletic Club", “Drumgoole Plaza”, “Duane Park”, “Dyckman House”, "Dyker Beach Park", "Dyker Beach Park and Golf Course", "East Coast Memorial", "East River Esplanade", "East River Greenway", "East River Park", "East River State Park", “Eastern Parkway”, "Edgar Allan Poe Cottage", "Eleanor Roosevelt Monument", "Elizabeth H. Berger Plaza", “Ellis Island”, "Elmer Holmes Bobst Library", “Elmhurst Park”, "Empire State Building", "Estella Diggs Park", "Far Rockaway Skate Park", "Father Demo Square", “Federal Hall”, “Feehan Triangle”, "Ferry Point Park", “Film Forum”, "Film Society of Lincoln Center", "Firemen’s Memorial", "Fisher Landau Center", “Flatiron Building”, “Flushing Fields”, "Flushing Meadows-Corona Park", "Floyd Bennett Field", "Foch Sitting Area", “Foley Square”, “Forbes Galleries”, “Fordham University”, “Forest Park”, "Fort George Amusement Park", "Fort Greene Park", "Fort Hill Park", "Fort Schuyler, Bronx", “Fort Tilden”, “Fort Totten”, "Fort Tryon Park", “Fort Wadsworth”, "Fort Washington Park", "Franklin D. Roosevelt Four Freedoms Park", “Freshkills Park”, "Frick Art Reference Library", “Frick Collection”, “Fulton Park”, “GE Building”, "Gantry Plaza State Park", "Gateway National Recreation Area", "General Grant National Memorial", "George Gustav Heye Center", "George Washington Bridge", "Givans Creek Woods", "Golconda Skate Park", “Gorman Park”, “Gottesman Libraries”, “Governors Island”, "Governors Island National Monument", “Gracie Mansion”, “Gramercy Park”, "Grand Army Plaza", "Grand Central Terminal", "Graniteville Quarry Park", "Great Kills Park", "Great Lawn and Turtle Pond", "Green-Wood Cemetery", “Greenacre Park”, "Guggenheim Museum SoHo", "Hall of Fame for Great Americans", "Hamilton Fish Park", "Hamilton Grange National Memorial", “Hammerstein Ballroom”, “Hanover Square”, "Harlem River Park", “Hart Island”, "Harvard Club of New York City", “Heckscher Playground”, "Hell’s Kitchen Park", "Hendrick I. Lott House", "Henry Hudson Park", “Herald Square”, "Herbert Von King Park", “High Line”, “Highbridge Park”, “Highland Park”, "Hillcrest Veterans Square", "Historic Richmond Town", “Hoffman Island”, “Holland Tunnel”, “Hudson Boulevard”, "Hudson Park and Boulevard", "Hudson River Park", “Hunter Island”, "Hunts Point Riverside Park", "Ilka Tanya Payán Park", “Imagination Playground”, "Imagination Playground at Burling Slip", “Ingram Woods”, "International Center of Photography", "International Freedom Center", "Intrepid Sea, Air & Space Museum", "Inwood Hill Park", "Irish Hunger Memorial", “Isham Park”, "Isle of Meadows", "J.J. Byrne Park", "J. Hood Wright Park", "Jacob Riis Park", "Jackie Robinson Park", "Jackson Square Park", “Jamaica Bay”, "Jamaica Bay Park", "Jamaica Bay Wildlife Refuge", "Jazz at Lincoln Center", “Jerome Park”, “Jewish Museum”, "Joe Sabba Park", "John J. Carty Park", "John Jay Park", "John Paul Jones Park", "Joseph Rodman Drake Park", "Joyce Kilmer Park", "Judge Moses Weinstein Playground", “Juilliard School”, "Julio Carballo Fields", "Juniper Valley Park", “Kaiser Park”, "Kaufman Music Center", “KeySpan Park”, “King Manor”, “Kingsland Homestead”, “Kissena Park”, “Kohlreiter Square”, “Koptyko Triangle”, "Kurdish Heritage Foundation of America", "La MaMa Experimental Theatre Club", "Last Chance Pond Park", "Lefferts Historic House", "Leif Ericson Park", “Lemon Creek”, "Leon S. Kaiser Playground", "Lewis H. Latimer House", “Libra Triangle”, “Liberty Island”, “Liberty Park”, "Lincoln Center for the Performing Arts", “Lincoln Tunnel”, “Linden Park”, “Lipstick Building”, "Little Island at Pier 55", "Little Red Lighthouse", “LoCicero Triangle”, "Long Pond Park", "Louis Cuvillier Park", "Lower East Side Tenement Museum", “Lynch Triangle”, "Lyons Pool Recreation Center", “MacArthur Playground”, "Macombs Dam Park", “Macri Triangle”, "Macy’s Herald Square", “Madison Square”, "Madison Square Garden", “Mafra Park”, “Magenta Playground”, “Manhattan Bridge”, "Manhattan Municipal Building", "Manhattan School of Music", "Manhattan Waterfront Greenway", "Mannes College of Music", "Marcus Garvey Park", "Maria Hernandez Park", “Marine Park”, "Marsha P. Johnson State Park", “Martinez Playground”, “McCarren Park”, “McGolrick Park”, “McKenna Square”, "Merchant’s House Museum", “Met Breuer”, “MetLife Building”, "Metropolitan Fireproof Warehouse", "Metropolitan Museum of Art", “Metropolitan Opera”, “Midland Beach”, “Midtown Manhattan”, "Mill Pond Park", “Mill Rock”, "Mill Rock Park", “Millennium Park”, “Miller Field”, "Mitchel Square Park", "Monsignor McGolrick Park", “Montefiore Square”, "Morbid Anatomy Museum", "Morgan Library & Museum", “Morningside Park”, "Morris-Jumel Mansion", “Mosholu Parkway”, "Mount Loretto Unique Area", "Mount Prospect Park", “Mullaly Park”, "Municipal Asphalt Plant", “Muscota Marsh”, "Museum of American Finance", "Museum of Arts and Design", "Museum of Biblical Art", "Museum of Chinese in America", "Museum of Comic and Cartoon Art", "Museum of Food and Drink", "Museum of Jewish Heritage", "Museum of Modern Art", "Museum of Primitive Art", "Museum of the City of New York", "National Museum of Catholic Art and History", "National Museum of the American Indian", "National September 11 Memorial & Museum", "New Dorp Beach", “New Jersey”, "New York Aquarium", "New York Botanical Garden", "New York Chinese Scholar’s Garden", "New York City Ballet", "New York City Center", "New York City Hall", "New York City Opera", "New York Evening Post Building", "New York Hall of Science", "New York Jazz Museum", "New York Life Building", "New York Philharmonic", "New York Pops", "New York Public Library", "New York Public Library Main Branch", "New York Public Library for the Performing Arts", "New York Society Library", "New York Stock Exchange", "New York Tattoo Museum", "New York University", “Noguchi Museum”, "North and South Brother Islands", "North Woods and North Meadow", "O'Donohue Park", “Ocean Parkway”, "Odd Fellows Hall", "Old Stone House", "Onassis Cultural Center", "One Chase Manhattan Plaza", "One Room Schoolhouse Park", "One World Trade Center", "One Worldwide Plaza", "Open Road Park", “Orchard Beach”, "Owl’s Head Park", "Paley Center for Media", “Paley Park”, "Park of the Americas", "Pelham Bay Park", “Pelham Parkway”, “Pennsylvania Station”, “Peretz Square”, "Peter Detmold Park", “Petrosino Square”, "Pier 11/Wall Street", “Pier 42”, “Plaza Hotel”, “Plaza Lafayette”, “Playground 52”, "Playground Seventy Five", “Poets House”, "Prall’s Island", "Printer’s Park", "Prison Ship Martyrs’ Monument", "Proctor-Hopson Circle", “Prospect Park”, "Prospect Park Zoo", “Public Theater”, "Pugsley Creek Park", "Queen Elizabeth II September 11th Garden", "Queens Botanical Garden", "Queens County Farm Museum", "Queens Museum of Art", "Queens Public Library", “Queens Zoo”, “Queensbridge Park”, "Rachel Carson Playground", "Racquet and Tennis Club", "Radio City Music Hall", "Ralph Bunche Park", "Randalls and Wards Islands", "Raoul Wallenberg Forest", "Rare Book & Manuscript Library", "Red Hook Park", “Remsen Cemetery”, “Riegelmann Boardwalk”, "Ripley’s Believe It or Not!", "Riverbank State Park", “Riverdale Park”, “Riverside Park”, "Robert Moses Playground", "Roberto Clemente State Park", "Rock and Roll Hall of Fame", "Rockaway Beach and Boardwalk", "Rockaway Community Park", “Rockefeller Center”, "Rodman’s Neck", "Rose Center for Earth and Space", "Roundabout Theatre Company", "Roy Wilkins Park", “Rucker Park”, "Rufus King Park", "Russell D. Ramsey Triangle", "Sailors’ Snug Harbor", “Sakura Park”, "Samuel J. Friedman Theatre", "Sara Delano Roosevelt Park", “Saratoga Park”, "Schomburg Center for Research in Black Culture", "Science, Industry and Business Library", “Seagram Building”, “Seguine Mansion”, “Septuagesimo Uno”, "Seton Falls Park", "Seth Low Playground", “Seward Park”, “Shea Stadium”, “Sheridan Square”, “Sherman Square”, "Shevchenko Scientific Society", "Shirley Chisholm State Park", “Shooters Island”, "Shore Boulevard Mall", “Silver Lake”, “Silvercup Studios”, "Skirball Center for the Performing Arts", "Snug Harbor Cultural Center", "Society of Illustrators", "Socrates Sculpture Park", "Solomon R. Guggenheim Museum", "Sony Wonder Technology Lab", “Soundview Park”, "South Beach Boardwalk", "South Beach–Franklin Delano Roosevelt Boardwalk", "South Street Seaport", “Southpoint Park”, "Sphere, The", "Sports Museum of America", "Spring Creek Park", "Spring Street Park", “Springfield Park”, "St. James Park", "St. John’s Park", "St. Mary’s Park", "St. Nicholas Park", "St. Patrick’s Cathedral", "St. Vartan Park", “Starlight Park”, “Staten Island”, "Staten Island Botanical Garden", "Staten Island Greenbelt", "Staten Island Zoo", "Statue of Liberty", "Statue of Liberty National Monument", “Steeplechase Park”, "Stonewall National Monument", “Straus Park”, "Strawberry Fields (memorial)", "Stuyvesant Cove Park", “Stuyvesant Square”, “Sunnyside Gardens”, “Sunset Park”, "Swedish Cottage Marionette Theatre", “Swinburne Island”, “Symphony Space”, “Tammany Hall”, “Tappen Park”, "Taqwa Community Farm", "Tarr Family Playground", “Teardrop Park”, "Theodore Roosevelt Birthplace National Historic Site", "Theodore Roosevelt Park", "Thomas Jefferson Park", "Thomas Paine Park", “Times Square”, "Tompkins Square Park", “Town Hall”, “Travers Park”, “Travis Triangle”, “Tremont Park”, “Triangle 54”, "Tribute in Light", “Tribute Park”, “Trinity Church”, “Trinity Churchyard”, "Trump World Tower", "Trygve Lie Plaza", “Turtle Playground”, "Udall’s Park Preserve", “Udalls Cove”, "Underbridge Dog Run", “Union Square”, "United Nations Headquarters", “University Plaza”, “University Woods”, "Valentine-Varian House", "Van Cortlandt House", "Van Cortlandt Park", “Verdi Square”, “Vesuvio Playground”, "Vietnam Veterans Plaza", "Vincent F. Albano Jr. Playground", "Vinmont Veteran Park", "Vivian Beaumont Theater", “Vleigh Playground”, "WNYC Transmitter Park", “Wagner Park”, "Waldorf-Astoria Hotel", "Walt Whitman Park", "Walter J. Wetzel Triangle", "Washington Market Park", "Washington Square Park", “Wave Hill”, “Wayback Machine”, "Weeksville Heritage Center", "West Harlem Piers", "West Side Community Garden", "Whitney Museum of American Art", "William A. Harris Garden", "William T. Davis Wildlife Refuge", "Williamsburg Art & Historical Center", “Williamsburg Bridge”, “Williamsbridge Oval”, “Willowbrook Park”, "Winston Churchill Square", "Wolfe’s Pond Park", “Woodlawn Cemetery”, “Woolworth Building”, "World Trade Center", "Wyckoff-Bennett Homestead", “Yankee Stadium”, “Zion Triangle”, “Zuccotti Park”

### Curated keywords transportation

“aerial”, “air traffic”, “aircraft”, “airlines”, “airplanes”, “airport”, “airports”, “airspace”, “airways”, “autogyro”, “autogyros”, “automobile”, “aviation”, “barge”, “bicycle”, “bicycles”, “boat”, “boats”, “bus”, “buses”, “business travel”, “car”, “cargo”, “carriageway”, “cars”, “commuter rail”, “commuter”, “convention road”, “crossing”, “destination”, “diesel”, “driver”, “drivers”, “driving”, “electric locomotives”, “engine”, “engines”, “expressway”, “expressways”, “ferries”, “ferry”, “fixed wing”, “flight”, “flights”, “flying”, “freight”, “fuel”, “fuselage”, “glider”, “gliders”, “helicopter”, “helicopters”, “high speed”, “highway”, “infrastructure”, “inter city”, “interchange”, “intersection”, “intersections”, “jet”, “junction”, “kite”, “km”, “landing”, “lane”, “lanes”, “lift”, “light rail”, “locomotive”, “locomotives”, “metro”, “mile”, “monorail”, “monorails”, “motor”, “motorcycles”, “motors”, “mph”, “navigation”, “network traffic”, “overtaking”, “parking”, “passenger”, “passengers”, “pedestrian”, “pedestrians”, “pilot”, “pilots”, “plane”, “pollution”, “port”, “ports”, “propeller”, “public transport”, “public transportation”, “rail”, “railroad”, “railroads”, “rails”, “railway”, “railways”, “rapid transit”, “rapid”, “road signs”, “road traffic”, “road”, “roads”, “rotor”, “rotorcraft”, “rotors”, “roundabout”, “roundabouts”, “route”, “routes”, “ship”, “ships”, “signal”, “signals”, “spacecraft”, “speed rail”, “speed”, “stations”, “steam locomotives”, “stop sign”, “stop signs”, “submarine”, “submarines”, “subway”, “tail rotor”, “terminal”, “track”, “tracks”, “traffic control”, “traffic signs”, “traffic”, “trains”, “tram”, “tramway”, “transit”, “transport”, “transportation”, “transshipment”, “travel time”, “travel times”, “travel”, “trip”, “trips”, “underpasses”, “vehicle”, “vehicles”, “vessels”, “wagons”, “watercraft”, “waterway”, “wing aircraft”, “driving”

### Curated keywords retail activity

Ad blocking", “ad server”, “ad space”, “ad”, “ads”, “advertisements”, “advertisers”, “advertising”, “amazon”, “appliance”, “appliances”, “automated customer”, “banking”, “banner ads”, “banners”, “based pricing”, “booksellers”, “bookstore”, “bookstores”, “brand”, “bulk”, “bundle”, “bundles”, “bundling”, “business users”, “business”, “businesses”, “buy free”, “buy”, “buyers”, “capita”, “care products”, “cents”, “chain”, “chains”, “cloth”, “clothing”, “commerce”, “commercial”, “commercials”, “compensation”, “competitive”, “consumer protection”, “consumer spending”, “consumer”, “consumers”, “consumption”, “convenience store”, “convenience stores”, “convenience”, “cookies”, “cosmetic”, “cosmetics”, “cost”, “costs”, “customer service”, “customer support”, “customer”, “customers employees”, “customers financial”, “customers”, “delivered”, “delivery”, “demand”, “department store”, “department stores”, “display ads”, “display advertising”, “disposable”, “distribution”, “economic”, “economy”, “edlp”, “electric”, “electronic commerce”, “equipment”, “exchange”, “fabric”, “financial institutions”, “footwear”, “fraud”, “furniture”, “gdp”, “gift”, “gni”, “good”, “goods services”, “goods”, “grocery stores”, “grocery”, “growth”, “guarantee”, “guarantees”, “half price”, “handicraft”, “handicrafts”, “hypermarket”, “hypermarkets”, “industry”, “institutional customers”, “institutional”, “institutions”, “investment”, “items”, “letterpress”, “local”, “lumber”, “manufacturing”, “market”, “marketing mix”, “marketing”, “markets”, “materials”, “merchandise”, “mobile advertising”, “money guarantee”, “monger”, “net”, “nrf”, “offer”, “online ads”, “online advertising”, “online shopping”, “order”, “pay”, “payment”, “peddler”, “peddlers”, “peddling”, “plastic”, “price”, “prices”, “pricing”, “product”, “production”, “products services”, “products”, “profit”, “promotion”, “ps”, “psychological pricing”, “purchase”, “purchases”, “rate”, “rates”, “rbp”, “retail”, “retailers”, “revenue”, “sale”, “sales”, “salesman”, “sell”, “selling”, “serve”, “service consumer”, “service delivery”, “service environment”, “service experience”, “service quality”, “service”, “services”, “servicescape”, “servicescapes”, “shoes”, “shop”, “shoppers”, “shopping”, “shops”, “single use”, “software”, “spam”, “spend”, “spending”, “sponsored”, “stationers”, “store”, “stores”, “supermarket”, “supermarkets”, “supplies”, “supply chain”, “supply”, “tax”, “textile”, “textiles”, “transaction”, “transactions”, “unit”, “value based”, “value”, “vat”, “vending machine”, “vending machines”, “vending”, “wholesale”, “wholesalers”, “wholesaling”, “discount”, “discounts”.

### Curated keywords work/remote work

“abolition work”, “american worker”, “anti trafficking”, “anti work”, “apprentice”, “apprentices”, “apprenticeship”, “apprenticeships”, “basic income”, “benefits”, “bullshit jobs”, “care labor”, “care work”, “career”, “child labor”, “child labour”, “children work”, “collar workers”, “collar”, “companies”, “company”, “corporate”, “corporations”, “coworking spaces”, “coworking”, “craftsman”, “craftsmen”, “critique work”, “degree”, “directors”, “division labour”, “domestic work”, “double burden”, “employed”, “employee engagement”, “employee”, “employees”, “employer”, “employers”, “employment”, “forced labour”, “gdp”, “gpi”, “graeber”, “hour shifts”, “hours week”, “housework”, “human computers”, “human trafficking”, “income”, “isco”, “job characteristics”, “job guarantee”, “job satisfaction”, “job”, “jobs”, “journeymen”, “labor force”, “labor”, “labour force”, “labour market”, “labour”, “limited liability”, “management”, “managers”, “manual labor”, “master craftsman”, “master craftsmen”, “meister”, “oecd”, “office”, “offices”, “overwork”, “pension”, “productivity”, “profession”, “professional”, “professionals”, “professions”, “protestant work”, “qualification”, “remote work”, “retire”, “retirement”, “seniority”, “sex trafficking”, “sex work”, “sex workers”, “shareholders”, “shift”, “shifts”, “slave trade”, “slave”, “slavery”, “slaves”, “social security”, “standard working”, “tasks”, “technician”, “tradesman”, “traffickers”, “trafficking victims”, “trafficking”, “turnover”, “unemployed”, “unemployment benefits”, “unemployment insurance”, “unemployment rate”, “unemployment”, “unpaid care”, “unpaid domestic”, “unpaid labor”, “unpaid work”, “unpaid”, “value unpaid”, “voluntary”, “wage labour”, “wage”, “wages”, “white collar”, “work essays”, “work ethic”, “work family”, “work week”, “work”, “worked”, “worker”, “workers”, “working class”, “working hours”, “working time”, “working”, “home office”, “wfh”, "working from home"
